# Supplementary material for: Regiospecific α-methylene functionalisation of tertiary amines with alkynes via Au-catalysed concerted one-proton/two-electron transfer to O2
Source: Nat Commun. 2022 Nov 9;13:6505. doi: 10.1038/s41467-022-34176-x (PMC9646731; doi:10.1038/s41467-022-34176-x)
Supplement: Supplementary file 1 — Supplementary Information [file 41467_2022_34176_MOESM1_ESM.pdf]

Supplementary Information

Regiospecific α-methylene functionalisation of tertiary amines with alkynes via Au-catalysed concerted one-proton/two-electron transfer to O_2

Takafumi Yatabe[1*] and Kazuya Yamaguchi[1*]

[1]Department of Applied Chemistry, School of Engineering, The University of Tokyo, 7-3-1 Hongo, Bunkyo-ku, Tokyo 113-8656, Japan

[*]e-mail: kyama@appchem.t.u-tokyo.ac.jp, yatabe@appchem.t.u-tokyo.ac.jp

Table of Contents

Supplementary Methods **2–23**

Supplementary Tables **24–29**

Supplementary Figures **30–96**

Supplementary References **97–99**

Supplementary Methods

Instruments and Reagents: Gas chromatography (GC) analyses were performed on a Shimadzu GC-2014 instrument with a flame ionization detector (FID) equipped with a TC-5 capillary column. GC-mass spectrometry (MS) spectra were recorded on a Shimadzu GCMS-QP2010 instrument equipped with an InertCap5 capillary column at an ionization voltage of 70 eV. Liquid-state ^{1}H, ^{13}C, and ^{19}F NMR spectra were recorded on a JEOL JNM-ECA 500 instrument. ^{1}H and ^{13}C NMR spectra were collected at 500.16 and 125.77 MHz, respectively. ^{1}H and ^{13}C NMR spectra were calibrated by tetramethylsilane (TMS) as the internal standard ($\delta = 0$ ppm) or the solvent peak (^{1}H NMR using toluene-d_8: $\delta = 7.01$ ppm, ^{13}C NMR using toluene-d_8: $\delta = 20.43$ ppm, $CDCl_3$: $\delta = 77.16$ ppm). ^{19}F NMR was measured at 470.62 MHz with benzotrifluoride as an external standard ($\delta = -63.72$ ppm). The desired products were isolated using Biotage Isolera. Inductively coupled plasma atomic emission spectroscopy (ICP-AES) analyses were performed on a Shimadzu ICPS-8100 instrument. Transmission electron microscopy (TEM) measurements were performed on JEOL JEM-2000EX II. TEM samples were prepared by placing a drop of the suspension with EtOH on carbon-coated Cu grids and dried *in vacuo*. X-ray diffraction (XRD) patterns were recorded using a Rigaku SmartLab instrument under Cu Kα radiation (45 kV, 200 mA). X-ray photoelectron spectroscopy (XPS) measurements were carried out on a ULVAC-PHI PHI5000 VersaProbe instrument using the Al Kα radiation ($h\nu$ = 1486.6 eV). Infrared (IR) spectra were measured on Jasco FT/IR-4100 using KBr disks. Elemental analyses for C, H, and N were performed on Elementar vario MICRO cube. Pd supported on Al_2O_3 (Pd/Al_2O_3. Pd: 5wt%, Lot. No. 237-020410, N.E. CHEMCAT), Pt supported on Al_2O_3 (Pt/Al_2O_3. Pt: 5wt%, Lot. No. 137-90020, N.E. CHEMCAT), Ru supported on Al_2O_3 (Ru/Al_2O_3, Ru: 5wt%, Lot. No. 437-000050, N.E. CHEMCAT), $Mg_6Al_2(OH)_{16}(CO_3)\cdot 4H_2O$ (layered double hydroxide: LDH) (47 $m^2\,g^{-1}$, Cat. No. Tomita-AD 500, Tomita Pharmaceutical), $Ca_{10}(PO_4)_6(OH)_2$ (hydroxyapatite: HAP) (11 $m^2\,g^{-1}$, Cat. No. 011-14882, FUJIFILM Wako), ZnO (7 $m^2\,g^{-1}$ after calcination at 600 °C for 2 h, Cat. No. 265-00971, FUJIFILM Wako), CeO_2 (45 $m^2\,g^{-1}$ after calcination at 600 °C for 2 h, Cat. No. 544841-25G, Sigma-Aldrich), ZrO_2 (48 $m^2\,g^{-1}$ after calcination at 600 °C for 2 h, Cat. No. JRC-ZRO-6, Catalysis Society of Japan), Al_2O_3 (183 $m^2\,g^{-1}$ after calcination at 600 °C for 2 h, Cat. No. KHS-24, Sumitomo Chemical), and TiO_2 (66 $m^2\,g^{-1}$ after calcination at 600 °C for 2 h, Cat. No. ST-01, Ishihara Sangyo Kaisha) were commercially available. Solvents, substrates, and metal sources were obtained from Kanto Chemical, Tokyo Chemical Industry, FUJIFILM Wako, Sigma-Aldrich, or Alfa Aesar (reagent grade). Several substrates were purified by Kugelrohr distillation just before use.

Leaching Test: To establish whether the observed catalysis of the α-methylene-selective alkynylation of **1a** with **2a** was heterogeneous in nature and was affected by Au/HAP — and not by metal species leaching from Au/HAP into the reaction solution — Au/HAP was removed from the reaction mixture by hot filtration 2 h after the initiation of the reaction under the optimized conditions; the reaction was then carried forward with the filtrate under the same optimized conditions (Supplementary Fig. 4). Removal of the catalyst caused the production of **2a** to cease immediately, and the yield of **3aa** did not change at the end of the specified 24 h. In order to measure the amount of metals leached into solution, the filtrate in which the reaction had been carried out for 24 h was evaporated to dryness *in vacuo*, and the residue obtained was treated with concentrated aqua regia (1 mL), and sonicated. The amounts of Au and Ca in the filtrate were then determined by ICP-AES after the solution was filtered off and moved into a 10 mL volumetric flask. The results indicated that gold and calcium were hardly detected in the reaction mixture (Au: 0.30% and Ca: 0.054% of Au and Ca used for the reaction, respectively). Therefore, the observed catalysis of Au/HAP for this reaction was confirmed to be truly heterogeneous. On the other hand, in the filtrate, a substantial amount of Zn species was detected by ICP-AES (27% of Zn used for the reaction), which was consistent with the role of Zn species as the homogeneous cocatalyst for nucleophilic addition.

Reuse Test: After the reaction, Au/HAP was retrieved from the reaction mixture by simple filtration using an Omnipore membrane filter. The retrieved catalyst was washed with CH_3CN (20 mL for every 100 mg of Au/HAP), and dried *in vacuo*. The catalyst thus retrieved was utilized to conduct reuse experiments. Consequently, Au/HAP can be reused at least twice for the reaction of **1a** and **2a** without lowering the yields of **3aa** as of 24 h, although the reaction rate appeared to decrease as the number of reuses increased (Supplementary Table 3 and Supplementary Fig. 5). FT-IR spectra showed no obvious difference between fresh Au/HAP and the used Au/HAP (Supplementary Fig. 6). XRD patterns of the used Au/HAP indicated that the structure of HAP had not changed after the reaction, whereas the gold nanoparticle size had increased slightly (Supplementary Fig. 7). In fact, TEM images of the used catalysts shown in Supplementary Fig. 8 clarified the aggregation of Au nanoparticles after the reactions (mean diameter of before use: 5.1 nm, mean diameter after the 1st use: 9.0 nm, mean diameter after the 3rd use: 10.5 nm). Furthermore, the presence of Zn species in the used Au/HAP was confirmed by XPS (Supplementary Fig. 9). Considering these evidences, the apparent progressive deactivation of the catalyst as a consequence of reuse is likely the result of an increase in the size of the gold nanoparticle and/or of the attachment of Zn species onto Au/HAP.

O_2 Pressure Dependence on Production Rates: Experiments of O_2 pressure dependence on production rates of **3aa** were carried out as follows. Into a schlenk flask, an internal standard (biphenyl, 0.1 mmol), substrates (**1a**: 0.5 mmol, **2a**: 0.5 mmol), Au/HAP (100 mg, 1.5 mol%), $ZnBr_2$ (10 mol%), and $PhCF_3$ solvent (2 mL) were added. After the flask and the solution were degassed by freeze-pump-thaw cycling, the reaction started under an atmosphere of O_2/N_2 mixed gas balloon with controlled O_2 partial pressures using flow meters. The solutions sampled every 10 min for 40 min were analyzed by GC.

Use of Deuterated Amine HCl Salts (Example: Kinetic Isotope Effect Using 1a-*d*4): 1-Methylpiperidine-2,2-*d*2 (**1a-*d*2**) and 1-methylpiperidine-2,2,2,2-*d4* (**1a-*d*4**) were synthesized as the HCl salts, thus, before the use of them, neutralization using NaOH in H_2O or NaOD in D_2O, extraction with solvent used for reactions, dehydration, and filtration were conducted. As a typical procedure of using the deuterated amines, an experiment of kinetic isotope effects using **1a-*d*4** on the present α-alkynylation with **2a** was shown below. First, the HCl salt of **1a-*d*4** (0.3 mmol) was added into a vial. Then, into the vial, an aqueous solution of 2M NaOH (150 μL) and $PhCF_3$ (0.5 mL×3) were added to extract **1a-*d*4** in the $PhCF_3$. The collected extraction solution was dried over Na_2SO_4 (100 mg). Then, filtration of the solids and washing with $PhCF_3$ (0.25 mL) gave the solution of **1a-*d*4**. After the addition of **2a** (1 mmol) and biphenyl (0.1 mmol) to the solution, GC analysis was performed to check the initial amount of **1a-*d*4** and **2a**. Into a Pyrex glass test tube, $ZnBr_2$ (5 mol% to **2a**), Au/HAP (Au: 0.8 mol% to **2a**), a Teflon-coated magnetic stir bar, and the prepared solution (containing **1a-*d*4**, **2a**, and biphenyl) were added. The mixture was stirred at 95 °C under an open air (1 atm). The solutions sampled every 10 min for 40 min were analyzed by GC to determine the yields of **3aa-*d*3**. As for the counterpart (**1a**), an HCl salt of **1a** was used in the same manner, and the production rate of **3aa** was determined.

DFT Calculations: All calculations were performed using the Gaussian 16 Rev B.01 or Rev. C software[S1]. Geometry optimizations and single-point energy calculations were conducted using the B3LYP functional[S2,S3] or M06[S4] functional with SDD[S5] basis sets for Au and 6-31G(d,p)[S6] basis sets for the other elements. For the spin multiplicity, all structures were calculated as the singlet state. All thermodynamic data were calculated at the standard state (25 °C and 1 atm).

Synthesis of *N*-methyl tertiary amines: The *N*-methylamines described below were prepared by reductive amination according to literature procedures.[S7] In particular, a secondary amine (10 mmol) was added into the mixture of a formaldehyde solution (37%, 40 mmol) and formic acid (40 mmol) at

room temperature. After the resulting mixture was refluxed for 24 h and cooled to room temperature, it was added to an aqueous solution of NaOH (2 M, 50 mL) and extracted with diethyl ether. The organic layers were washed with brine and dried over K_2CO_3. After the filtration of K_2CO_3, the solvent was evaporated under reduced pressure, and the residue was purified by distillation, affording the desired *N*-methylamine.

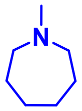

1c (CAS No. 1192-95-6)

1-methylazepane (1c): ^{1}H NMR (500 MHz $CDCl_3$, TMS): δ 1.59–1.69 (m, 8H), 2.35 (s, 3H), 2.53–2.56 (m, 4H). MS (70 eV, EI): *m/z* (%): 113 (51) [M^+], 112 (28), 98 (14), 85 (8), 84 (100), 71 (34), 70 (34), 58 (27), 57 (46), 56 (5), 55 (7).

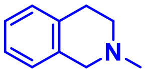

1e (CAS No. 1612-65-3)

2-methyl-1,2,3,4-tetrahydroisoquinoline (1f): ^{1}H NMR (500 MHz $CDCl_3$, TMS): δ 2.45 (s, 3H), 2.68 (t, J = 6.0 Hz, 2H), 2.92 (t, J = 6.0 Hz, 2H), 3.58 (s, 2H), 7.00–7.02 (m, 1H), 7.09–7.14 (m, 3H). MS (70 eV, EI): *m/z* (%):147 (45) [M^+], 146 (100), 144 (11), 131 (7). 115 (6), 105 (8), 104 (55), 103 (16), 78 (16), 77 (11), 73 (6), 51 (5).

Synthesis of *trans-N,N*-dimethyl-1-(2-phenylcyclopropyl)methanamine (1s): The amine substrate of a radical clock for HAT (**1q**) was synthesized referring to literature procedures.[S8] *trans*-2-Phenylcyclopropane-1-carbocylic acid (2.5 mmol), 4-dimethylaminopyridine (0.25 mmol), dry dichloromethane (8 mL), dimethylamine (5 mmol, 2M THF solution), and Et_3N (2.5 mmol) were successively added into a Schlenk flask. After cooling the solution to 0 °C, EDAC·HCl (3 mmol, 1-ethyl-3-(3-dimethylaminopropyl)carbodiimide hydrochloride) was added and stirred for 2 h at 0 °C. Then, the reaction solution was additionally stirred for 24 h at room temperature. The resulting solution was extracted with EtOAc (30 mL) and sat. citric acid aqueous solution (50 mL) followed by sat. $NaHCO_3$ aqueous solution (2×25 mL) and deionized water (30 mL). After drying the organic layer using Na_2SO_4 and evaporating the organic solvent *in vacuo*, the crude product was subjected to column chromatography on silica gel using EtOAc/hexane = 4/6 as an eluent, affording *trans-N,N*-dimethyl-2-phenylcyclopropane-1-carboxamide (0.261 g). Then, all the amides were added at 0 °C to a Schlenk flask containing $LiAlH_4$ (4 mmol) and dry THF (15 mL). After stirring the solution for 2 h, the reaction was quenched by deionized water and 2M NaOH aqueous solution. After filtration of

white solids with celite, washing by dry THF (10 mL), and drying on Na_2SO_4, the solution was evaporated *in vacuo* to afford the desired product, *trans*-*N*,*N*-dimethyl-1-(2-phenylcyclopropyl)methanamine (**1s**) (96.4 mg).

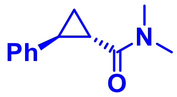

(CAS No. 5279-83-4)

***trans*-*N*,*N*-dimethyl-2-phenylcyclopropane-1-carboxamide:** ^{1}H NMR (500 MHz $CDCl_3$, TMS): δ 1.25–1.30 (m, 1H), 1.62–1.66 (m, 1H), 1.97–2.01 (m, 1H), 2.46–2.50 (m, 1H), 2.99 (s, 3H), 3.13 (s, 3H), 7.11–7.13 (m, 2H), 7.18–7.21 (m, 1H), 7.26–7.29 (m, 2H). MS (70 eV, EI): *m/z* (%): 189 (53) [M^+], 145 (20). 144 (34), 127 (26), 117 (56), 116 (32), 115 (52), 98 (12), 91 (26), 72 (100), 68 (26), 65 (11).

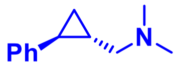

1s (CAS No. 16583-42-9)

***trans*-*N*,*N*-dimethyl-1-(2-phenylcyclopropyl)methanamine (1s):** ^{1}H NMR (500 MHz $CDCl_3$, TMS): δ 0.81–0.85 (m, 1H), 0.95–0.99 (m, 1H), 1.19–1.26 (m, 1H), 1.67–1.70 (m, 1H), 2.28 (dd, J = 12.6 and 6.3 Hz, 1H), 2.29 (s, 6H), 2.40 (dd, J = 12.6 and 6.3 Hz, 1H), 7.05–7.07 (m, 2H), 7.12–7.15 (m, 1H), 7.23–7.26 (m, 2H). MS (70 eV, EI): *m/z* (%): 175 (2) [M^+], 134 (13), 129 (7), 115 (7), 91 (12), 84 (10), 71 (79), 58 (100), 56 (12).

Synthesis of ethyl 1-(diethylamino)cyclopropane-1-carboxylate (1t): According to the reference,[S9] ethyl 1-(diethylamino)cyclopropane-1-carboxylate (**1t**) was synthesized as follows. Into a brown vial (volume: ~20 mL), K_2CO_3 (1.8 g, 13.1 mmol), 1-aminocyclopropanecarboxylic acid ethyl ester hydrochloride (0.36 g, 2.2 mmol), CH_3CN solvent (10 mL), and EtI (1.2 g, 7.9 mmol) were added and stirred at room temperature for 84 h. The resulting slurry was filtrated and washed with diethyl ether (15 mL). The solvent of the filtrate was evaporated, and then the residue was subjected to column chromatography on amine-modified silica gel using hexane/EtOAc = 9/1 as an eluent. After the column chromatography, evaporation of the desired fractions afforded **1t** (82.2 mg).

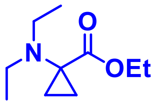

1t

ethyl 1-(diethylamino)cyclopropane-1-carboxylate (1t): ^{1}H NMR (500 MHz $CDCl_3$, TMS): δ 0.97 (dd, J = 7.3 and 4.0 Hz, 2H), 1.03 (t, J = 7.3 Hz, 6H), 1.25 (t, J = 7.3 Hz, 3H), 1.27 (dd, J = 7.5 and 4.0 Hz, 2H), 2.88 (q, J = 7.3 Hz, 4H), 4.11 (q, J = 7.1 Hz, 2H). ^{13}C–{^{1}H} NMR (125 MHz, $CDCl_3$, TMS): δ 14.3, 15.4, 19.1, 45.1, 47.8, 60.1, 175.1. MS (70 eV, EI): *m/z* (%): 185 (9) [M^+], 170 (8), 157 (7), 156 (66), 142 (7), 128 (8), 112 (33), 98 (5), 96 (6), 85 (7), 84 (100), 83 (8), 82 (13), 73 (11), 70 (8), 68 (11), 57 (6), 56 (100), 55 (20), 54 (28).

Synthesis of 1-methylpiperidine-2,2-*d*₂ (1a-*d*₂): By referring to the previous report,[S8] 1-methylpiperidine-2,2-d_2 (**1a-d_2**) was synthesized as the corresponding HCl salt through amide reduction. 1-Methylpiperidin-2-one (2 mmol) was added at 0 °C to a Schlenk flask containing $LiAlD_4$ (6 mmol) and dry THF (20 mL). After stirring the solution for 2 h, the reaction was quenched by deionized water and 2M NaOH aqueous solution. After filtration of white solids with celite and washing by dry THF (10 mL), the filtrate was dried over K_2CO_3 and Na_2SO_4 overnight. After filtration and partial evaporation, an aqueous solution of 1M HCl (5 mL) was added to the solution. The resulting solution was evaporated *in vacuo* using azeotrope with toluene several times to afford the desired HCl salt (193.2 mg). ^{1}H NMR of **1a-d_2** was measured in toluene-d_8 after treatment with NaOD/D_2O (40wt%) and filtration, determining the deuteration ratio (>95%).

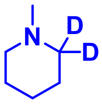

1a-d_2 (CAS No. 695-72-7)

1-methylpiperidine-2,2-d_2 (1a-d_2): ^{1}H NMR (500 MHz, toluene-d_8): δ 1.23–1.31 (m, 2H), 1.47–1.52 (m, 4H), 2.09 (s, 3H), 2.10–2.22 (m, 2H).

Synthesis of 1-methylpiperidine-2,2,2,2-d_4 (1a-d_4): After *N*-methylglutarimide synthesis,[S10] reduction of the imide with $LiAlD_4$ afforded 1-methylpiperidine-2,2,2,2-d_4 (**1a-d_4**) as follows.[S8] Glutarimide (1 g), K_2CO_3 (2.1 g), and CH_3I (2.13 g) were mixed in dry acetone (20 mL). Then, the reactions slurry was stirred under reflux for 24 h. After cooling, filtration, and evaporation of acetone, the residue was subjected to column chromatography on silica gel with an eluent of hexane/EtOAc = 6/4, affording *N*-methylglutarimide (0.77 g). Then, the imide (2 mmol) was added at 0 °C to a Schlenk flask containing $LiAlD_4$ (6 mmol) and dry diethyl ether (15 mL). After stirring the solution for 19 h,

the reaction was quenched by deionized water and 2M NaOH aqueous solution. After filtration of white solids and washing by dry diethyl ether, the filtrate was dried over Na_2SO_4. After filtration, an aqueous solution of 12M HCl (0.2 mL) was added to the solution with stirring. The resulting solution was evaporated *in vacuo* using azeotrope with toluene several times to afford the desired HCl salt (94.9 mg). ^{1}H NMR of **1a-*d*$_4$** was measured in toluene-*d*$_8$ after treatment with NaOD/D_2O (40wt%) and filtration, determining the deuteration ratio (>95%).

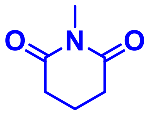

(CAS No. 25077-25-2)

***N*-methylglutarimide:** ^{1}H NMR (500 MHz, $CDCl_3$): δ 1.96 (quin, J = 6.5 Hz, 2H), 2.67 (t, J = 6.5 Hz, 4H), 3.14 (s, 3H). MS (70 eV, EI): m/z (%): 128 (7), 127 (100) [M^+], 99 (10), 98 (27), 71 (17), 70 (29), 58 (12), 56 (6), 55 (19).

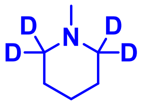

1a-*d*$_4$ (CAS No. 42983-61-9)

1-methylpiperidine-2,2,2,2-*d*$_4$ (1a-*d*$_4$): ^{1}H NMR (500 MHz, toluene-*d*$_8$): δ 1.22–1.34 (m, 2H), 1.48 (t, J = 5.9 Hz, 4H), 2.09 (s, 3H).

Synthesis of phenylacetylene-*d* (2a-*d*): In the following manner referred to the previous report,[S11] phenylacetylene-*d* (**2a-*d***) was synthesized. Into a Pyrex glass test tube, phenylacetylene (3 mmol) and D_2O (5 mL) were added. After purging the air in the test tube with Ar, the mixture was stirred at room temperature for about 3 days. The resulting mixture was extracted by dichloromethane three times and dried over Na_2SO_4. After removing Na_2SO_4 by filtration, evaporation of the solvents gave the desired **2a-*d*** (120 mg) (deuteration ratio: 98%, determined by ^{1}H NMR).

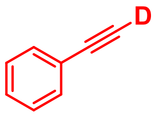

2a-*d* (CAS No. 3240-11-7)

phenylacetylene-*d* (2a-*d*): ^{1}H NMR (500 MHz, $CDCl_3$): δ 7.30–7.37 (m, 3H), 7.47–7.52 (m, 2H).

Spectral Data:

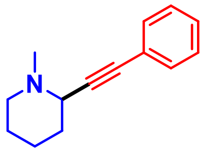

3aa (CAS No. 51498-55-6)

1-methyl-2-(phenylethynyl)piperidine (3aa): 78% isolated yield (eluent: hexane/EtOAc = 6/4). ^{1}H NMR (500 MHz, $CDCl_3$, TMS): δ 1.47–1.52 (m, 1H), 1.55–1.75 (m, 3H), 1.81–1.93 (m, 2H), 2.35–2.41 (m, 1H), 2.41 (s, 3H), 2.62–2.68 (m, 1H), 3.55 (brs, 1H), 7.27–7.32 (m, 3H) , 7.42–7.46 (m, 2H). ^{13}C–{^{1}H} NMR (125 MHz, $CDCl_3$, TMS): δ 20.9, 25.9, 31.9, 44.6, 52.1, 54.8, 86.2, 87.6, 123.5, 128.0, 128.3, 131.8. MS (70 eV, EI): *m/z* (%): 199 (44) [M^{+}], 198 (54), 184 (11), 171 (23), 170 (100), 157 (22), 156 (16), 143 (11), 142 (36), 128 (28), 127 (14), 122 (25), 116 (15), 115 (44), 102 (13), 94 (11), 79 (10). Anal. Calcd. for $C_{14}H_{17}N{\cdot}0.25H_2O$: C, 82.57; H, 8.66; N, 6.87. Found: C, 82.97; H, 8.49; N, 6.85.

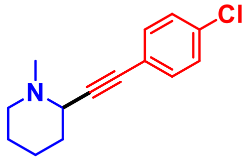

3ab

2-((4-chlorophenyl)ethynyl)-1-methylpiperidine (3ab): 77% isolated yield (eluent: hexane/EtOAc = 6/4). ^{1}H NMR (500 MHz, $CDCl_3$, TMS): δ 1.47–1.50 (m, 1H), 1.55–1.73 (m, 3H), 1.80–1.93 (m, 2H), 2.34–2.42 (m, 1H), 2.39 (s, 3H), 2.61–2.65 (m, 1H), 3.53 (brs, 1H), 7.26–7.28 (m, 2H) , 7.35–7.38 (m, 2H). ^{13}C–{^{1}H} NMR (125 MHz, $CDCl_3$, TMS): δ 21.0, 25.8, 31.9, 44.6, 52.3, 54.8, 85.0, 88.7, 122.0, 128.6, 133.0, 134.0. MS (70 eV, EI): *m/z* (%): 235 (17), 234 (26) [M^{+}], 233 (50), 232 (60), 218 (12), 206 (35), 205 (23), 204 (100), 191 (24), 190 (13), 178 (12), 177 (10), 176 (28), 162 (15), 149 (23), 127 (21), 126 (12), 122 (32), 115 (33), 113 (11), 96 (12), 94 (15). Anal. Calcd. for $C_{14}H_{16}NCl$: C, 71.94; H, 6.90; N, 5.99. Found: C, 71.54; H, 6.77; N, 5.91.

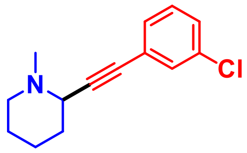

3ac

2-((3-chlorophenyl)ethynyl)-1-methylpiperidine (3ac): 77% isolated yield (eluent: hexane/EtOAc = 6/4). ^{1}H NMR (500 MHz, $CDCl_3$, TMS): δ 1.48–1.53 (m, 1H), 1.55–1.71 (m, 3H), 1.80–1.93 (m, 2H), 2.36–2.44 (m, 1H), 2.40 (s, 3H), 2.60–2.64 (m, 1H), 3.56 (brs, 1H), 7.21–7.33 (m, 3H) , 7.42–

7.43 (m, 1H). $^{13}C–\{^1H\}$ NMR (125 MHz, $CDCl_3$, TMS): δ 20.9, 25.8, 31.8, 44.6, 52.1, 54.7, 84.9, 89.0, 125.2, 128.3, 129.6, 129.9, 131.7, 134.1. MS (70 eV, EI): *m/z* (%): 235 (13), 234 (21) [M^+], 233 (41), 232 (47), 218 (11), 206 (35), 205 (24), 204 (100), 191 (20), 190 (10), 178 (11), 176 (27), 162 (13), 149 (21), 127 (20), 126 (10), 122 (36), 115 (31), 113 (10), 96 (12), 94 (18). Anal. Calcd. for $C_{14}H_{16}NCl \cdot 0.1H_2O$: C, 71.39; H, 6.93; N, 5.95. Found: C, 71.52; H, 6.71; N, 5.88.

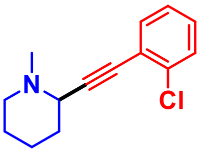

3ad

2-((2-chlorophenyl)ethynyl)-1-methylpiperidine (3ad): 87% isolated yield (eluent: hexane/EtOAc = 6/4). ^1H NMR (500 MHz, $CDCl_3$, TMS): δ 1.50–1.55 (m, 1H), 1.56–1.78 (m, 3H), 1.86–1.94 (m, 2H), 2.39–2.47 (m, 1H), 2.43 (s, 3H), 2.65–2.70 (m, 1H), 3.68 (brs, 1H), 7.18–7.24 (m, 2H), 7.38–7.40 (m, 1H), 7.47–7.48 (m, 1H). $^{13}C–\{^1H\}$ NMR (125 MHz, $CDCl_3$, TMS): δ 20.6, 25.8, 31.7, 44.5, 51.7, 54.7, 83.1, 93.1, 123.4, 126.4, 129.0, 129.3, 133.4, 136.0. MS (70 eV, EI): *m/z* (%): 235 (18), 234 (24) [M^+], 233 (54), 232 (52), 218 (12), 206 (36), 205 (24), 204 (100), 198 (24), 191 (23), 178 (12), 176 (29), 170 (11), 162 (15), 151 (11), 149 (24), 142 (10), 127 (23), 126 (12), 122 (43), 115 (36), 114 (10), 113 (12), 96 (14), 94 (16). Anal. Calcd. for $C_{14}H_{16}NCl \cdot 0.1H_2O$: C, 71.39; H, 6.93; N, 5.95. Found: C, 71.36; H, 6.55; N, 5.89.

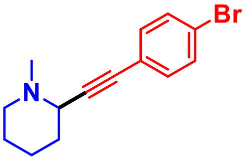

3ae

2-((4-bromophenyl)ethynyl)-1-methylpiperidine (3ae): 82% isolated yield (eluent: hexane/EtOAc = 6/4). ^1H NMR (500 MHz, $CDCl_3$, TMS): δ 1.47–1.50 (m, 1H), 1.55–1.72 (m, 3H), 1.79–1.92 (m, 2H), 2.34–2.42 (m, 1H), 2.39 (s, 3H), 2.61–2.64 (m, 1H), 3.52 (brs, 1H), 7.28–7.31 (m, 2H), 7.41–7.47 (m, 2H). $^{13}C–\{^1H\}$ NMR (125 MHz, $CDCl_3$, TMS): δ 21.0, 25.8, 31.8, 44.5, 52.2, 54.8, 85.1, 88.9, 122.1, 122.4, 131.5, 133.2. MS (70 eV, EI): *m/z* (%): 279 (53), 278 (66) [M^+], 277 (53), 276 (63), 251 (23), 250 (96), 249 (25), 248 (100), 237 (26), 235 (27), 222 (28), 220 (27), 169 (45), 127 (36), 126 (23), 122 (65), 115 (58), 114 (21), 113 (21), 94 (28). Anal. Calcd. for $C_{14}H_{16}NBr$: C, 60.45; H, 5.80; N, 5.03. Found: C, 60.09; H, 5.52; N, 5.00.

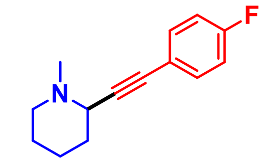

3af

2-((4-fluorophenyl)ethynyl)-1-methylpiperidine (3af): 64% isolated yield (eluent: hexane/EtOAc = 6/4). ^{1}H NMR (500 MHz, $CDCl_3$, TMS): δ 1.47–1.50 (m, 1H), 1.54–1.73 (m, 3H), 1.80–1.93 (m, 2H), 2.34–2.41 (m, 1H), 2.40 (s, 3H), 2.61–2.64 (m, 1H), 3.52 (brs, 1H), 6.97–7.01 (m, 2H), 7.39–7.43 (m, 2H). ^{13}C–{^{1}H} NMR (125 MHz, $CDCl_3$, TMS): δ 21.0, 25.8, 31.9, 44.5, 52.2, 54.8, 85.0, 87.2, 115.5 (d, J = 22.8 Hz), 119.5 (d, J = 3.6 Hz), 133.6 (d, J = 8.4 Hz), 162.3 (d, J = 248.3 Hz). ^{19}F NMR (470 MHz, $CDCl_3$): δ −112.92. MS (70 eV, EI): *m/z* (%): 217 (52) [M^+], 216 (61), 202 (12), 189 (23), 188 (100), 175 (24), 174 (16), 161 (12), 160 (36), 146 (30), 145 (12), 134 (15), 133 (50), 122 (22), 120 (15), 94 (15), 88 (12). Anal. Calcd. for $C_{14}H_{16}NF\cdot0.35H_2O$: C, 75.21; H, 7.53; N, 6.26. Found: C, 75.08; H, 7.09; N, 6.21.

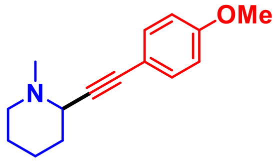

3ag

1-methyl-2-(phenylethynyl)piperidine (3ag): 76% isolated yield (eluent: hexane/EtOAc = 8/2). ^{1}H NMR (500 MHz, $CDCl_3$, TMS): δ 1.46–1.50 (m, 1H), 1.54–1.76 (m, 3H), 1.79–1.89 (m, 2H), 2.33–2.41 (m, 1H), 2.40 (s, 3H), 2.63–2.67 (m, 1H), 3.51 (brs, 1H), 3.80 (s, 3H), 6.81–6.84 (m, 2H), 7.36–7.39 (m, 2H). ^{13}C–{^{1}H} NMR (125 MHz, $CDCl_3$, TMS): δ 21.1, 25.9, 32.1, 44.6, 52.3, 54.9, 55.4, 85.9, 86.1, 113.9, 115.7, 133.2, 159.4. MS (70 eV, EI): *m/z* (%): 230 (14), 229 (87) [M^+], 228 (100), 214 (32), 201 (21), 200 (88), 187 (17), 186 (29), 172 (55), 159 (14), 158 (51), 157 (11), 145 (21), 144 (12), 143(12), 130 (14), 128 (10), 122 (31), 115 (23), 94 (14), 89 (11). Anal. Calcd. for $C_{15}H_{19}NO$: C, 78.56; H, 8.35; N, 6.11. Found: C, 77.92; H, 8.38; N, 5.95.

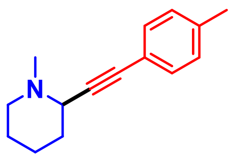

3ah

1-methyl-2-(*p*-tolylethynyl)piperidine (3ah): 82% isolated yield (eluent: hexane/EtOAc = 6/4). ^{1}H NMR (500 MHz, $CDCl_3$, TMS): δ 1.46–1.49 (m, 1H), 1.54–1.74 (m, 3H), 1.80–1.92 (m, 2H), 2.34 (s, 3H), 2.35–2.43 (m, 1H), 2.40 (s, 3H), 2.63–2.67 (m, 1H), 3.54 (brs, 1H), 7.10–7.11 (m, 2H), 7.33–

7.34 (m, 2H). ^{13}C–{^{1}H} NMR (125 MHz, $CDCl_3$, TMS): δ 21.0, 21.5, 25.8, 31.9, 44.5, 52.1, 54.8, 86.2, 86.7, 120.4, 129.1, 131.7, 138.0. MS (70 eV, EI): *m/z* (%): 213 (64) [M^+], 212 (71), 198 (19), 185 (25), 184 (100), 171 (24), 170 (25), 157 (12), 156 (57), 142 (20), 141 (22), 129 (28), 128 (20), 122 (30), 115 (30), 94 (11), 86 (14). Anal. Calcd. for $C_{15}H_{19}N{\cdot}0.15H_2O$: C, 83.40; H, 9.01; N, 6.48. Found: C, 83.29; H, 8.79; N, 6.38.

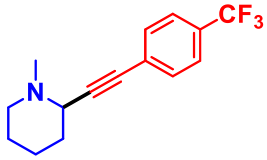

3ai

1-methyl-2-((4-(trifluoromethyl)phenyl)ethynyl)piperidine (3ai): 72% isolated yield (eluent: hexane/EtOAc = 6/4). ^{1}H NMR (500 MHz, $CDCl_3$, TMS): δ 1.49–1.52 (m, 1H), 1.56–1.73 (m, 3H), 1.82–1.95 (m, 2H), 2.34–2.42 (m, 1H), 2.41 (s, 3H), 2.62–2.65 (m, 1H), 3.56 (brs, 1H), 7.52–7.57 (m, 4H). ^{13}C–{^{1}H} NMR (125 MHz, $CDCl_3$, TMS): δ 21.0, 25.8, 31.8, 44.6, 52.2, 54.8, 84.9, 90.5, 124.1 (q, J = 272.3 Hz), 125.2–125.3 (m), 127.3, 129.8 (q, J = 129.8 Hz), 132.0. ^{19}F NMR (470 MHz, $CDCl_3$): δ −65.4. MS (70 eV, EI): *m/z* (%): 267 (39) [M^+], 266 (47), 252 (11), 239 (26), 238 (100), 225 (20), 224 (11), 210 (27), 183 (17), 127 (11), 122 (17), 115 (11), 94 (11). Anal. Calcd. for $C_{15}H_{16}F_3N$: C, 67.40; H, 6.03; N, 5.24. Found: C, 67.11; H, 5.80; N, 5.15.

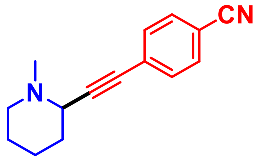

3aj

4-((1-methylpiperidin-2-yl)ethynyl)benzonitrile (3aj): 66% isolated yield (eluent: hexane/EtOAc = 6/4). ^{1}H NMR (500 MHz, $CDCl_3$, TMS): δ 1.49–1.54 (m, 1H), 1.56–1.71 (m, 3H), 1.81–1.94 (m, 2H), 2.36–2.44 (m, 1H), 2.40 (s, 3H), 2.60–2.64 (m, 1H), 3.57 (brs, 1H), 7.51–7.53 (m, 2H), 7.59–7.61 (m, 2H). ^{13}C–{^{1}H} NMR (125 MHz, $CDCl_3$, TMS): δ 20.9, 25.7, 31.6, 44.5, 52.1, 54.8, 84.7, 92.7, 111.3, 118.6, 128.4, 132.0, 132.3. MS (70 eV, EI): *m/z* (%): 224 (37) [M^+], 223 (44), 209 (11), 196 (23), 195 (100), 182 (20), 181 (13), 167 (29), 153 (18), 141 (13), 140 (34), 127 (15), 122 (20), 94 (12). Anal. Calcd. for $C_{15}H_{16}N_2{\cdot}0.2H_2O$: C, 79.05; H, 7.25; N, 12.29. Found: C, 79.18; H, 7.10; N, 12.25.

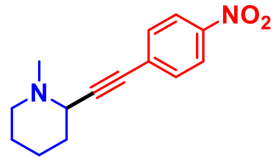

3ak

1-methyl-2-((4-nitrophenyl)ethynyl)piperidine (3ak): 61% isolated yield (eluent: hexane/EtOAc = 6/4). ^{1}H NMR (500 MHz, $CDCl_3$, TMS): δ 1.51–1.55 (m, 1H), 1.57–1.72 (m, 3H), 1.82–1.96 (m, 2H), 2.37–2.45 (m, 1H), 2.41 (s, 3H), 2.61–2.65 (m, 1H), 3.59 (brs, 1H), 7.57–7.59 (m, 2H), 8.17–8.19 (m, 2H). ^{13}C–{^{1}H} NMR (125 MHz, $CDCl_3$, TMS): δ 20.9, 25.7, 31.6, 44.6, 52.1, 54.9, 84.6, 93.8, 123.6, 130.4, 132.5, 146.9. MS (70 eV, EI): *m/z* (%): 244 (48) [M^+], 243 (49), 229 (13), 216 (27), 215 (100), 202 (13), 197 (20), 187 (23), 169 (29), 141 (21), 128 (11), 127 (11), 122 (25), 115 (30), 114 (10), 94 (16). Anal. Calcd. for $C_{14}H_{16}N_2O_2{\cdot}0.1H_2O$: C, 68.33; H, 6.64; N, 11.38. Found: C, 68.18; H, 6.45; N, 11.32.

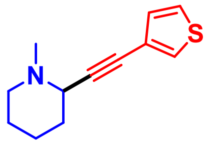

3al

1-methyl-2-(thiophen-3-ylethynyl)piperidine (3al): 66% isolated yield (eluent: hexane/EtOAc = 6/4). ^{1}H NMR (500 MHz, $CDCl_3$, TMS): δ 1.46–1.49 (m, 1H), 1.55–1.73 (m, 3H), 1.79–1.92 (m, 2H), 2.39 (s, 3H), 2.32–2.40 (m, 1H), 2.62–2.65 (m, 1H), 3.52 (brs, 1H), 7.11 (dd, J = 5.2 and 1.2 Hz, 1H), 7.25 (dd, J = 5.2 and 2.9 Hz, 1H), 7.40 (dd, J = 2.9 and 1.2 Hz, 1H). ^{13}C–{^{1}H} NMR (125 MHz, $CDCl_3$, TMS): δ 21.0, 25.8, 31.9, 44.6, 52.2, 54.9, 81.1, 87.2, 122.4, 125.2, 128.2, 130.2. MS (70 eV, EI): *m/z* (%): 206 (11), 205 (59) [M^+], 204 (69), 190 (12), 177 (30), 176 (100), 163 (26), 162 (22), 149 (13), 148 (53), 135 (12), 134 (27), 122 (26), 121 (34), 108 (11), 94 (13), 82 (15). Anal. Calcd. for $C_{12}H_{15}NS{\cdot}0.1H_2O$: C, 69.59; H, 7.40; N, 6.76. Found: C, 69.32; H, 7.13; N, 6.64.

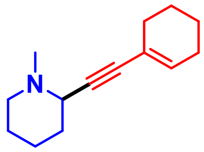

3am

2-(cyclohex-1-en-1-ylethynyl)-1-methylpiperidine (3am): 74% isolated yield (eluent: hexane/EtOAc = 6/4). ^{1}H NMR (500 MHz, $CDCl_3$, TMS): δ 1.42–1.46 (m, 1H), 1.51–1.68 (m, 7H), 1.71–1.86 (m, 2H), 2.06–2.15 (m, 4H), 2.29–2.37 (m ,1H), 2.33 (s, 3H), 2.56–2.59 (m, 1H), 3.43 (brs, 1H), 6.06–6.09 (m, 1H). ^{13}C–{^{1}H} NMR (125 MHz, $CDCl_3$, TMS): δ 20.9, 21.6, 22.4, 25.6, 25.8, 29.7,

32.0, 44.4, 52.0, 54.7, 84.5, 87.9, 120.7, 134.0. MS (70 eV, EI): *m/z* (%): 204 (14), 203 (100) [M^+], 202 (93), 188 (32), 175 (21), 174 (76), 161 (23), 160 (54), 148 (11), 147 (22), 146 (97), 134 (17), 133 (13), 132 (37), 131 (21), 122 (53), 120 (18), 119 (14), 118 (31), 117 (32), 115 (19), 105 (16), 104 (10), 103 (12), 98 (12), 96 (13), 94 (24), 91 (46), 82 (12), 81 (16), 80 (15), 79 (23), 78 (14), 77 (27), 70 (19), 68 (11), 67 (11), 65 (18), 55 (11), 51 (12). Anal. Calcd. for $C_{14}H_{21}N\cdot0.25H_2O$: C, 80.91; H, 10.43; N, 6.74. Found: C, 80.96; H, 10.11; N, 6.66.

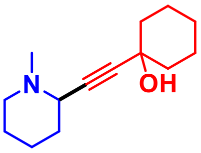

3an

1-((1-methylpiperidin-2-yl)ethynyl)cyclohexan-1-ol (3an): 30% isolated yield (eluent: EtOAc). ^{1}H NMR (500 MHz, $CDCl_3$, TMS): δ 1.18–1.28 (m, 1H), 1.42–1.48 (m, 1H), 1.52–1.95 (m, 14H), 2.32 (s, 3H), 2.31–2.39 (m, 1H), 2.54–2.57 (m, 1H), 2.85 (brs, 1H), 3.39 (brs, 1H). ^{13}C–{^{1}H} NMR (125 MHz, $CDCl_3$, TMS): δ 20.8, 23.7, 25.3, 25.6, 31.8, 40.4, 44.4, 51.8, 54.1, 68.8, 81.7, 90.0. MS (70 eV, EI): *m/z* (%): 221 (20) [M^+], 220 (22), 178 (14), 150 (13), 125 (10), 124 (100) ,122 (52), 108 (14), 98 (21), 96 (21), 95 (12), 94 (30), 82 (12), 81 (13), 80 (12), 79 (11), 70 (21), 66 (15), 55 (20). Anal. Calcd. for $C_{14}H_{23}NO\cdot0.5H_2O$: C, 73.00; H, 10.50; N, 6.08. Found: C, 72.75; H, 10.08; N, 6.04.

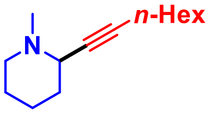

3ao

1-methyl-2-(oct-1-yn-1-yl)piperidine (3ao): 26% isolated yield (eluent: hexane/EtOAc = 6/4). ^{1}H NMR (500 MHz, $CDCl_3$, TMS): δ 0.89 (t, J = 7.1 Hz, 3H), 1.27–1.35 (m, 4H), 1.38–1.44 (m, 3H), 1.48–1.72 (m, 6H), 1.77–1.83 (m, 1H), 2.22 (td, J = 7.0 and 1.9 Hz, 2H), 2.32 (s, 3H), 2.28–2.36 (m, 1H), 2.57–2.60 (m, 1H), 2.92 (brs, 1H). ^{13}C–{^{1}H} NMR (125 MHz, $CDCl_3$, TMS): δ 14.2, 18.8, 21.0, 22.7, 25.9, 28.6, 29.2, 31.4, 32.4, 44.4, 52.2, 54.5, 77.9, 86.0. MS (70 eV, EI): *m/z* (%): 207 (26) [M^+], 206 (48), 192 (19), 178 (21), 164 (19), 151 (12), 150 (100), 137 (69), 136 (49), 134 (14), 124 (18), 123 (12), 122 (84), 120 (10), 110 (14), 109 (24), 108 (57), 107 (13), 98 (24), 96 (38), 95 (22), 94 (62), 93 (16), 91 (18), 84 (11), 82 (27), 81 (20), 80 (19), 79 (26), 77 (19), 70 (25), 68 (13), 67 (20), 65 (14), 57 (14), 55 (23), 53 (15). Anal. Calcd. for $C_{14}H_{25}N\cdot0.25H_2O$: C, 79.37; H, 12.13; N, 6.61. Found: C, 79.28; H, 11.92; N, 6.34.

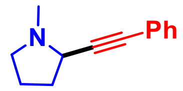

3ba (CAS No. 87143-80-4)

1-methyl-2-(phenylethynyl)pyrrolidine: 49% isolated yield (eluent: hexane/EtOAc = 8/2). ^{1}H NMR (500 MHz, $CDCl_3$, TMS): δ 1.78–1.85 (m, 1H), 1.91–2.06 (m, 2H), 2.17–2.23 (m, 1H), 2.38–2.43 (m, 1H), 2.49 (s, 3H), 2.94 (td, J = 8.8 and 4.0 Hz, 1H), 3.32 (t, J = 7.1 Hz, 1H), 7.27–7.30 (m, 3H), 7.41–7.45 (m, 2H). ^{13}C–{^{1}H} NMR (125 MHz, $CDCl_3$, TMS): δ 22.6, 32.4, 40.1, 55.0, 57.2, 84.3, 89.0, 123.4, 128.0 128.3, 131.8. MS (70 eV, EI): *m/z* (%): 186 (6), 185 (47) [M^{+}], 184 (100), 170 (5), 158 (6), 157 (45), 156 (36), 143 (8), 142 (24), 141 (8), 129 (13), 128 (16), 127 (10), 116 (14), 115 (42), 114 (12), 113 (6), 108 (37), 102 (6), 89 (5), 84 (6), 82 (6), 77 (8), 63 (8), 55 (8), 51 (5).

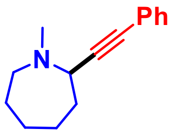

3ca (CAS No. 125038-93-9)

1-methyl-2-(phenylethynyl)azepane: 48% isolated yield (eluent: hexane/EtOAc = 8/2). ^{1}H NMR (500 MHz, $CDCl_3$, TMS): δ 1.63–1.72 (m, 5H), 1.82–2.06 (m, 3H), 2.49 (s, 3H), 2.58–2.63 (m, 1H), 2.76–2.81 (m, 1H), 3.82 (dd, J = 6.7 and 4.2 Hz, 1H), 7.27–7.32 (m, 3H), 7.42–7.47 (m, 2H). ^{13}C–{^{1}H} NMR (125 MHz, $CDCl_3$, TMS): δ 23.8, 26.9, 28.0, 34.3, 45.3, 53.6, 57.2, 86.0, 88.3, 123.7, 127.9, 128.4, 131.8. MS (70 eV, EI): *m/z* (%): 213 (49) [M^{+}], 212 (19), 198 (12), 184 (33), 171 (21), 170 (100), 157 (30), 156 (19), 142 (23), 141 (14), 129 (13), 128 (19), 127 (11), 116 (13), 115 (53), 114 (11), 102 (11), 94 (19).

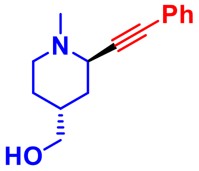

3da

(*trans*-1-methyl-2-(phenylethynyl)piperidin-4-yl)methanol: 55% isolated yield (eluent: EtOAc). ^{1}H NMR (500 MHz, $CDCl_3$, TMS): δ 1.30 (qd, J = 12.0 and 5.0 Hz, 1H, $NCH_2CH_aH_e$), 1.56 (td, J = 13.1 and 4.4 Hz, 1H, $NCHCH_aH_e$), 1.72–1.76 (m, 1H, $NCH_2CH_aH_e$), 1.92–2.01 (m, 2H, $NCHCH_aH_e$, OCH_2CH), 2.38 (s, 3H, CH_3), 2.57 (td, J = 11.9 and 2.9 Hz, 1H, NCH_aH_e), 2.60–2.70 (m, 2H, NCH_aH_e, OH), 3.49 (d, J = 6.7 Hz, 2H, OCH_2), 3.93 (dd, J = 4.2 and 2.7 Hz, 1H, NCH), 7.28–7.32 (m, 3H), 7.41–7.46 (m, 2H). ^{13}C–{^{1}H} NMR (125 MHz, $CDCl_3$, TMS): δ 28.8, 33.7, 34.0, 43.9, 49.4, 53.3,

67.6, 86.0, 87.4, 123.3, 128.1, 128.3, 131.8. MS (70 eV, EI): *m/z* (%): 229 (34) [M^+], 228 (65), 198 (45), 171 (27), 170 (100), 157 (38), 156 (26), 155 (31), 153 (29), 152 (46), 142 (50), 129 (26), 128 (54), 127 (31), 116 (23), 115 (73), 102 (22), 96 (36), 94 (21), 77 (24). Anal. Calcd. for $C_{15}H_{19}NO\cdot0.5H_2O$: C, 75.59; H, 8.46; N, 5.88. Found: C, 75.72; H, 8.23; N, 5.80.

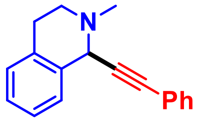

3ea (CAS No. 1356845-31-2)

2-methyl-1-(phenylethynyl)-1,2,3,4-tetrahydroisoquinoline: 29% isolated yield (eluent: hexane/EtOAc = 8/2). ^{1}H NMR (500 MHz, $CDCl_3$, TMS): δ 2.63 (s, 3H), 2.68–2.74 (m, 1H), 2.88–3.12 (m, 3H), 4.70 (s, 1H), 7.11–7.14 (m, 1H), 7.16–7.20 (m, 2H), 7.26–7.29 (m, 3H), 7.34–7.38 (m, 1H), 7.40–7.44 (m, 2H). ^{13}C–{^{1}H} NMR (125 MHz, $CDCl_3$, TMS): δ 28.9, 43.9, 48.8, 57.1, 86.4, 87.6, 123.3, 126.0, 127.1, 127.8, 128.2, 128.3, 129.0, 131.9, 133.6, 135.3. MS (70 eV, EI): *m/z* (%): 248 (9), 247 (57) [M^+], 246 (100), 205 (6), 204 (34), 203 (30), 202 (31), 170 (21), 146 (5), 144 (10), 115 (11), 103 (5), 101 (7), 77 (6).

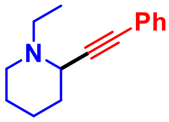

3fa

1-ethyl-2-(phenylethynyl)piperidine: 65% isolated yield (eluent: hexane/EtOAc = 8/2). ^{1}H NMR (500 MHz, $CDCl_3$, TMS): δ 1.11 (t, J = 7.2 Hz, 3H), 1.53–1.76 (m, 4H), 1.83–1.89 (m, 2H), 2.52–2.66 (m, 4H), 3.85 (brs, 1H), 7.28–7.32 (m, 3H), 7.42–7.46 (m, 2H). ^{13}C–{^{1}H} NMR (125 MHz, $CDCl_3$, TMS): δ 12.3, 21.1, 25.9, 31.6, 49.1, 50.1, 51.7, 86.4, 87.3, 123.6, 127.19, 128.3, 131.8. MS (70 eV, EI): *m/z* (%): 213 (50) [M^+], 212 (36), 198 (61), 185 (24), 184 (100), 170 (10), 156 (39), 142 (10), 141 (12), 136 (12), 129 (16), 128 (49), 127 (13), 116 (11), 115 (58), 108 (10), 102 (12), 77 (11), 70 (12), 56 (22). Anal. Calcd. for $C_{15}H_{19}N\cdot0.1H_2O$: C, 83.75; H, 9.00; N, 6.51. Found: C, 83.58; H, 8.91; N, 6.41.

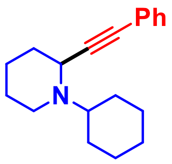

3ga

1-cyclohexyl-2-(phenylethynyl)piperidine: 63% isolated yield (eluent: hexane/EtOAc = 9/1). ^{1}H NMR (500 MHz, $CDCl_3$, TMS): δ 1.09–1.32 (m, 5H), 1.48–1.90 (m, 9H), 1.99–2.08 (m, 2H), 2.50–2.62 (m, 2H), 2.72–2.76 (m, 1H), 4.02 (t, J = 4.2 Hz, 1H), 7.27–7.32 (m, 3H), 7.40–7.45 (m, 2H). ^{13}C–{^{1}H} NMR (125 MHz, $CDCl_3$, TMS): δ 21.5, 26.0, 26.3, 28.9, 31.1, 32.4, 45.3, 49.5, 61.3, 86.1, 88.8, 123.8, 127.8, 128.3, 131.7. MS (70 eV, EI): *m/z* (%): 267 (28) [M^{+}], 266 (12), 238 (18), 224 (25), 211 (37), 210 (41), 190 (18), 184 (17), 156 (12), 141 (13), 135 (11), 134 (100), 128 (27), 115 (20), 55 (18). Anal. Calcd. for $C_{19}H_{25}N \cdot 0.1H_2O$: C, 84.77; H, 9.43; N, 5.20. Found: C, 84.86; H, 9.40; N, 5.14.

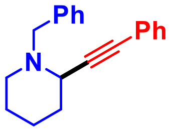

3ha (CAS No. 479543-27-6)

benzyl-2-(phenylethynyl)piperidine: 72% isolated yield (eluent: hexane/EtOAc = 9/1). ^{1}H NMR (500 MHz, $CDCl_3$, TMS): δ 1.52–1.64 (m, 3H), 1.69–1.77 (m, 1H), 1.80–1.86 (m, 2H), 2.51–2.54 (m, 1H), 2.61–2.64 (m, 1H), 3.61–3.80 (m, 3H), 7.23–7.26 (m, 1H), 7.29–7.35 (m, 5H), 7.38–7.41 (m, 2H), 7.47–7.51 (m, 2H).^{13}C–{^{1}H} NMR (125 MHz, $CDCl_3$, TMS): δ 20.8, 25.8, 31.4, 49.3, 51.7, 60.6, 86.7, 87.5, 123.6, 126.9, 127.9, 128.2, 128.3, 129.3, 131.8, 138.6. MS (70 eV, EI): *m/z* (%): 276 (12), 275 (56) [M^{+}], 274 (39), 248 (10), 247 (13), 246 (31), 198 (21), 185 (11), 184 (73), 156 (16), 142 (12), 129 (19), 128 (49), 115 (38), 91 (100), 65 (18).

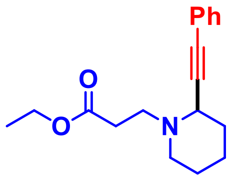

3ia

ethyl 3-(2-(phenylethynyl)piperidin-1-yl)propanoate: 50% isolated yield (eluent: hexane/EtOAc = 8/2). ^{1}H NMR (500 MHz, $CDCl_3$, TMS): δ 1.26 (t, J = 7.2 Hz, 3H), 1.51–1.75 (m, 4H), 1.79–1.89 (m, 2H), 2.50–2.63 (m, 4H), 2.85–2.89 (m, 2H), 3.81–3.85 (m, 1H), 4.14 (q, J = 7.2 Hz, 2H), 7.28–7.32 (m, 3H), 7.42–7.46 (m, 2H). ^{13}C–{^{1}H} NMR (125 MHz, $CDCl_3$, TMS): δ 14.4, 20.8, 25.9, 31.6, 32.9, 49.1, 51.8, 52.3, 60.4, 86.6, 87.1, 123.5, 128.0, 128.3, 131.8, 172.8. MS (70 eV, EI): *m/z* (%):285 (3)

[M^+], 256 (11), 240 (12), 212 (52), 199 (10), 198 (69), 197 (22), 196 (10), 185 (15), 184 (100), 156 (15), 141 (11), 129 (12), 128 (31), 127 (10), 115 (32), 91 (11), 70 (13). Anal. Calcd. for $C_{18}H_{23}NO_2$: C, 75.76; H, 8.12; N, 4.91. Found: C, 75.50; H, 8.02; N, 4.82.

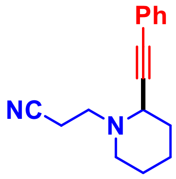

3ja

3-(2-(phenylethynyl)piperidin-1-yl)propanenitrile: 48% isolated yield (eluent: hexane/EtOAc = 8/2). ^{1}H NMR (500 MHz, $CDCl_3$, TMS): δ 1.52–1.75 (m, 4H), 1.83–1.90 (m, 2H), 2.54 (t, J = 7.2 Hz, 2H), 2.57 (t, J = 4.0 Hz, 1H), 2.63–2.68 (m, 1H), 2.79–2.91 (m, 2H), 3.81 (t, J = 4.0 Hz, 1H), 7.28–7.33 (m, 3H), 7.41–7.45 (m, 2H). ^{13}C–{^{1}H} NMR (125 MHz, $CDCl_3$, TMS): δ 16.4, 20.6, 25.7, 31.5, 49.1, 51.7, 52.4, 86.5, 86.8, 119.1, 123.1, 128.3, 128.4, 131.8. MS (70 eV, EI): m/z (%): 238 (4) [M^+], 237 (6), 209 (6), 199 (17), 198 (100), 184 (7), 170 (5), 156 (12), 155 (10), 142 (8), 141 (10), 129 (9), 128 (26), 127 (9), 116 (5), 115 (37), 102 (8), 91 (9), 77 (7), 70 (19), 55 (6), 54 (9). Anal. Calcd. for $C_{16}H_{18}N_2$: C, 80.63; H, 7.61; N, 11.75. Found: C, 80.35; H, 7.58; N, 11.67.

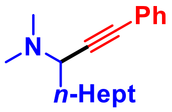

3ka

***N*,*N*-dimethyl-1-phenyldec-1-yn-3-amine**: 53% isolated yield (eluent: hexane/EtOAc = 8/2). ^{1}H NMR (500 MHz, $CDCl_3$, TMS): δ 0.88 (t, J = 7.2 Hz, 3H), 1.28–1.74 (m, 12H), 2.32 (s, 6H), 3.51 (dd, J = 8.5 and 6.5 Hz, 1H), 7.27–7.32 (m, 3H), 7.42–7.46 (m, 2H). ^{13}C–{^{1}H} NMR (125 MHz, $CDCl_3$, TMS): δ 14.2, 22.8, 26.9, 29.4, 29.5, 32.0, 34.1, 41.6, 58.4, 86.1, 87.2, 123.6, 128.0, 128.4, 131.9. MS (70 eV, EI): m/z (%): 257 (0.1) [M^+], 159 (13), 158 (100), 129 (1), 128 (3), 127 (1), 116 (2), 115 (22), 102 (1), 91 (2), 89 (1), 84 (2), 55 (1). Anal. Calcd. for $C_{18}H_{27}N\cdot 0.25H_2O$: C, 82.54; H, 10.58; N, 5.35. Found: C, 82.28; H, 10.37; N, 5.16.

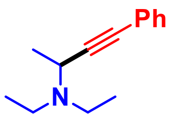

3la (CAS No. 153396-40-8)

***N*,*N*-diethyl-4-phenylbut-3-yn-2-amine**: 27% isolated yield (eluent: hexane/EtOAc = 9/1). ^{1}H NMR (500 MHz, $CDCl_3$, TMS): δ 1.11 (t, J = 7.1 Hz, 6H), 1.42 (d, J = 7.1 Hz, 3H), 2.48–2.56 (m, 2H), 2.71–2.78 (m, 2H), 3.90 (q, J = 7.1 Hz, 1H), 7.27–7.31 (m, 3H), 7.39–7.43 (m, 2H). ^{13}C–{^{1}H} NMR (125 MHz, $CDCl_3$, TMS): δ 13.8, 20.2, 44.8, 48.4, 84.2, 89.6, 123.6, 127.9, 128.3, 131.8. MS (70 eV, EI): *m/z* (%): 201 (3) [M^+], 187 (15), 186 (100), 130 (6), 129 (36), 128 (25), 127 (11), 115 (18), 103 (5), 77 (7), 58 (6), 56 (7).

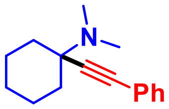

3ma (CAS No. 1313814-49-1)

***N*,*N*-dimethyl-1-(phenylethynyl)cyclohexan-1-amine**: 64% isolated yield (eluent: hexane/EtOAc = 4/6). ^{1}H NMR (500 MHz, $CDCl_3$, TMS): δ 1.19–1.27 (m, 1H), 1.44–1.49 (m, 2H), 1.59–1.75 (m, 5H), 2.05–2.11 (m, 2H), 2.36 (s, 6H), 7.27–7.32 (m, 3H), 7.42–7.47 (m, 2H). ^{13}C–{^{1}H} NMR (125 MHz, $CDCl_3$, TMS): δ 23.2, 25.7, 36.5, 39.5, 59.9, 86.5, 89.5, 123.8, 127.9, 128.3, 131.9. MS (70 eV, EI): *m/z* (%): 227 (20) [M^+], 212 (31), 198 (11), 185 (16), 184 (100), 170 (17), 150 (13), 142 (10), 141 (30), 128 (11), 115 (24). Anal. Calcd. for $C_{16}H_{21}N\cdot 0.1H_2O$: C, 83.86; H, 9.32; N, 6.11. Found: C, 83.86; H, 8.88; N, 5.65.

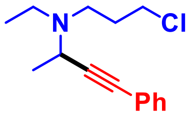

3na

***N*-(3-chloropropyl)-*N*-ethyl-4-phenylbut-3-yn-2-amine**: 28% isolated yield (eluent: hexane/EtOAc = 9/1). ^{1}H NMR (500 MHz, $CDCl_3$, TMS): δ 1.10 (t, J = 7.2 Hz, 3H), 1.40 (d, J = 7.1 Hz, 3H), 1.87–2.00 (m, 2H), 2.49–2.60 (m, 2H), 2.64–2.71 (m, 1H), 2.76–2.84 (m, 1H), 3.59–3.68 (m, 2H), 3.90 (q, J = 7.1 Hz, 1H), 7.27–7.31 (m, 3H), 7.39–7.44 (m, 2H). ^{13}C–{^{1}H} NMR (125 MHz, $CDCl_3$, TMS): δ 14.0, 20.5, 31.7, 43.5, 45.7, 47.6, 48.7, 84.2, 89.5, 123.6, 128.0, 128.4, 131.8. MS (70 eV, EI): *m/z* (%): 249 (3) [M^+], 237 (5), 236 (34), 235 (17), 234 (100), 186 (17), 130 (11), 129 (78), 128 (36), 127

(15), 115 (16), 103 (5), 77 (8), 58 (11), 56 (5). Anal. Calcd. for $C_{15}H_{20}ClN$: C, 72.13; H, 8.07; N, 5.61. Found: C, 72.62; H, 7.82; N, 5.33.

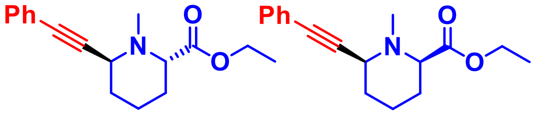

3oa

***trans*-ethyl 1-methyl-6-(phenylethynyl)piperidine-2-carboxylate, *cis*-ethyl 1-methyl-6-(phenylethynyl)piperidine-2-carboxylate**: *trans*-isomer: 39% isolated yield (eluent: hexane/EtOAc = 6/4). ^{1}H NMR (500 MHz, $CDCl_3$, TMS): δ 1.29 (t, J = 7.1 Hz, 3H), 1.60–1.70 (m, 2H), 1.77–1.91 (m, 3H), 1.94–2.01 (m, 1H), 2.40 (s, 3H), 3.26 (dd, J = 10.6 and 3.0 Hz, 1H), 4.01 (t, J = 3.6 Hz, 1H), 4.22 (q, J = 7.1 Hz, 2H), 7.29–7.33 (m, 3H), 7.43–7.47 (m, 2H). ^{13}C–{^{1}H} NMR (125 MHz, $CDCl_3$, TMS): δ 14.4, 19.3, 30.0, 30.7, 41.8, 53.8, 60.9, 63.1, 86.1, 87.5, 123.2, 128.2, 128.4, 131.9, 173.8. MS (70 eV, EI): *m/z* (%): 271 (2) [M^+], 199 (15), 198 (100), 170 (6), 169 (6), 167 (10), 142 (8), 141 (38), 128 (8), 115 (19), 102 (5), 96 (45), 91 (6), 85 (9). Anal. Calcd. for $C_{17}H_{21}NO_2$: C, 75.25; H, 7.80; N, 5.16. Found: C, 75.52; H, 7.45; N, 4.93. *cis*-isomer: 5% isolated yield (eluent: hexane/EtOAc = 6/4). ^{1}H NMR (500 MHz, $CDCl_3$, TMS): δ 1.29 (t, J = 7.1 Hz, 3H), 1.35–1.47 (m, 1H), 1.70–1.80 (m, 1H), 1.84–1.94 (m, 3H), 2.00–2.08 (m, 1H), 2.50 (s, 3H), 2.77 (dd, J = 11.5 and 2.7 Hz, 1H), 3.02 (dd, J = 11.1 and 3.1 Hz, 1H), 4.24 (qd, J = 7.1 and 1.0 Hz, 2H), 7.27–7.30 (m, 3H), 7.39–7.42 (m, 2H). ^{13}C–{^{1}H} NMR (125 MHz, $CDCl_3$, TMS): δ 14.4, 23.5, 29.8, 32.7, 42.5, 56.9, 61.0, 69.0, 84.4, 89.4, 123.4, 128.2, 128.3, 131.7, 173.2. MS (70 eV, EI): *m/z* (%): 271 (1) [M^+], 199 (16), 198 (100), 167 (9), 142 (7), 141 (36), 128 (7), 115 (17), 96 (35), 85 (6).

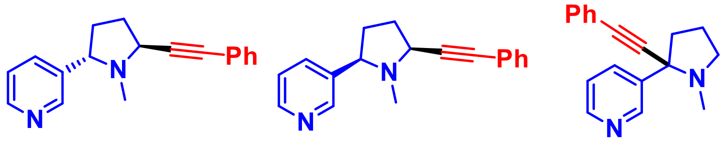

3pa

***trans*-3-(1-methyl-5-(phenylethynyl)pyrrolidin-2-yl)pyridine, *cis*-3-(1-methyl-5-(phenylethynyl)pyrrolidin-2-yl)pyridine, 3-(1-methyl-2-(phenylethynyl)pyrrolidin-2-yl)pyridine**: 32% isolated yield in total (eluent: hexane/EtOAc = 4/6) (the ratio determined by ^{1}H NMR: *trans*-isomer/*cis*-isomer/*regio*-isomer = 77/18/5). Anal. Calcd. for $C_{18}H_{18}N_2 \cdot 0.1H_2O$: C, 81.84; H, 6.94; N, 10.60. Found: C, 81.57; H, 6.80; N, 10.50. *trans*-isomer: ^{1}H NMR (500 MHz, $CDCl_3$, TMS): δ 1.71–1.79 (m, 1H), 2.07 (dddd, J = 12.3, 9.2, 3.4 and 1.2 Hz, 1H), 2.29 (s, 3H), 2.31–2.39

(m, 1H), 2.43–2.53 (m, 1H), 3.63 (dd, J = 8.8 and 7.1 Hz, 1H), 4.27 (d, J = 6.5 Hz, 1H), 7.25–7.36 (m, 4H), 7.44–7.49 (m, 2H), 7.70 (dt, J = 7.8 and 1.8 Hz, 1H), 8.51 (dd, J = 4.8 and 1.7 Hz, 1H), 8.57 (d, J = 1.9 Hz, 1H). ^{13}C–{^{1}H} NMR (125 MHz, $CDCl_3$, TMS): δ 31.0, 33.9, 36.5, 57.0, 64.9, 86.5, 87.5, 123.3, 123.7, 128.1, 128.4, 131.9, 135.1, 139.3, 148.8, 149.7. MS (70 eV, EI): *m/z* (%): 263 (10), 262 (60) [M^+], 261 (100), 234 (27), 233 (42), 219 (12), 193 (12), 185 (27), 184 (37), 156 (13), 155 (23), 142 (26), 129 (32), 128 (16), 119 (10), 118 (18), 115 (21). *cis*-isomer: ^{1}H NMR (500 MHz, $CDCl_3$, TMS): δ 1.77–1.87 (m, 1H), 2.10–2.29 (m, 3H), 2.30 (s, 3H), 3.22–3.27 (m, 1H), 3.31–3.37 (m, 1H), 7.26–7.34 (m, 4H), 7.45–7.49 (m, 2H), 7.79 (dt, J = 7.8 and 2.0 Hz, 1H), 8.52 (dd, J = 4.8 and 1.7 Hz, 1H), 8.56 (d, J = 1.9 Hz, 1H). ^{13}C–{^{1}H} NMR (125 MHz, $CDCl_3$, TMS): δ 30.9, 33.4, 39.2, 58.7, 68.7, 83.8, 89.6, 123.3, 123.8, 128.2, 128.4, 131.9, 135.2, 138.4, 149.1, 149.7. MS (70 eV, EI): *m/z* (%): 263 (11), 262 (62) [M^+], 261 (100), 234 (24), 233 (37), 219 (11), 193 (11), 185 (30), 184 (52), 156 (16), 155 (34), 143 (10), 142 (22), 129 (36), 128 (18), 127 (10). 118 (22), 115 (24). *regio*-isomer: ^{1}H NMR (500 MHz, $CDCl_3$, TMS): δ 1.96–2.53 (m, 4H), 2.20 (s, 3H), 2.72–2.78 (m, 1H), 3.22–3.27 (m, 1H), 7.25–7.36 (m, 4H), 7.44–7.54 (m, 2H), 8.02 (dt, J = 8.0 and 2.0 Hz, 1H), 8.49–8.59 (m, 1H), 9.00 (d, J = 1.7 Hz, 1H). ^{13}C–{^{1}H} NMR (125 MHz, $CDCl_3$, TMS): δ 21.7, 36.1, 44.8, 53.8, 67.3, 86.6, 89.1, 123.1, 123.6, 128.2, 128.3, 131.9, 134.6, 138.6, 148.7, 149.2. MS (70 eV, EI): *m/z* (%): 262 (12) [M^+], 261 (9), 233 (10), 185 (18), 184 (100), 115 (6).

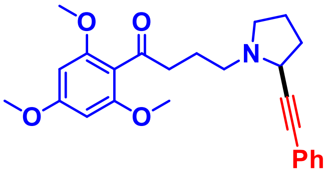

3qa

4-(2-(phenylethynyl)pyrrolidin-1-yl)-1-(2,4,6-trimethoxyphenyl)butan-1-one: 12% isolated yield (eluent: EtOAc). ^{1}H NMR (500 MHz, $CDCl_3$, TMS): δ 1.75–2.04 (m, 5H), 2.12–2.23 (m, 1H), 2.43–2.55 (m, 2H), 2.73–2.91 (m, 4H), 3.54–3.61 (m, 1H), 3.74 (s, 6H), 3.80 (s, 3H), 6.07 (s, 2H), 7.26–7.30 (m, 3H), 7.39–7.43 (m, 2H). ^{13}C–{^{1}H} NMR (125 MHz, $CDCl_3$, TMS): δ 22.3, 23.2, 32.0, 43.2, 51.9, 53.2, 55.3, 55.5, 55.9, 84.4, 89.2, 90.7, 113.8, 123.6, 128.0, 128.3, 131.8, 158.2, 162.2, 204.6. MS (70 eV, EI): *m/z* (%): 407 (3) [M^+], 376 (16), 213 (17), 212 (100), 210 (12), 198 (23), 197 (40), 196 (73),195 (75), 170 (34), 169 (25), 156 (10), 153 (10), 152 (12), 137 (11), 129 (10), 128 (22), 120 (17), 115 (17), 102 (12). Anal. Calcd. for $C_{25}H_{29}NO_4$: C, 73.69; H, 7.17; N, 3.44. Found: C, 73.33; H, 6.68; N, 3.21.

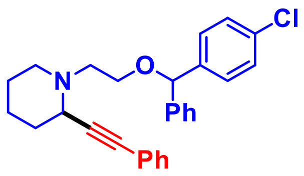

3ra

1-(2-((4-chlorophenyl)(phenyl)methoxy)ethyl)-2-(phenylethynyl)piperidine: 17% isolated yield (eluent: hexane/EtOAc = 8/2 (1st), toluene/EtOAc = 9/1 (2nd)). ^{1}H NMR (500 MHz, $CDCl_3$, TMS): δ 1.50–1.76 (m, 4H), 1.81–1.88 (m, 2H), 2.54–2.70 (m, 2H), 2.79–2.89 (m, 2H), 3.56–3.66 (m, 2H), 3.82–3.86 (m, 1H), 5.37 (s, 1H), 7.22–7.35 (m, 12H), 7.41–7.46 (m, 2H). ^{13}C–{^{1}H} NMR (125 MHz, $CDCl_3$, TMS): δ 20.7, 25.8, 31.5, 49.8, 52.8, 55.8, 67.3, 83.1, 86.4, 87.4, 123.5, 127.0, 127.6, 127.9, 128.2, 128.4, 128.45, 128.47, 131.7, 133.1, 141.0, 141.9. MS (70 eV, EI): *m/z* (%): 431 (7), 430 (6), 429 (19) [M^+], 229 (17), 228 (100), 212 (13), 210 (6), 203 (12), 202 (6), 201 (37), 200 (7), 185 (8), 184 (7), 182 (8), 170 (6), 169 (7), 168 (9), 167 (17), 166 (19), 165 (20), 122 (16), 91 (23), 79 (5). Anal. Calcd. for $C_{28}H_{28}ClNO$: C, 78.21; H, 6.56; N, 3.26. Found: C, 78.20; H, 6.46; N, 3.27.

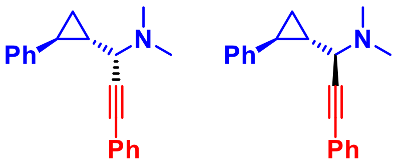

3sa

(S^*)-*N*,*N*-dimethyl-3-phenyl-1-(*trans*-2-phenylcyclopropyl)prop-2-yn-1-amine, (R^*)-*N*,*N*-dimethyl-3-phenyl-1-(*trans*-2-phenylcyclopropyl)prop-2-yn-1-amine: major diastereoisomer: 25% isolated yield (eluent: hexane/EtOAc = 9/1). ^{1}H NMR (500 MHz, $CDCl_3$, TMS): δ 1.03–1.07 (m, 1H), 1.25–1.30 (m, 1H), 1.44–1.50 (m, 1H), 1.97–2.00 (m, 1H), 2.40 (s, 6H), 3.77 (d, J = 5.2 Hz, 1H), 7.06–7.20 (m, 3H), 7.24–7.28 (m, 2H), 7.30–7.36 (m, 3H), 7.44–7.48 (m, 2H). ^{13}C–{^{1}H} NMR (125 MHz, $CDCl_3$, TMS): δ 14.3, 20.5, 25.0, 42.1, 60.9, 84.1, 87.0, 123.2, 125.7, 126.1, 128.3, 128.43, 128.45, 132.0, 142.7. MS (70 eV, EI): *m/z* (%): 275 (10) [M^+], 274 (16), 215 (17), 171 (45), 170 (46), 159 (14), 158 (100), 134 (25), 115 (54), 71 (20). Anal. Calcd. for $C_{20}H_{21}N\cdot0.25H_2O$: C, 85.82; H, 7.74; N, 5.00. Found: C, 86.11; H, 7.82; N, 4.66. minor diastereoisomer: 8% isolated yield (eluent: hexane/EtOAc = 9/1). ^{1}H NMR (500 MHz, $CDCl_3$, TMS): δ 0.97–1.01 (m, 1H), 1.09–1.13 (m, 1H), 1.39–1.44 (m, 1H), 2.11–2.15 (m, 1H), 2.39 (s, 6H), 3.83 (d, J = 5.0 Hz, 1H), 7.12–7.17 (m, 3H), 7.25–7.28 (m, 2H), 7.30–7.34 (m, 3H), 7.43–7.48 (m, 2H). ^{13}C–{^{1}H} NMR (125 MHz, $CDCl_3$, TMS): δ 12.7, 21.2, 25.1, 42.0,

61.1, 83.7, 87.2, 122.8, 123.2, 125.8, 126.3, 128.3, 128.5, 132.0, 142.5. MS (70 eV, EI): *m/z* (%): 275 (3) [M^{+}], 274 (3), 215 (17), 171 (42), 170 (33), 159 (13), 158 (100), 134 (30), 115 (50), 71 (49).

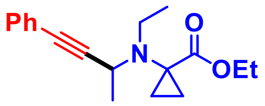

3ta

ethyl 1-(ethyl(4-phenylbut-3-yn-2-yl)amino)cyclopropane-1-carboxylate: 6% isolated yield (eluent: hexane/EtOAc = 19/1 (1st), dichloromethane (2nd)). ^{1}H NMR (500 MHz, $CDCl_3$, TMS): δ 1.13 (t, J = 7.3 Hz, 3H), 1.09–1.21 (m, 2H), 1.23 (t, J = 7.3 Hz, 3H), 1.28–1.40 (m, 2H), 1.42 (d, J = 6.9 Hz, 3H), 2.98–3.15 (m, 2H), 4.07–4.18 (m, 3H), 7.26–7.30 (m, 3H), 7.38–7.40 (m, 2H). ^{13}C–{^{1}H} NMR (125 MHz, $CDCl_3$, TMS): δ 14.3, 16.2, 18.8, 19.5, 23.1, 44.7, 46.1, 50.5, 60.5, 83.5, 91.7, 123.8, 127.6, 128.2, 131.4, 175.4. MS (70 eV, EI): *m/z* (%): 285 (1) [M^{+}], 270 (32), 256 (10), 212 (44), 170 (51), 156 (13), 142 (13), 141 (12), 129 (70), 128 (100), 127 (40), 115 (23), 103 (10), 84 (18), 82 (13), 77 (16), 73 (16), 70 (13), 56 (53), 55 (19), 54 (23).

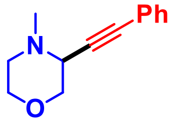

3ua

4-methyl-3-(phenylethynyl)morpholine: 11% GC yield. MS (70 eV, EI): *m/z* (%): 201 (60) [M^{+}], 173 (15), 172 (19), 171 (15), 170 (90), 156 (14), 144 (20), 143 (34), 142 (100), 129 (13), 128 (46), 127 (30), 116 (35), 115 (70), 102 (35), 99 (17), 98 (37), 77 (10), 76 (13), 63 (13), 51 (12).

Supplementary Tables

Supplementary Table 1. Comparison of this work with previous main reports on oxidative α-methylene C–H functionalisations of tertiary amines except for benzylic amines, *N*-protected amines, and symmetric amines.

Reference	Nucleophile or Reaction	Demonstrated tertiary amine substrate type applicable to α-methylene selective functionalization (number of the substrates)	α-Methylene selectivity			Reaction intermediate
			vs Methyl	*vs* Methylene	*vs* Methine	
**this work**	**alkyne**	**linear, cyclic, unsymmetrical (17)**	**methylene**	**cyclic methylene**	**methylene**	**iminium cation**
S12	silylalkyne	cyclic (4)	cyclic methylene	cyclic methylene	—	iminium cation
S13	alkyne	linear, cyclic (3) (low yields:8–16%)	cyclic methylene	—	methylene	iminium cation
S14	iodoalkyne	cyclic (1)	methyl	cyclic methylene	—	amino alkyl radical
S15	dicyanobenzene	cyclic (4)	—	cyclic methylene	methylene	amino alkyl radical
S16	cyclization	cyclic (4) (minor α-methine oxidation)	—	cyclic methylene	methylene	iminium cation
S17	aryl isocyanate	cyclic (3) (minor α-methyl oxidation)	cyclic methylene	—	methylene	amino alkyl radical
S18	ketone	cyclic (1)	cyclic methylene	—	—	iminium cation
S19	cyclization	cyclic, unsymmetrical (6)	methyl	cyclic methylene	methylene	iminium cation
S20	bromoalkene	cyclic (4)	cyclic methylene	both	—	amino alkyl radical
S21	bromobenzene	cyclic (1) (exceptional substrate)	both	—	methyl	amino alkyl radical
S22	alkene	linear, cyclic, unsymmetrical (20) (Curtin–Hammet principle)	methylene	both	methine	amino alkyl radical
S23	NaCN	linear, cyclic (12) (minor α- linear methylene oxidation)	both	cyclic methylene	methylene	iminium cation
S16	NaCN	cyclic (3) (minor α-methyl oxidation)	cyclic methylene	cyclic methylene	—	iminium cation
S24	NaCN	linear, cyclic, unsymmetrical (8) (minor α-methyl, linear methylene oxidation)	cyclic methylene	cyclic methylene	methylene	iminium cation
S25	oxygenation	cyclic, unsymmetrical (3)	cyclic methylene	—	—	iminium cation
S26	oxygenation	cyclic, unsymmetrical (20)	cyclic methylene	cyclic methylene	—	iminium cation
S27	oxygenation	linear, cyclic (11)	methylene	cyclic methylene	—	iminium cation
S13	oxygenation	linear, cyclic (7)	methylene	cyclic methylene	—	iminium cation

Supplementary Table 2. The effect that different supports and Zn cocatalysts have on the yield of the α-methylene-selective alkynylation of 1-methylpiperidine (**1a**) with phenylacetylene (**2a**).[a]

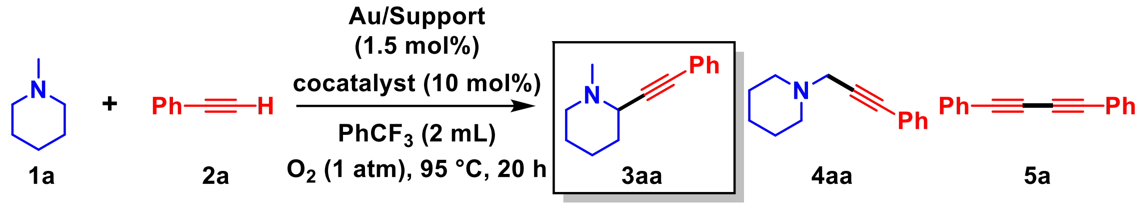

Entry	Catalyst	Cocatalyst	Yield [%]		
			**3aa**	**4aa**	**5a**
1	Au/HAP	$ZnBr_2$	57	<1	<1
2	Au/HAP	ZnF_2	23	<1	<1
3	Au/HAP	$Zn(OAc)_2{\cdot}2H_2O$	12	<1	<1
4	Au/HAP	$Zn(acac)_2$	7	<1	<1
5	Au/HAP	ZnI_2	1	<1	<1
6	Au/ZrO_2	$ZnBr_2$	40	<1	<1
7	Au/CeO_2	$ZnBr_2$	36	<1	<1
8	Au/LDH	$ZnBr_2$	<1	<1	<1
9	—	—	<1	<1	<1

[a]Reaction conditions: **1a** (0.5 mmol), **2a** (0.5 mmol), Au/support (1.5 mol%), Zn cocatalyst (10 mol%), $PhCF_3$ (2 mL), 95 °C, O_2 (1 atm), 20 h. Yields were determined by gas chromatography analysis using biphenyl as an internal standard. —: none.

Supplementary Table 3. The effect that different solvents have on the yield of the α-methylene-selective alkynylation of 1-methylpiperidine (**1a**) with phenylacetylene (**2a**).[a]

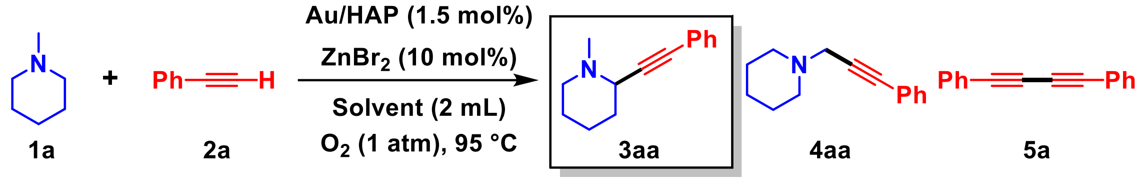

Entry	Solvent	Yield [%]		
		**3aa**	**4aa**	**5a**
1	$PhCF_3$	73	<1	<1
2	Toluene	83	<1	<1
3	PhCl	72	<1	<1
4	Mesitylene	68	<1	<1
5	1,4-Dioxane	68	<1	<1
6	BuOAc	53	<1	<1
7	DMF	8	<1	4
8	DMA	6	<1	4

[a]Reaction conditions: **1a** (1 mmol), **2a** (0.5 mmol), Au/HAP (Au: 1.5 mol%), $ZnBr_2$ (10 mol%), solvent (2 mL), 95 °C, O_2 (1 atm), 20 h. Yields were determined by gas chromatography (GC) analysis using biphenyl as an internal standard. BuOAc: butyl acetate; DMF: *N*,*N*-dimethylformamide; DMA: *N*,*N*-dimethylacetamide.

Supplementary Table 4. Catalyst reuse tests conducted on the α-methylene-selective alkynylation of 1-methylpiperidine (**1a**) with phenylacetylene (**2a**).[a]

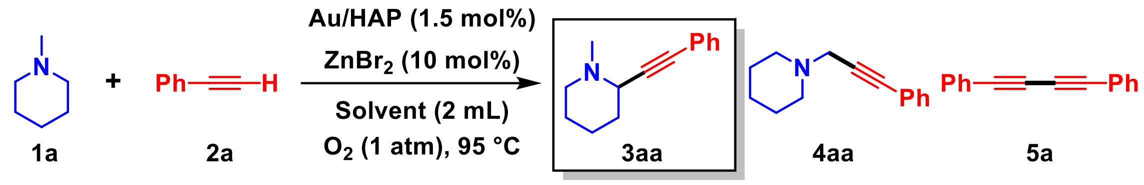

Entry	Catalyst	Yield [%]		
		**3aa**	**4aa**	**5a**
1	Fresh	82	<1	<1
2	1st reuse	82	<1	<1
3	2nd reuse	78	<1	<1

[a]Reaction conditions: **1a** (1 mmol), **2a** (0.5 mmol), Au/HAP (100 mg, Au: 1.5 mol%), $ZnBr_2$ (10 mol%), toluene (2 mL), 95 °C, O_2 (1 atm), 24 h. Yields were determined by gas chromatography analysis using biphenyl as internal standard. The average values calculated over 8, 5, and 3 runs are reported for entries 1, 2, and 3, respectively.

Supplementary Table 5. Optimization of reaction conditions for α-methine alkynylation of *N*,*N*-dimethylcyclohexylamine (**1m**) with **2a**.[a]

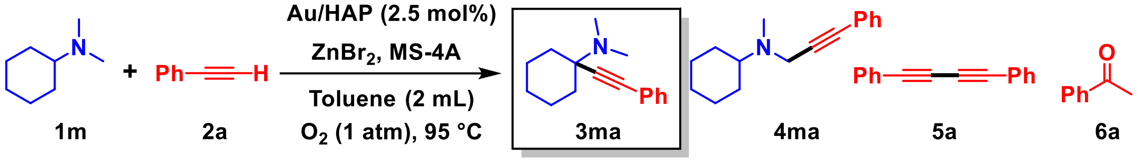

Entry	**2a** [eq.]	$ZnBr_2$ [mol%]	MS-4A [mg]	Conv. of **1m** [%]	Yield [%] **3ma**	**4ma**	**5a**	**6a**
1	2	20	0	89	12	1	3	19
2	4	20	0	77	16	1	1	11
3	6	20	0	87	23	1	1	7
4	6	40	0	99	35	<1	<1	10
5	6	20	100	72	28	1	<1	<1
6	6	20	200	84	52	1	<1	<1
7	6	20	300	97	84	<1	<1	<1
8	6	40	300	73	39	<1	1	<1

[a]Reaction conditions: **1m** (0.3 mmol), **2a** (0.6, 1.2, or 1.8 mmol), Au/HAP (100 mg, Au: 2.5 mol%), $ZnBr_2$ (20 or 40 mol%), MS-4A (0, 100, 200, or 300 mg), toluene (2 mL), 95 °C, O_2 (1 atm), 24 h. Conversions and yields were determined by gas chromatography analysis using biphenyl as internal standard. MS-4A = molecular sieves 4A.

Supplementary Table 6. Comparison of **1a** conversions with/without **2a** and/or $ZnBr_2$.[a]

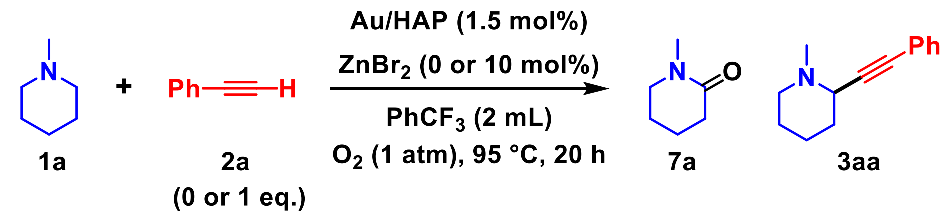

Entry	**2a** [eq.]	$ZnBr_2$ [mol%]	Conv. of **1a** [%]	Yield [%]	
				**7a**	**3aa**
1	0	0	18	<1	—
2	0	10	26	<1	—
3	1	0	36	<1	18
4	1	10	77	<1	57

[a]Reaction conditions: **1a** (0.5 mmol), **2a** (0 or 0.5 mmol), Au/HAP (Au: 1.5 mol%),$ZnBr_2$ (0 or 10 mol%), $PhCF_3$ (2 mL), 95 °C, O_2 (1 atm), 20 h. Conversions and yields were determined by gas chromatography using biphenyl as an internal standard.

Supplementary Figures

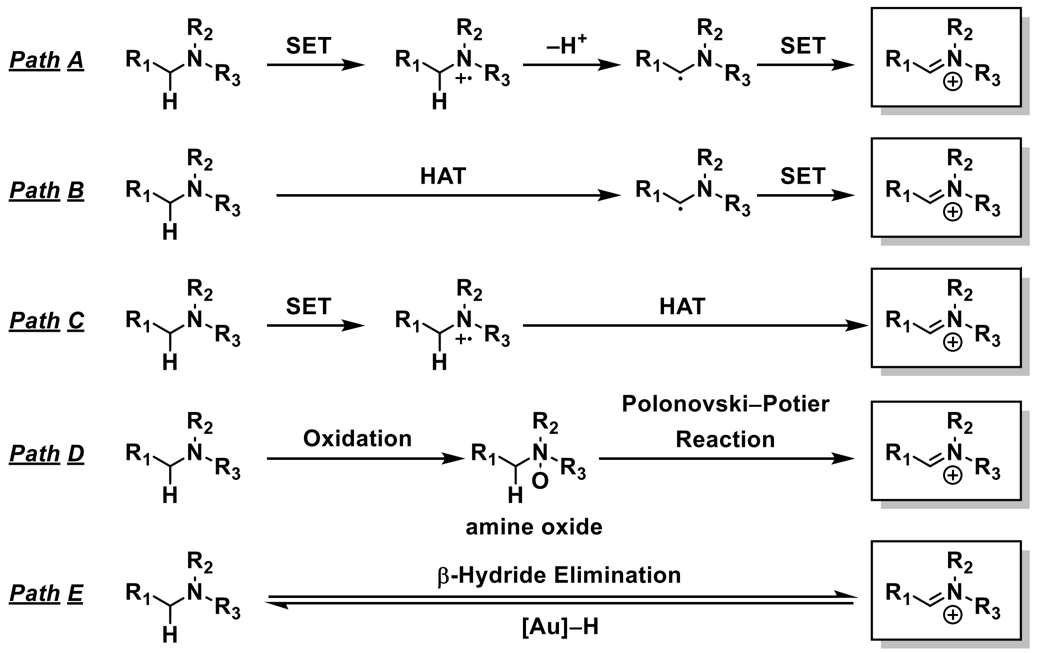

Supplementary Fig. 1 Proposed paths of amine oxidation to iminium cations based on previous reports; path A: single-electron-transfer (SET)/deprotonation/SET, path B: hydrogen-atom-transfer (HAT)/SET, path C: SET/HAT, path D: amine oxidation to amine oxides followed by Polonovski–Potier reaction, and path E: β-hydride elimination.

(a)

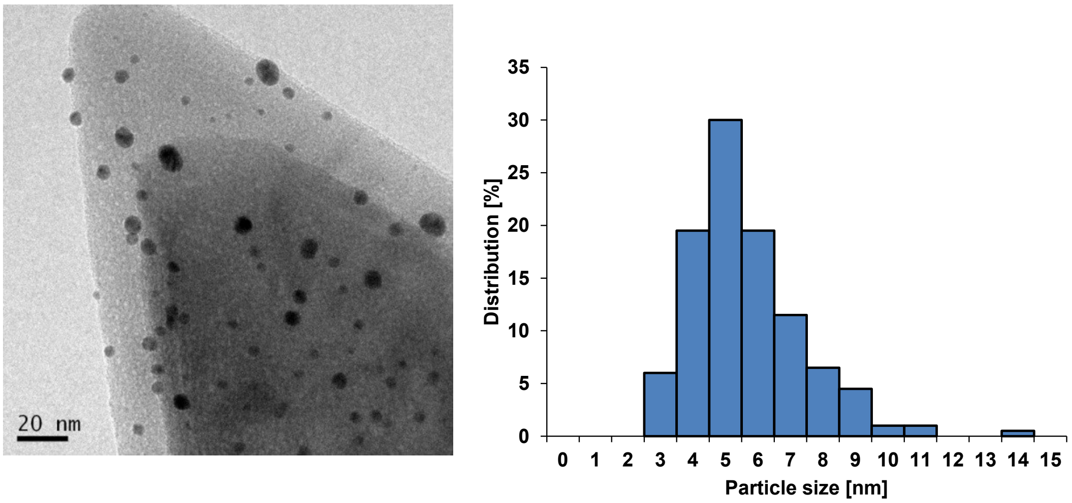

(b)

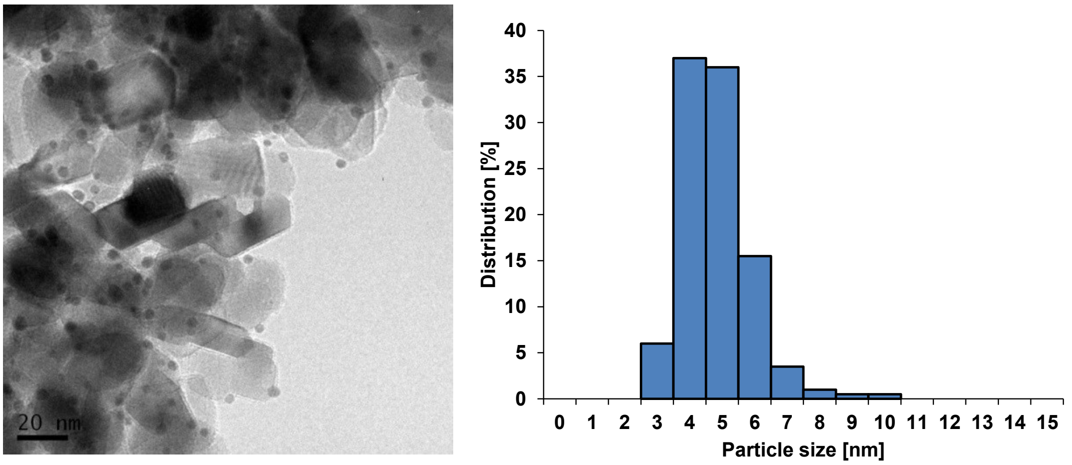

Supplementary Fig. 2 TEM images and nanoparticle size distributions of (a) Au/HAP (mean diameter: 5.1 nm, σ= 1.7 nm) and (b)Au/TiO_2 (mean diameter: 4.3 nm, σ= 1.0 nm). The size distributions were determined using 200 particles.

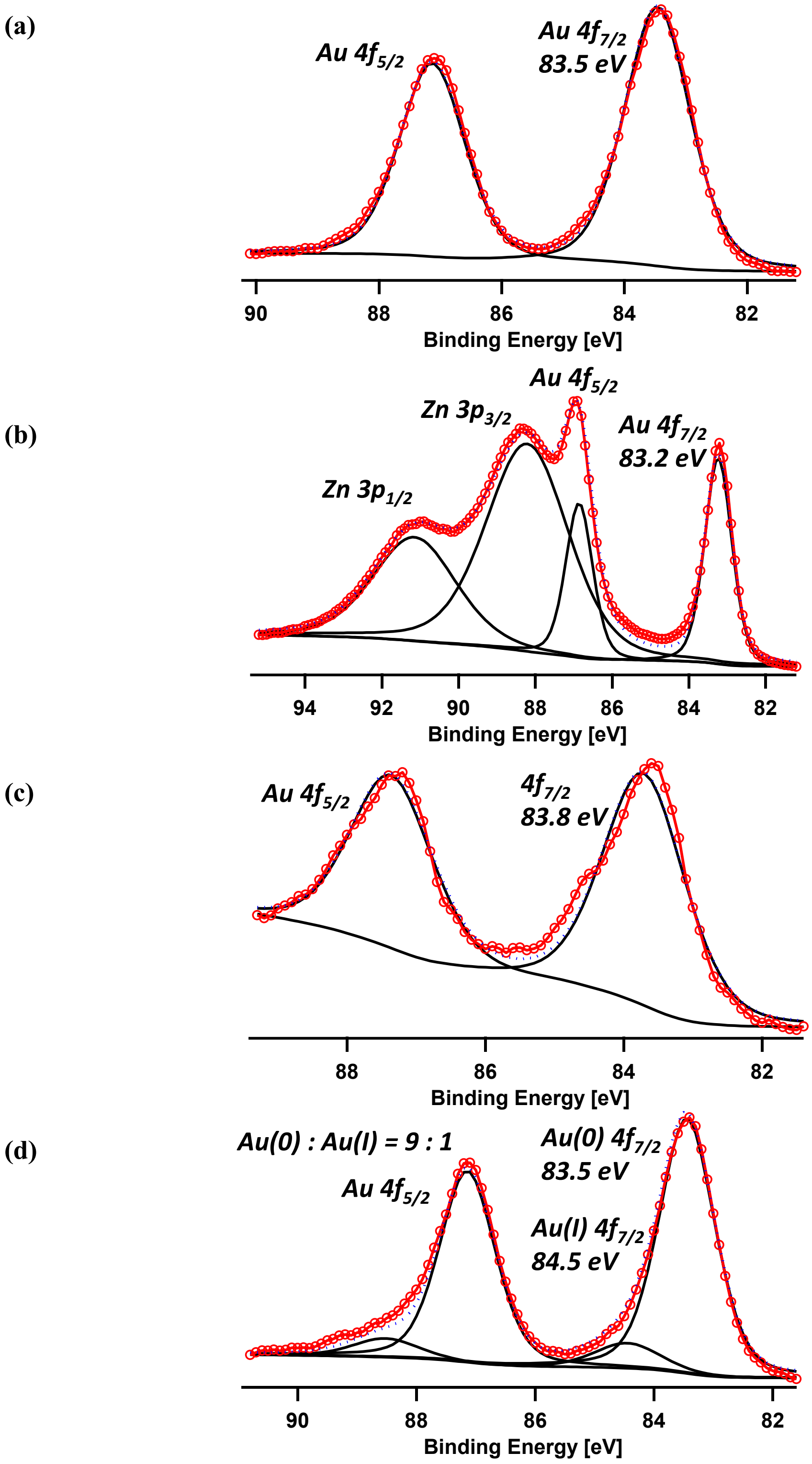

Supplementary Fig. 3 X-ray photoelectron spectra around Au *4f* components: (a) Au/HAP, (b) Au/ZnO, (c) Au/Al_2O_3, and (d) Au/ZrO_2. Black lines and blue broken lines indicate the deconvoluted signals and the sum of these lines. Red dotted lines indicate the data plots. The binding energies were calibrated by using the C *1s* signal at 284.8 eV.

(e)

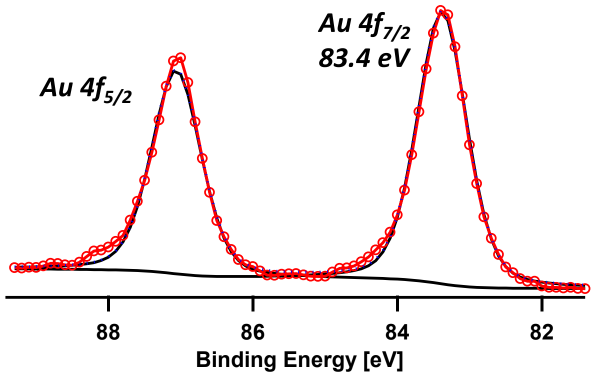

(f)

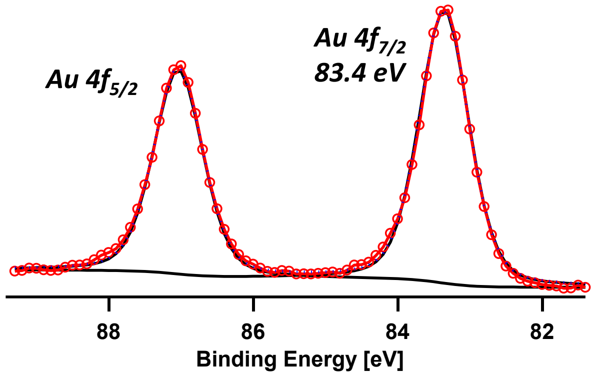

(g)

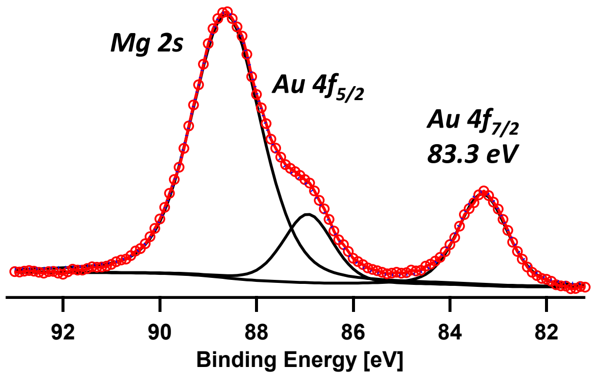

Supplementary Fig. 3 (continued) X-ray photoelectron spectra around Au *4f* components: (e) Au/TiO_2, (f) Au/CeO_2, and (g) Au/LDH. Black lines and blue broken lines indicate the deconvoluted signals and the sum of these lines. Red dotted lines indicate the data plots. The binding energies were calibrated by using the C *1s* signal at 284.8 eV.

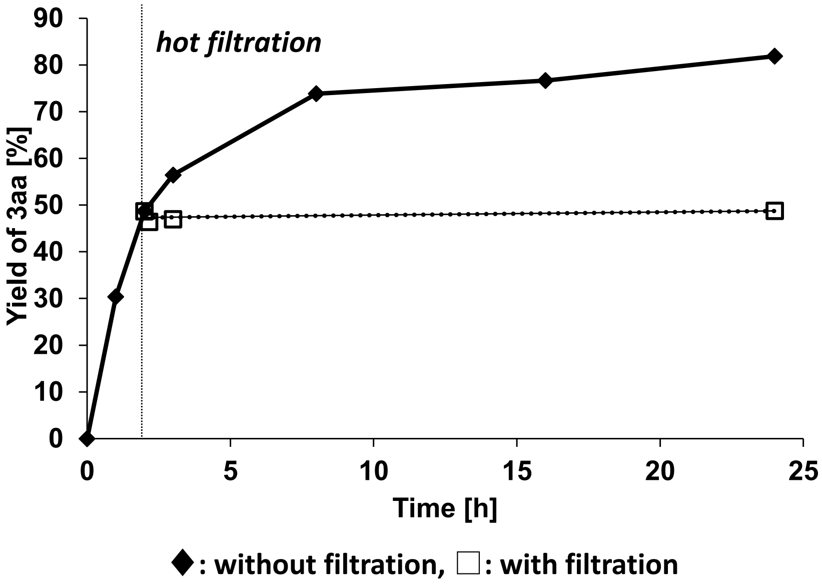

Supplementary Fig. 4 Influence of the mid-reaction removal by hot filtration of catalyst Au/HAP on the profile of the reaction of 1-methylpiperidine (**1a**) with phenylacetylene (**2a**) to produce 1-methyl-2-(phenylethynyl)piperidine (**3aa**) (verification of heterogeneous catalysis). Reaction conditions: **1a** (1 mmol), **2a** (0.5 mmol), Au/HAP (100 mg), $ZnBr_2$ (10 mol%), toluene (2 mL), 95 °C, O_2 (1 atm). Yields were determined by gas chromatography analysis using biphenyl as internal standard.

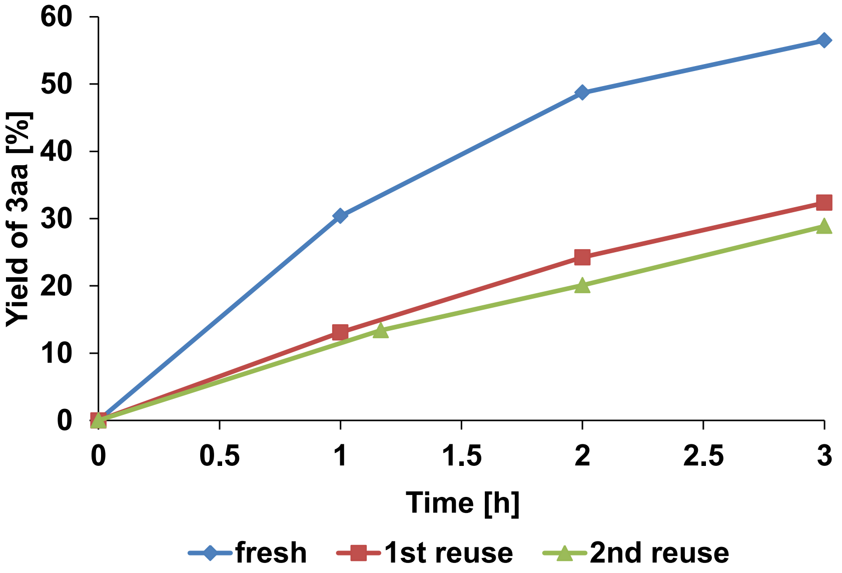

Supplementary Fig. 5 Reaction profiles obtained for catalyst reuse tests. Reaction conditions: 1-methylpiperidine (**1a**, 1 mmol), phenylacetylene (**2a**, 0.5 mmol), Au/HAP (100 mg), $ZnBr_2$ (10 mol%), toluene (2 mL), 95 °C, O_2 (1 atm). Yields were determined by gas chromatography analysis using biphenyl as internal standard.

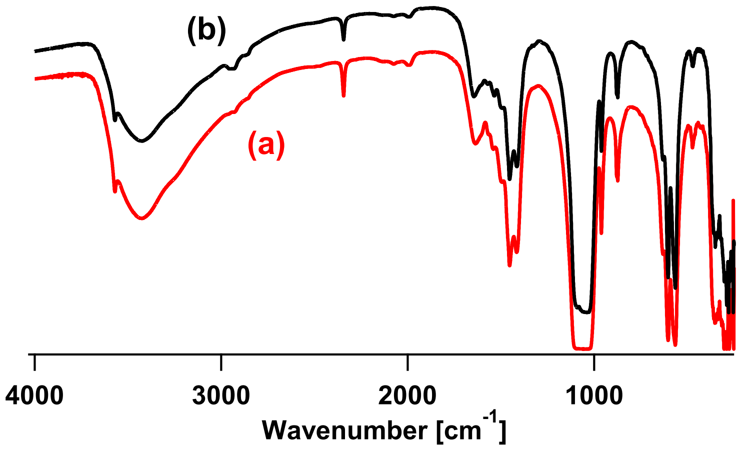

Supplementary Fig. 6 FT-IR spectra of (a) Au/HAP retrieved after the 1st use, and (b) fresh Au/HAP.

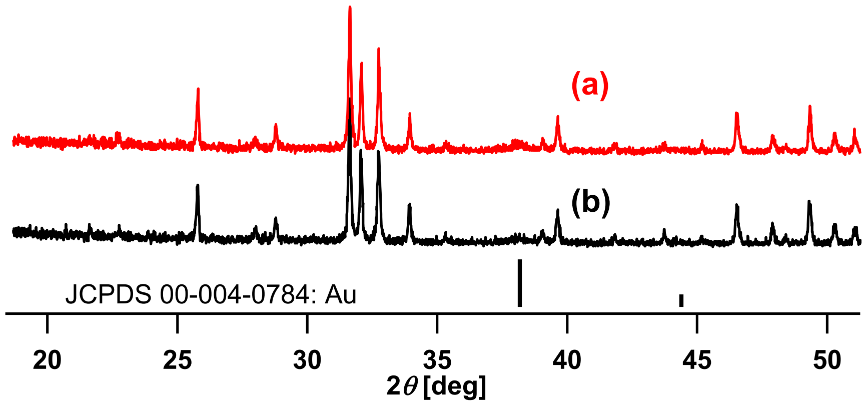

Supplementary Fig. 7 X-ray diffraction (XRD) patterns of (a) Au/HAP retrieved after the 1st use, and (b) fresh Au/HAP.

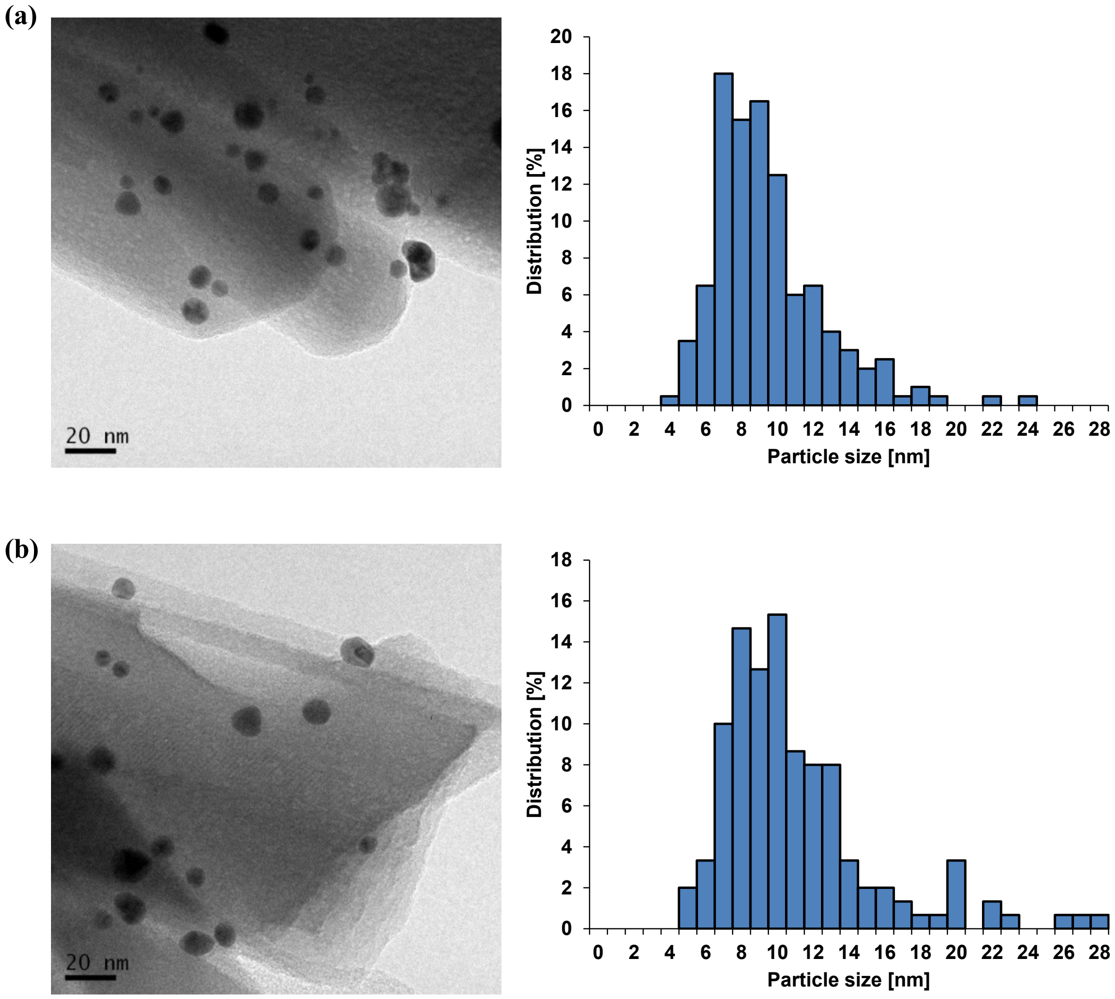

Supplementary Fig. 8 TEM images and nanoparticle size distributions of (a) Au/HAP retrieved after the 1st use (mean diameter: 9.0 nm, σ = 3.2 nm) and (b) Au/HAP retrieved after the 3rd use (mean diameter: 10.5 nm, σ = 4.3 nm). The size distributions were determined using 200 particles.

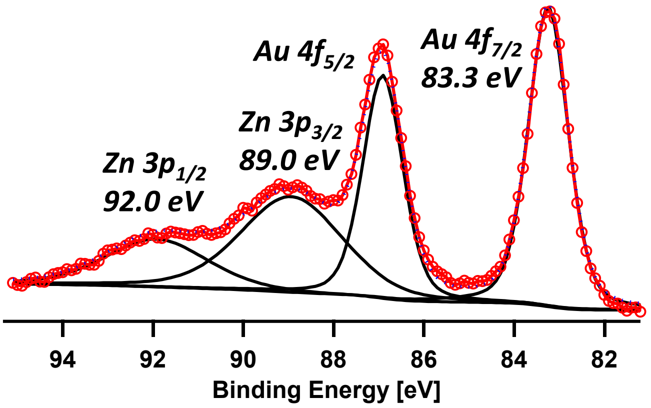

Supplementary Fig. 9 X-ray photoelectron spectra of Au/HAP retrieved after the 1st use around the Au *4f* region. Black lines and blue broken lines indicate the deconvoluted signals and the sum of these lines. Red dotted lines indicate the data plots. The binding energies were calibrated by using the C *1s* signal at 284.8 eV.

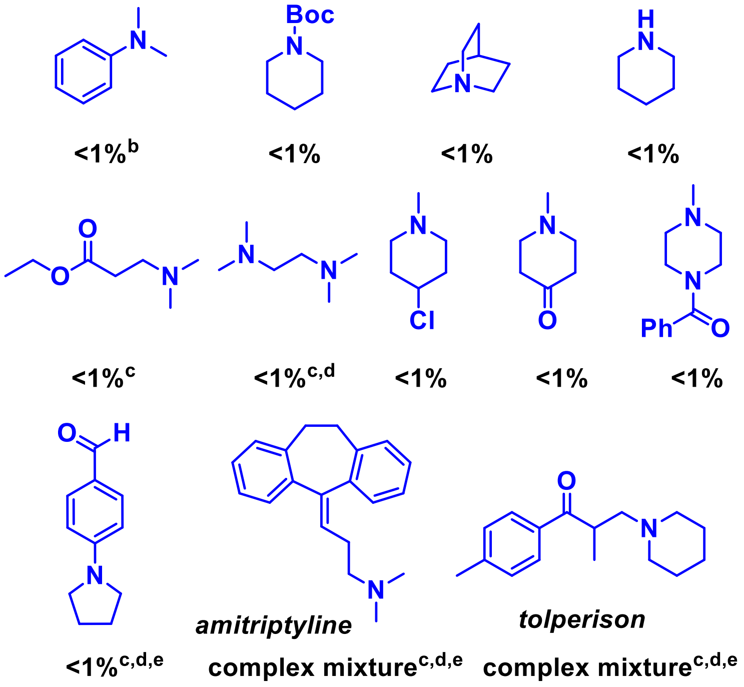

Supplementary Fig. 10 Limitation of amine substrate scope.[a] Yields were determined by GC or ^{1}H NMR analysis. [a]Reaction conditions: **1** (1 mmol), **2a** (0.5 mmol), Au/HAP (100 mg, Au: 1.5 mol%), $ZnBr_2$ (11 mg, 10 mol%), $PhCF_3$ (2 mL), 95 °C, O_2 (1 atm), 24 h. [b]20 h. [c]Toluene (2 mL). [d]**1** (0.3 mmol), **2a** (0.6 mmol), Au/HAP (160 mg, 4 mol%), $ZnBr_2$ (13 mg, 20 mol%). [e]**2a** (1.8 mmol), MS-4A (300 mg).

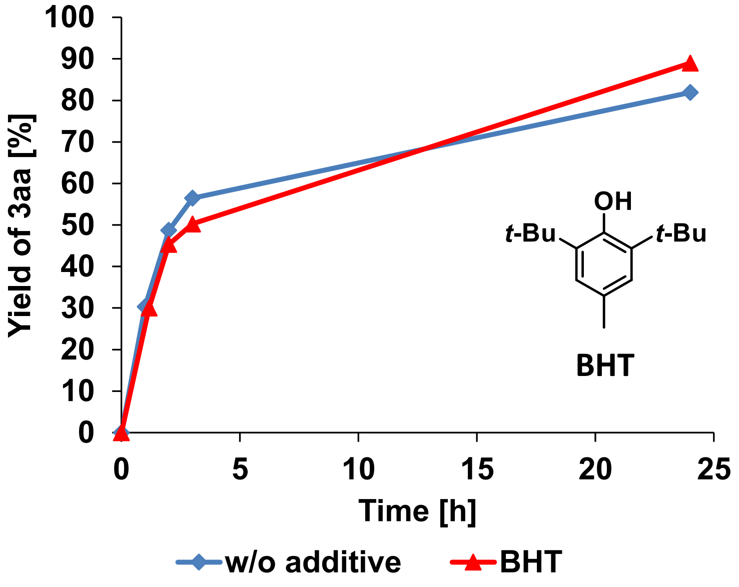

Supplementary Fig. 11 The effect of a radical scavenger (1 mmol, 1 equivalent with respect to **1a**) on the α-methylene-selective alkynylation of 1-methylpiperidine with phenylacetylene. The reaction conditions were the same as those described in Supplementary Fig. 4. GC-determined yields of 1-methyl-2-(phenylethynyl)piperidine (**3aa**) are shown in the vertical axis. BHT: 2,6-di-*tert*-butyl-4-methylphenol.

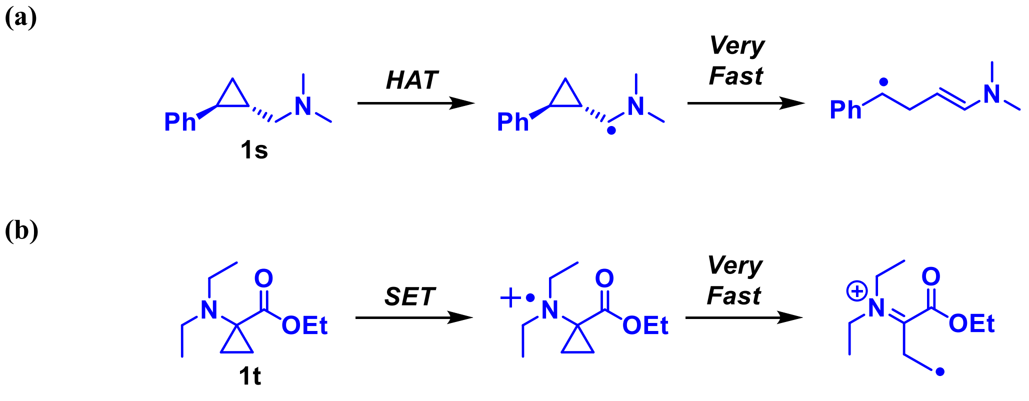

Supplementary Fig. 12 Ring-opening reactions of radical clocks (a) **1s** or (b) **1t** when HAT or SET of the amines occurs, respectively.

(a)

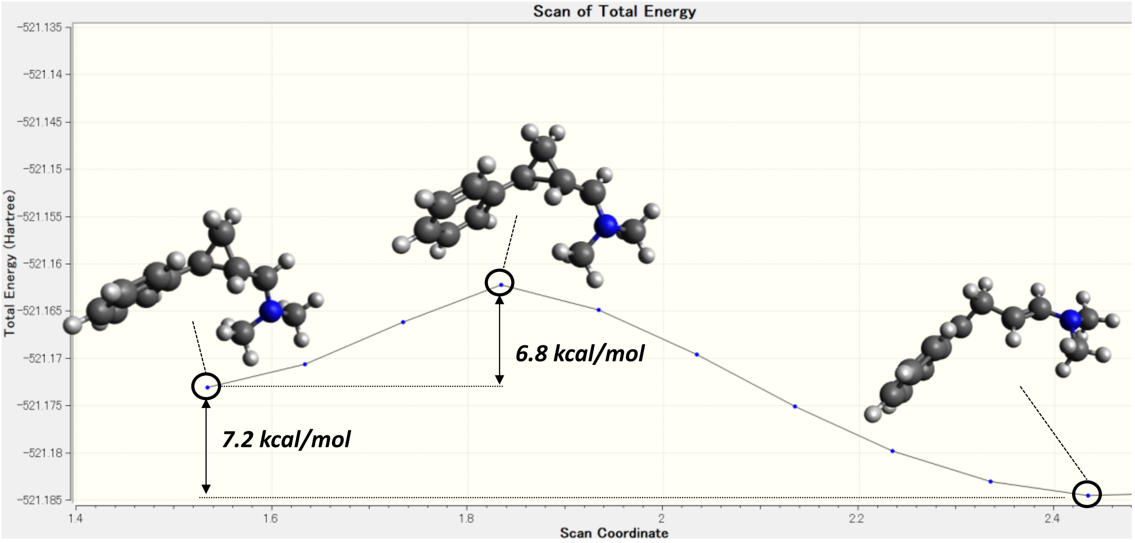

(b)

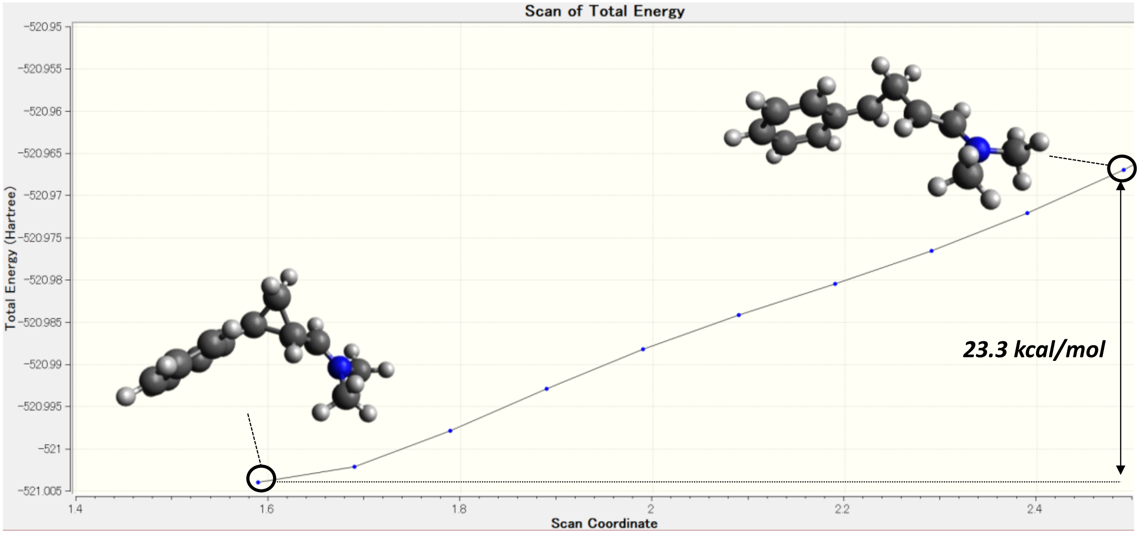

Supplementary Fig. 13 DFT scan calculation using Gaussian about ring-opening reactions of the corresponding (a) carbon-centered radical species and (b) iminium cation species derived from **1s**, respectively.

(a)

(b)

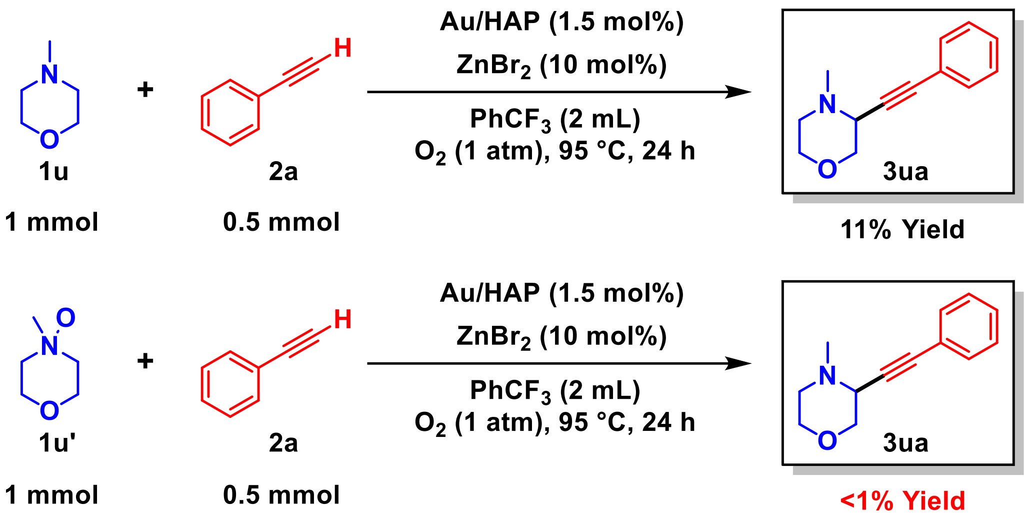

Supplementary Fig. 14 Reactions to obtain an α-methylene alkynylated product (**3ua**) with **2a** using the present hybrid catalytic system comprising Au/HAP and $ZnBr_2$ starting from (a) 4-methylmorpholine (**1u**) or (b) 4-methylmorpholine *N*-oxide (**1u'**). Reaction conditions are indicated in the figure, and the yields were determined by GC using biphenyl as internal standard.

(a)
(b)
(c)
(d)
(e)

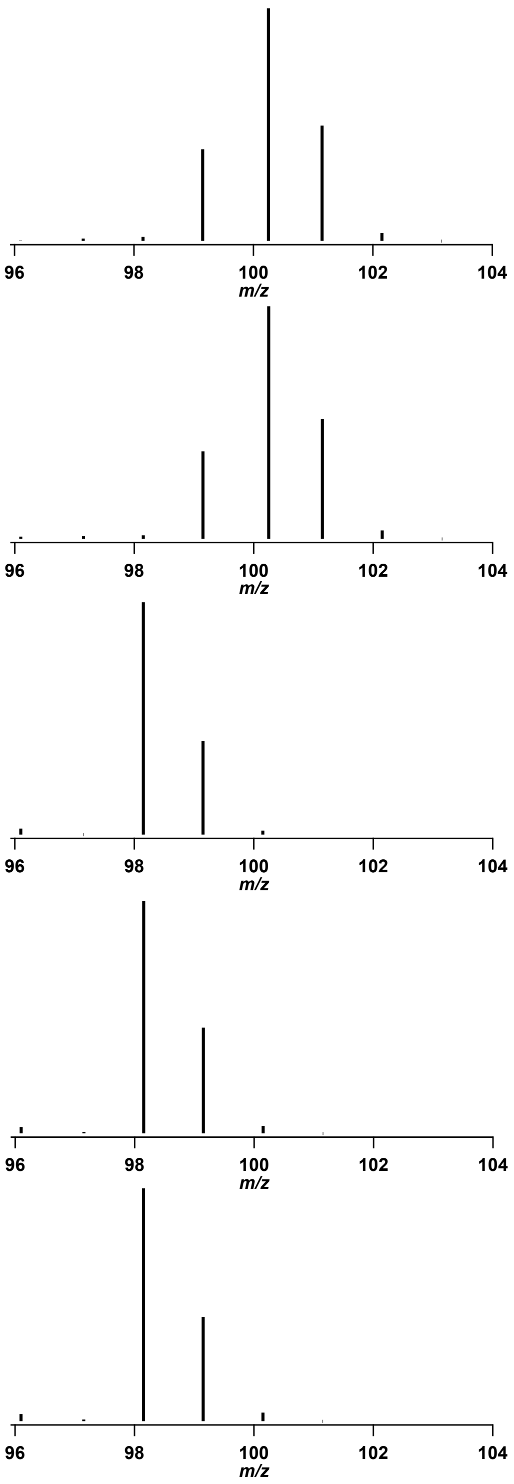

Supplementary Fig. 15 GC-MS patterns obtained by selected ion monitoring mode for *m/z* = 96–103 to detect the deuterium scrambling using several substrates under the reaction conditions indicated in Fig. 2b: (a) **1a-*d*$_2$**, (b) **1a-*d*$_2$** after the reaction only with Au/HAP, (c) **1a**, (d) **1a** after the reaction with $NaBD_4$ and Au/HAP, and (e) **1a** after the reaction with D_2O and Au/HAP.

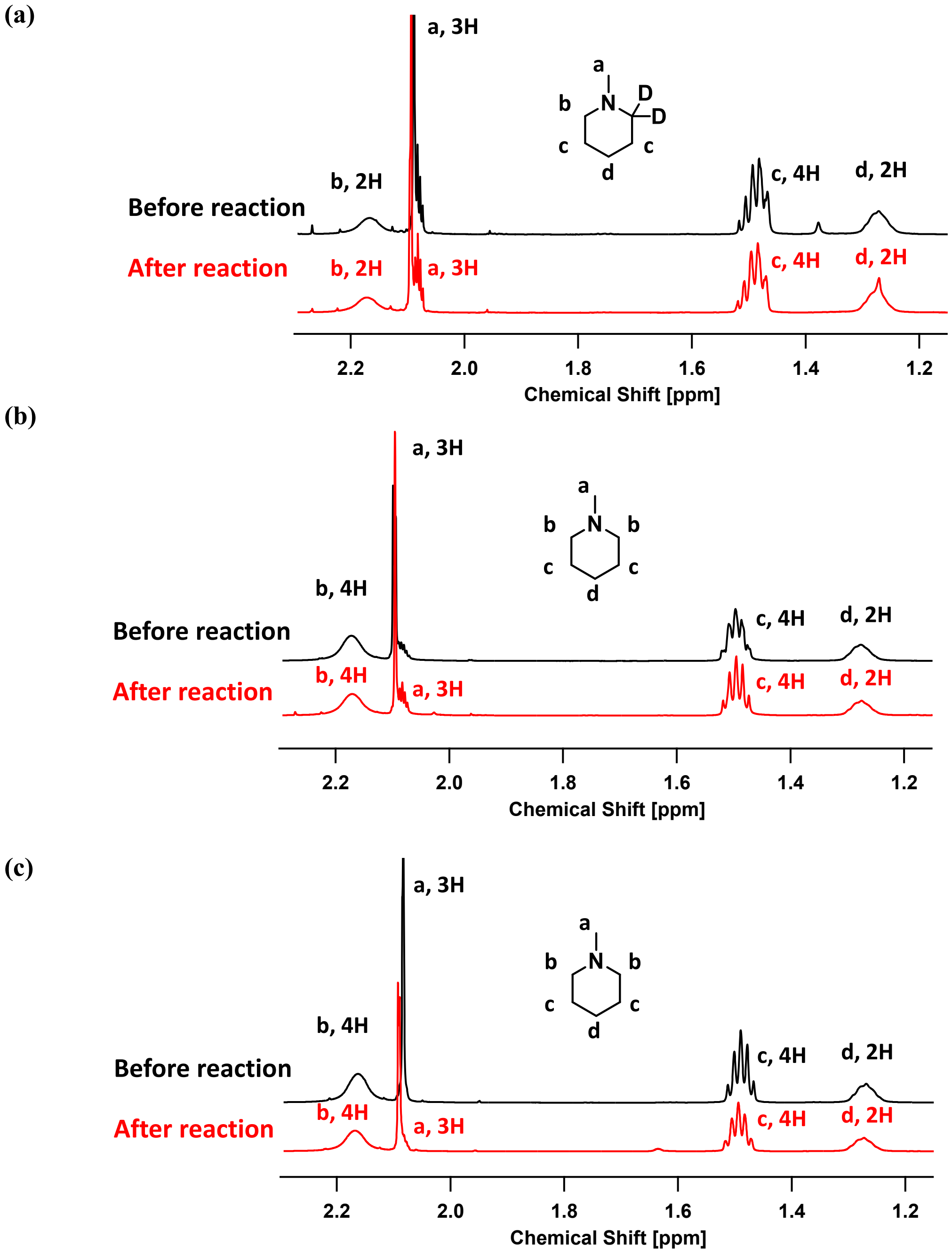

Supplementary Fig. 16 ^{1}H NMR spectra to detect the deuterium scrambling using several substrates and toluene-d_8 solvent under the reaction conditions indicated in Fig. 2b: (a) the spectra of **1a-*d*$_2$** before/after the reaction only with Au/HAP, (b) the spectra of **1a** before/after the reaction with $NaBD_4$ and Au/HAP, and (c) the spectra of **1a** before/after the reaction with D_2O and Au/HAP, recorded at ~25 °C in toluene-d_8 at 500 MHz.

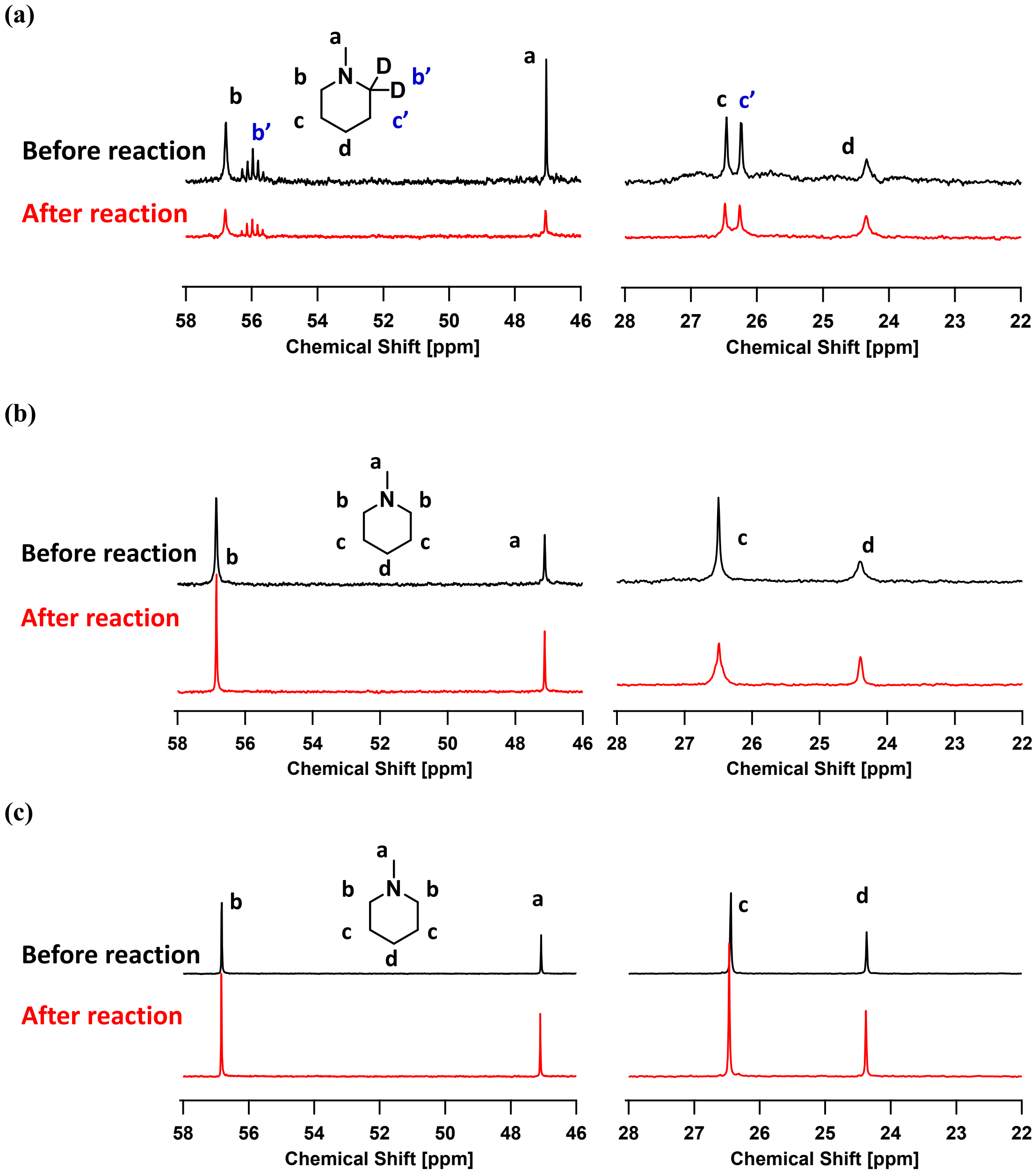

Supplementary Fig. 17 ^{13}C NMR spectra to detect the deuterium scrambling using several substrates and toluene-d_8 solvent under the reaction conditions indicated in Fig. 2b: (a) the spectra of **1a-*d*₂** before/after the reaction only with Au/HAP, (b) the spectra of **1a** before/after the reaction with $NaBD_4$ and Au/HAP, and (c) the spectra of **1a** before/after the reaction with D_2O and Au/HAP, recorded at ~25 °C in toluene-d_8 at 126 MHz.

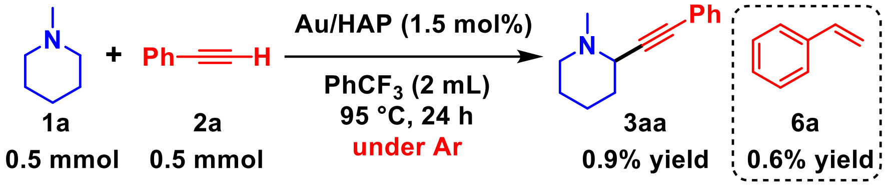

Supplementary Fig. 18 α-Alkynylation of **1a** with **2a** in the presence of Au/HAP without any cocatalysts under an Ar atmosphere. Reaction conditions are indicated in the figure, and the yields were determined by GC using biphenyl as internal standard.

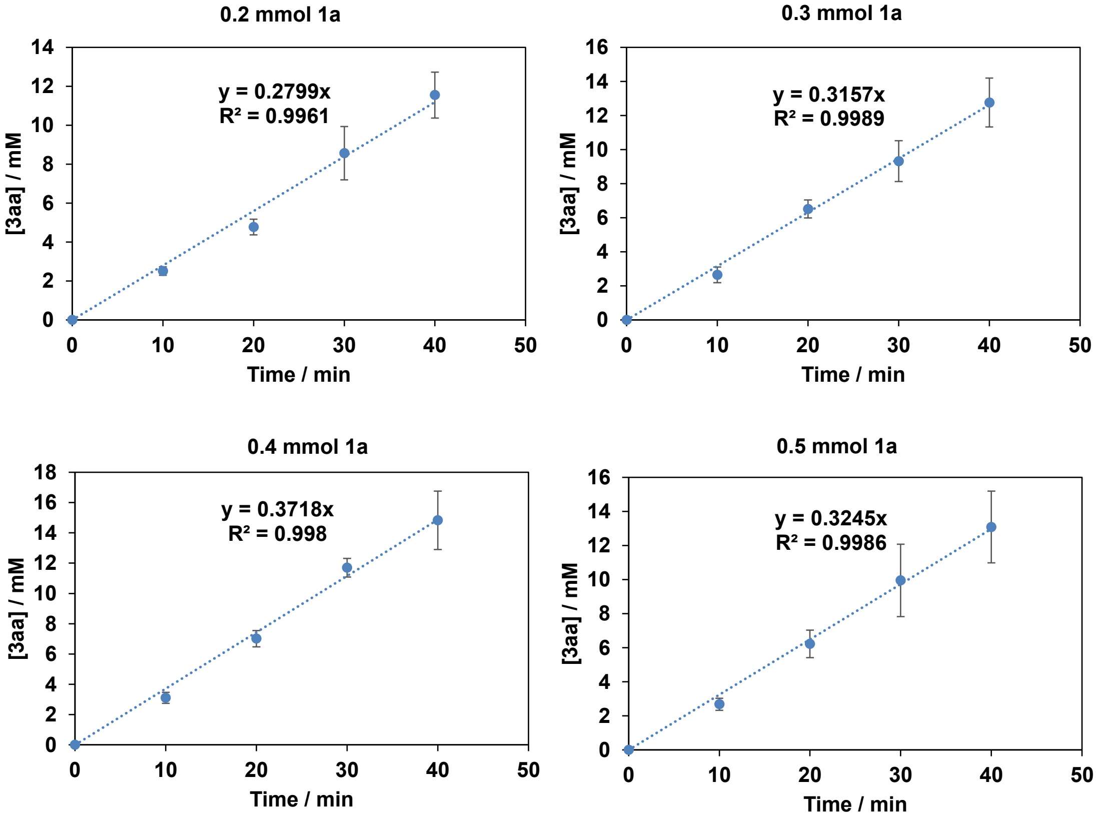

Supplementary Fig. 19 The dependence of the initial condensation of **1a** (0.1 mM, 0.15 mM, 0.2 mM, or 0.25 mM) on **3aa** production rate using the present hybrid catalytic system comprising Au/HAP and $ZnBr_2$. Reaction conditions: **1a** (0.2, 0.3, 0.4, or 0.5 mmol), **2a** (1 mmol), Au/HAP (0.8 mol% to **2a**), $ZnBr_2$ (5 mol% to **2a**), $PhCF_3$ (2 mL), 95 °C, open air (1 atm). Yields were determined by gas chromatography analysis using biphenyl as an internal standard. The data plots are average values of 2 runs, and the error bars calculated from 2 runs show the maximum/minimum values of the respective yields.

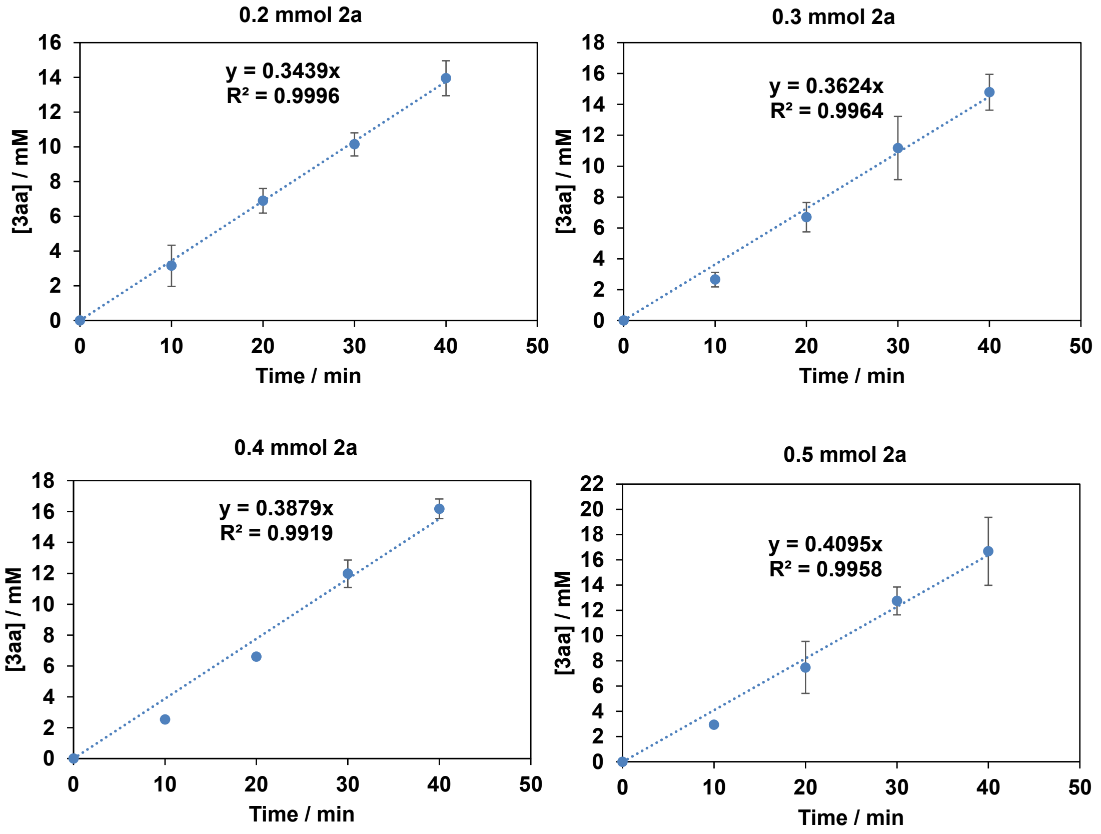

Supplementary Fig. 20 The dependence of the initial condensation of **2a** (0.1 mM, 0.15 mM, 0.2 mM, or 0.25 mM) on **3aa** production rate using the present hybrid catalytic system comprising Au/HAP and $ZnBr_2$. Reaction conditions: **1a** (1 mmol), **2a** (0.2, 0.3, 0.4, or 0.5 mmol), Au/HAP (0.8 mol% to **1a**), $ZnBr_2$ (5 mol% to **1a**), $PhCF_3$ (2 mL), 95 °C, open air (1 atm). Yields were determined by gas chromatography analysis using biphenyl as an internal standard. The data plots are average values of 2 runs, and the error bars calculated from 2 runs show the maximum/minimum values of the respective yields.

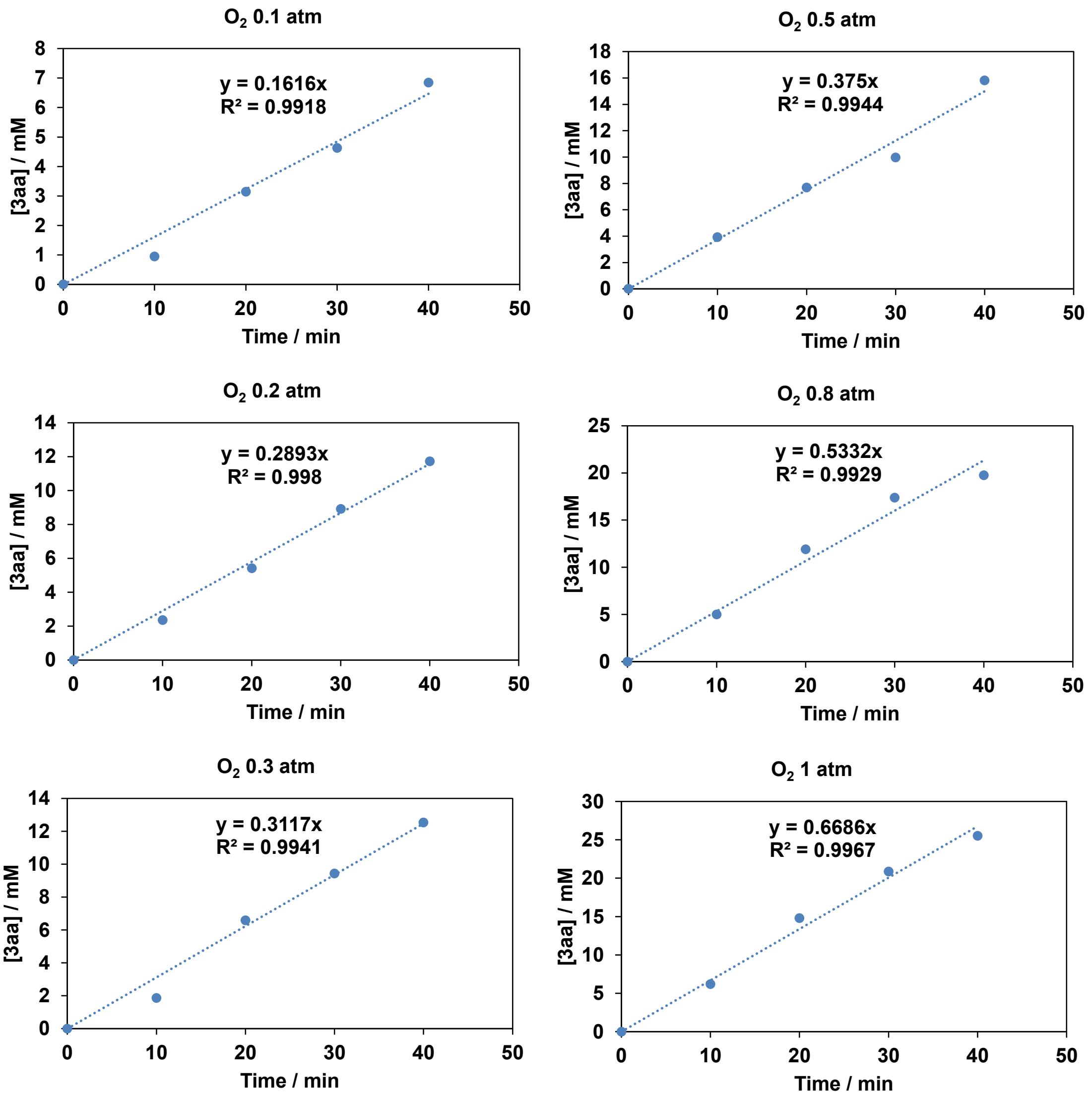

Supplementary Fig. 21 The dependence of O_2 partial pressure (0.1 atm, 0.2 atm, 0.3 atm, 0.5 atm, 0.8 atm, or 1 atm) on **3aa** production rate from **1a** and **2a** using the present hybrid catalytic system comprising Au/HAP and $ZnBr_2$. Reaction conditions: **1a** (0.5 mmol), **2a** (0.5 mmol), Au/HAP (1.5 mol%), $ZnBr_2$ (10 mol%), $PhCF_3$ (2 mL), 95 °C. Yields were determined by gas chromatography analysis using biphenyl as an internal standard.

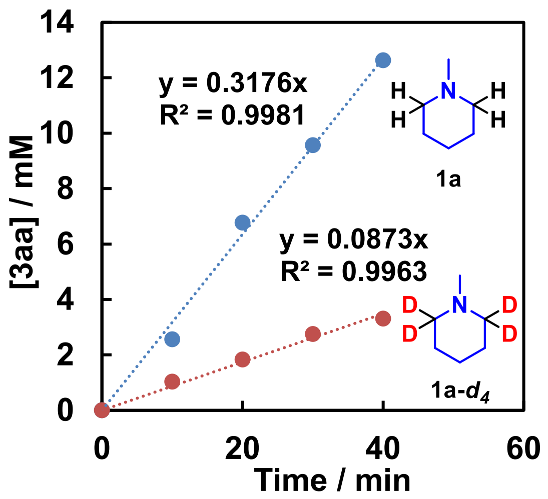

Supplementary Fig. 22 Kinetic isotope effect on α-alkynylation with **2a** in the presence of Au/HAP and $ZnBr_2$ using **1a** or **1a-*d₄*** as an amine substrate, respectively. Reaction conditions are indicated in Fig. 2e, and the yields were determined by GC using biphenyl as internal standard.

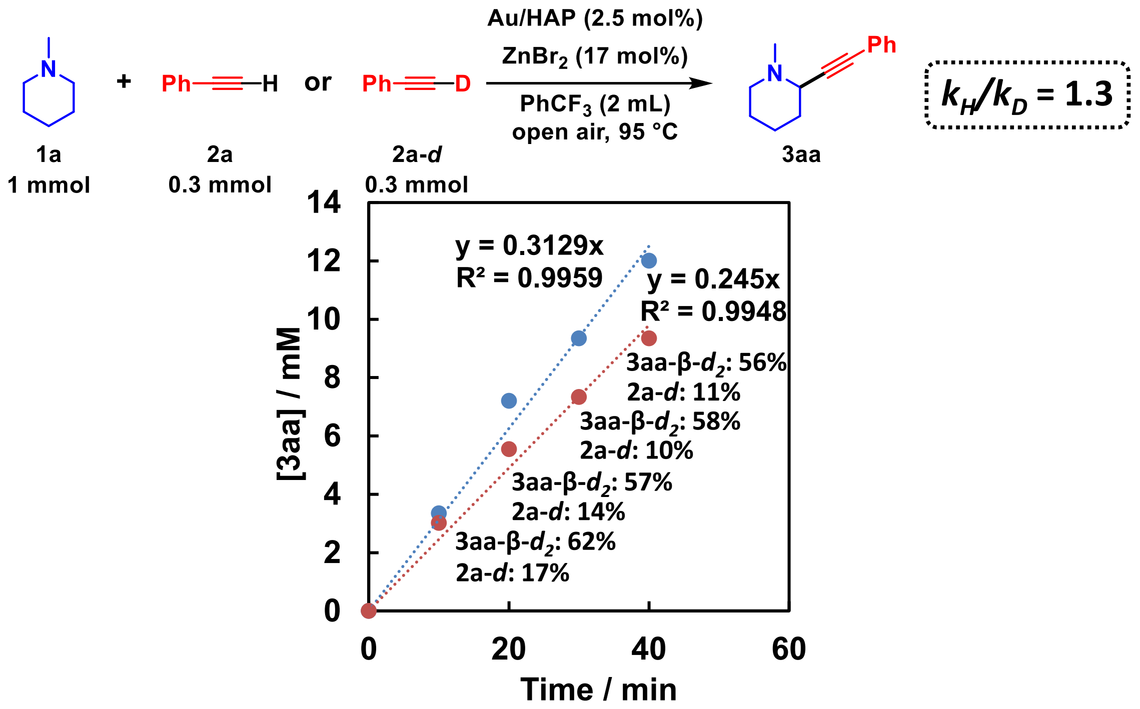

Supplementary Fig. 23 Kinetic isotope effect investigation on α-alkynylation of **1a** in the presence of Au/HAP and $ZnBr_2$ using **2a** or **2a-*d*** as an alkyne substrate, respectively. Reaction conditions are indicated in this figure, and the yields were determined by GC using biphenyl as internal standard. GC-MS spectra were recorded in scan mode, and deuteration ratios of each sampling were approximately estimated from the relative intensity of m/z = 103 based on those of **2a** and **2a-*d*** (D: 98%) and from the relative intensity of m/z = 200 based on those of **3aa** and **3aa-*d₂*** (D: 13% and 76%).

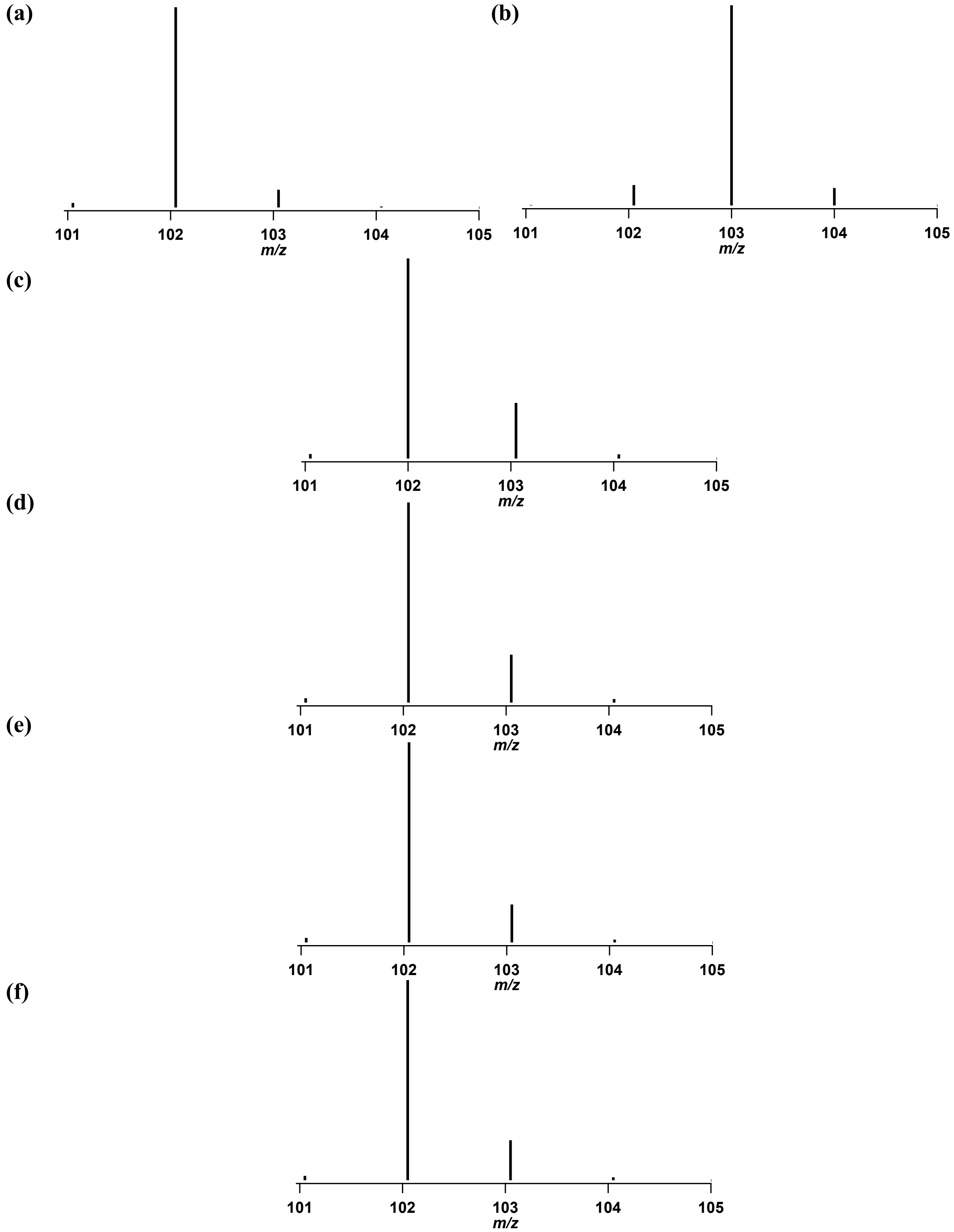

Supplementary Fig. 24 GC-MS patterns of **2a** obtained by scan mode for *m/z* = 101–105 to detect the deuterium scrambling using **2a-*d*** as an alkyne substrate under the reaction conditions indicated in Supplementary Fig. 23: (a) **2a** and (b) **2a-*d*** (D: 98% determined by ^{1}H NMR). (c) 10 min, (d) 20 min, (e) 30 min, and (f) 40 min after the reaction started.

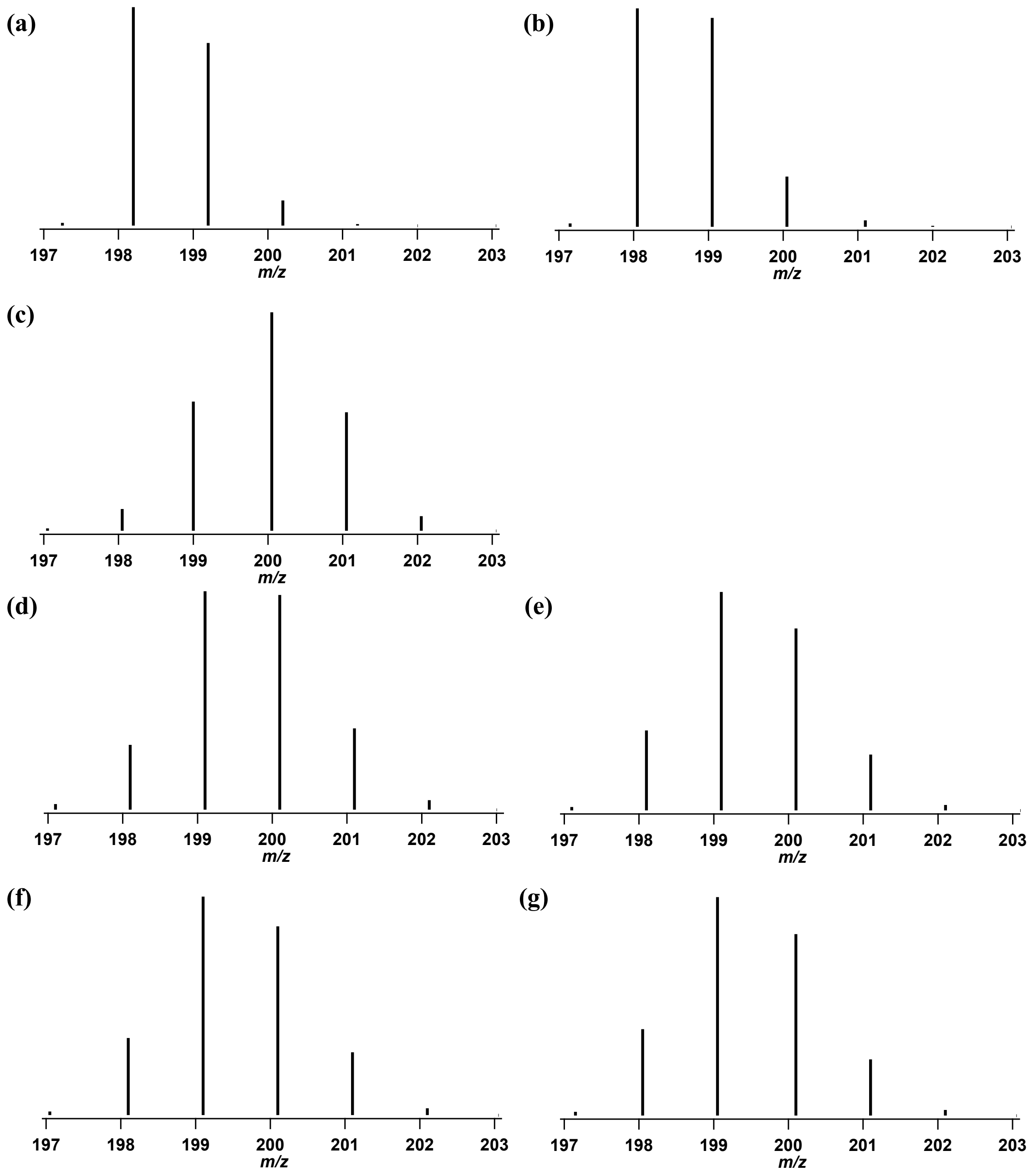

Supplementary Fig. 25 GC-MS patterns of **3aa** obtained by scan mode for *m/z* = 197–203 to detect the deuterium scrambling using **2a-*d*** as an alkyne substrate or D_2O as an additive under the reaction conditions indicated in Supplementary Fig. 23 or 28: (a) **3aa**, (b) **3aa-β-*d*₂** (D: 13% determined by ^{1}H NMR) isolated 24 h after the reaction using **2a-*d*** as an alkyne substrate, (c) **3aa-β-*d*₂** (D: 76% determined by ^{1}H NMR) isolated 25 h after the reaction using D_2O as an additive. (d) 10 min, (e) 20 min, (f) 30 min, and (g) 40 min after the reaction using **2a-*d*** as an alkyne substrate started.

(a)

(b)

(c)

(d)

(e)

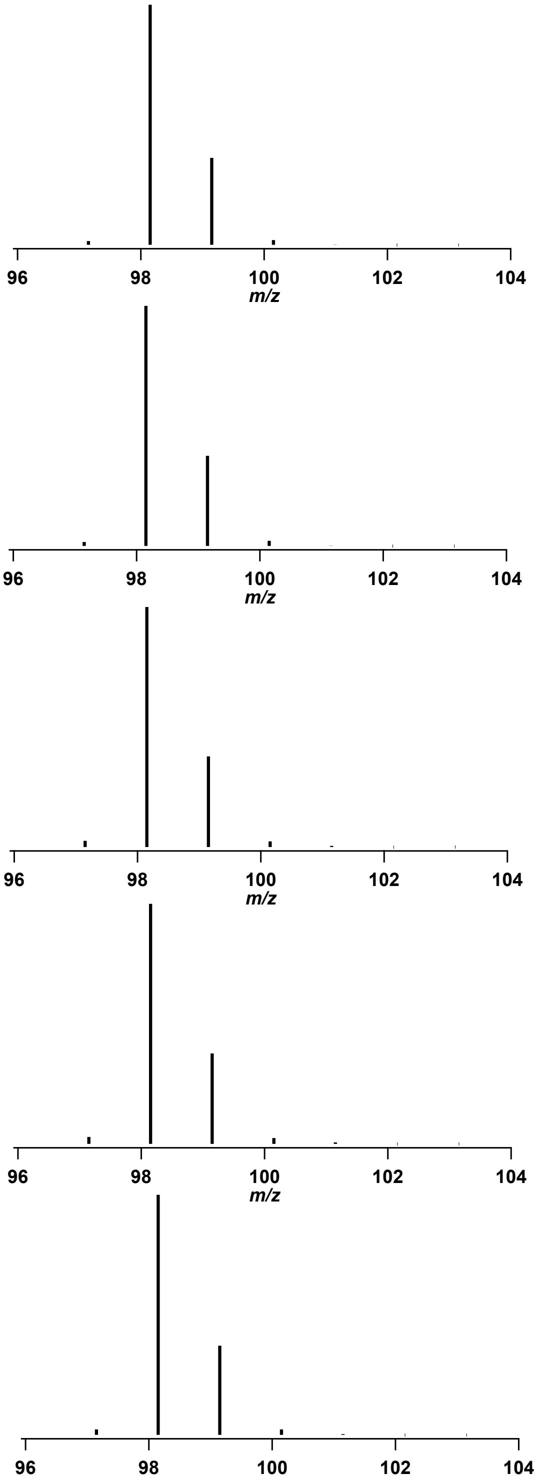

Supplementary Fig. 26 GC-MS patterns of **1a** obtained by selected ion monitoring mode for *m/z* = 97–103 to detect the deuterium scrambling using **2a-*d*** as an alkyne substrate under the reaction conditions indicated in Supplementary Fig. 23: (a) **1a**. (b) 10 min, (c) 20 min, (d) 30 min, and (e) 40 min after the reaction started.

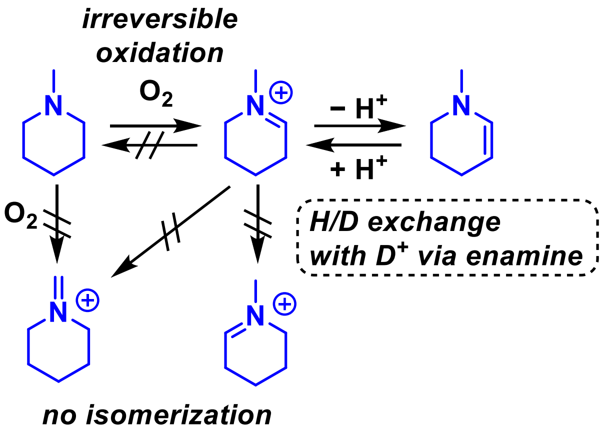

Supplementary Fig. 27 Proposed mechanism of the corresponding iminium cations and enamine formation by aerobic oxidation of **1a** in the presence of Au nanoparticle catalysts.

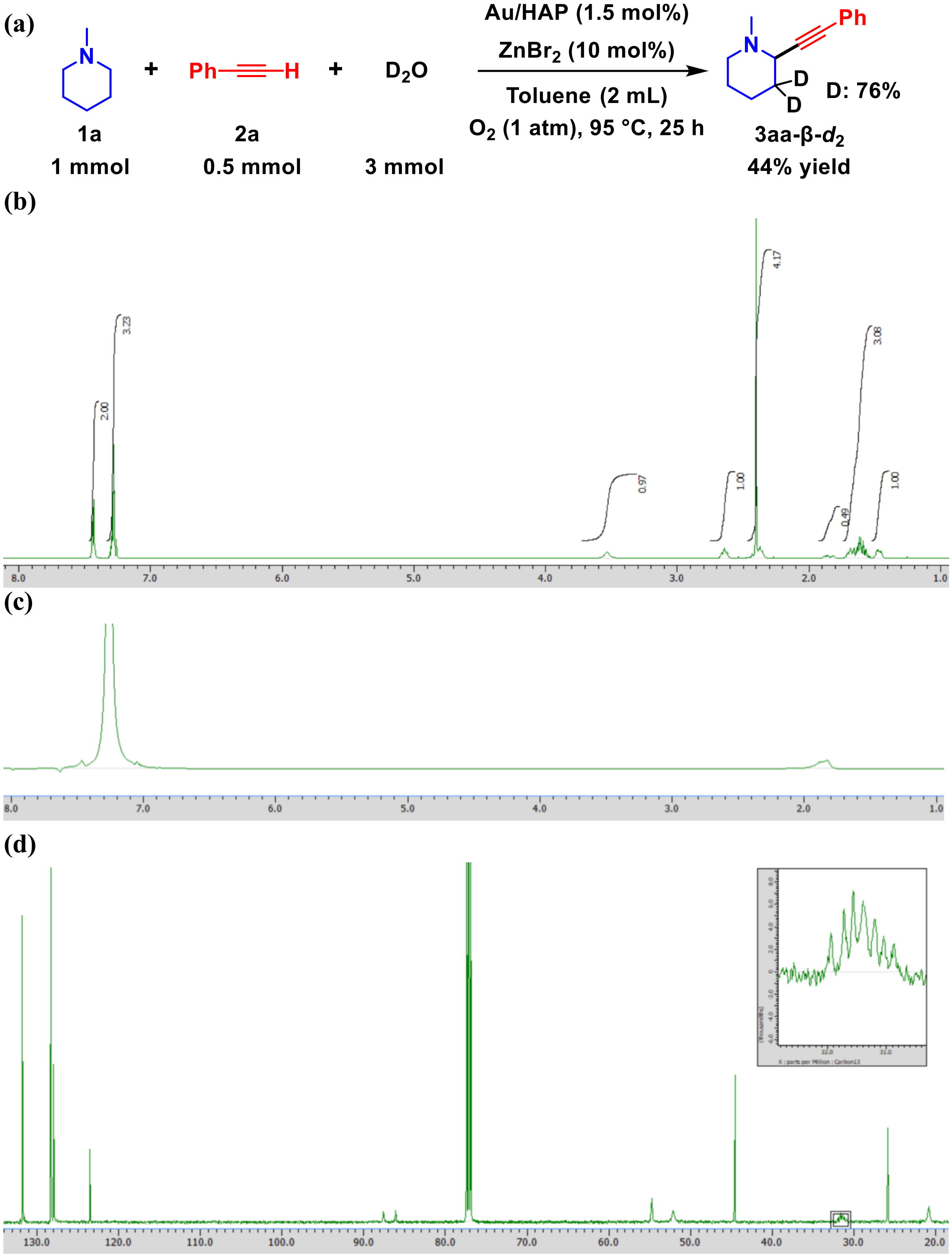

Supplementary Fig. 28 (a) β-Deuterated propargylic amine synthesis. Reaction conditions are indicated in this figure, and the yield was determined by isolation. The deuteration ratio was determined by ^{1}H NMR. (b) ^{1}H NMR, (c) ^{2}H NMR, and (d) ^{13}C NMR spectra of the isolated **3aa-β-*d*$_2$**. recorded at ~25 °C in $CDCl_3$ at 500 MHz, 77 MHz, and 126 MHz, respectively.

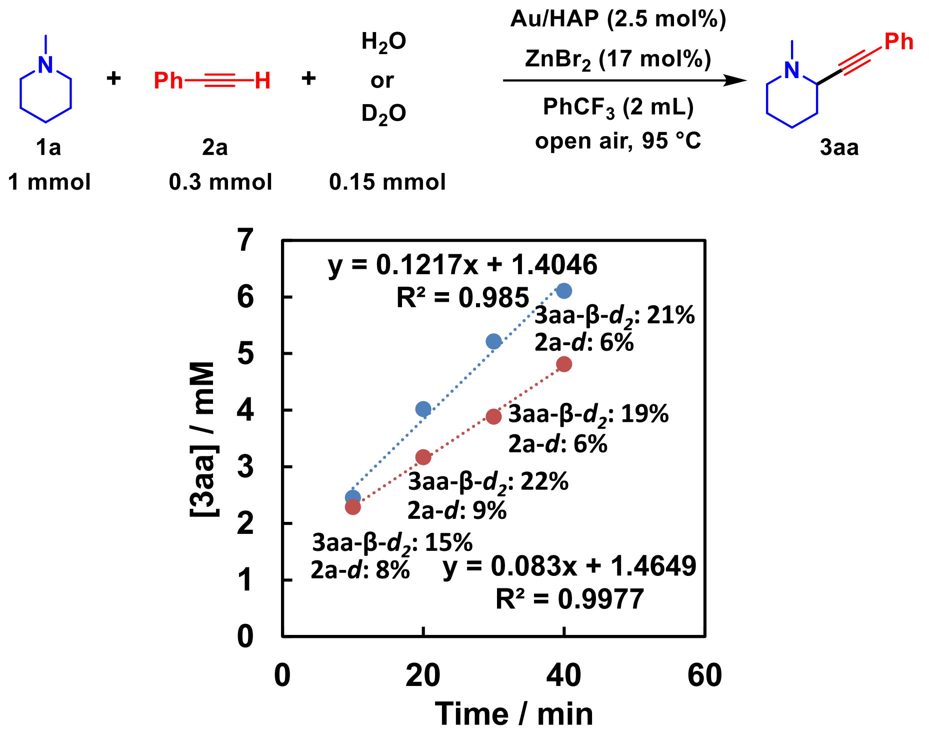

Supplementary Fig. 29 Kinetic isotope effect investigation on α-alkynylation of **1a** in the presence of Au/HAP and $ZnBr_2$ using **2a** as an alkyne substrate with H_2O or D_2O, respectively. Reaction conditions are indicated in this figure, and the yields were determined by GC using biphenyl as internal standard. GC-MS spectra were recorded in scan or selected ion monitoring mode, and deuteration ratios of each sampling were approximately estimated from the relative intensity of m/z = 103 or 200.

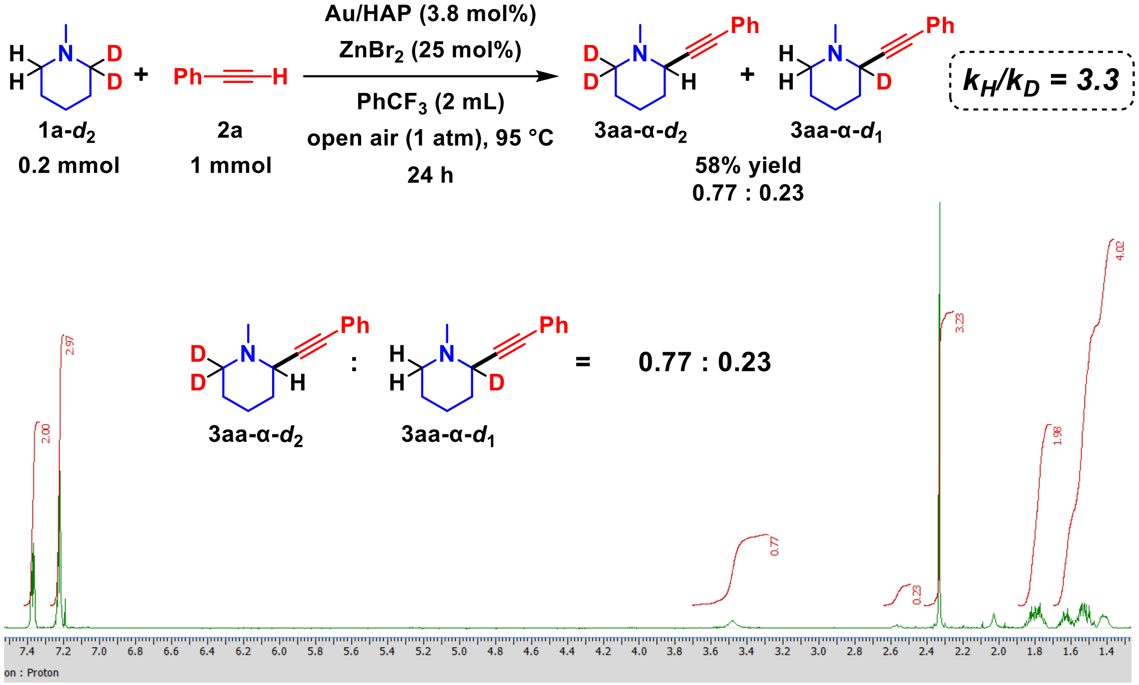

Supplementary Fig. 30 Kinetic isotope effect on α-alkynylation with **2a** in the presence of Au/HAP and $ZnBr_2$ using **1a-*d*$_2$** as an amine substrate. Reaction conditions are indicated in the figure, and the isolated yield of **3aa** is shown (GC yield determined using biphenyl as internal standard is also 58%). The shown kinetic isotope effect was calculated from the ^1H NMR spectrum of isolated **3aa** at the lower side, recorded at ~25 °C in $CDCl_3$ at 500 MHz.

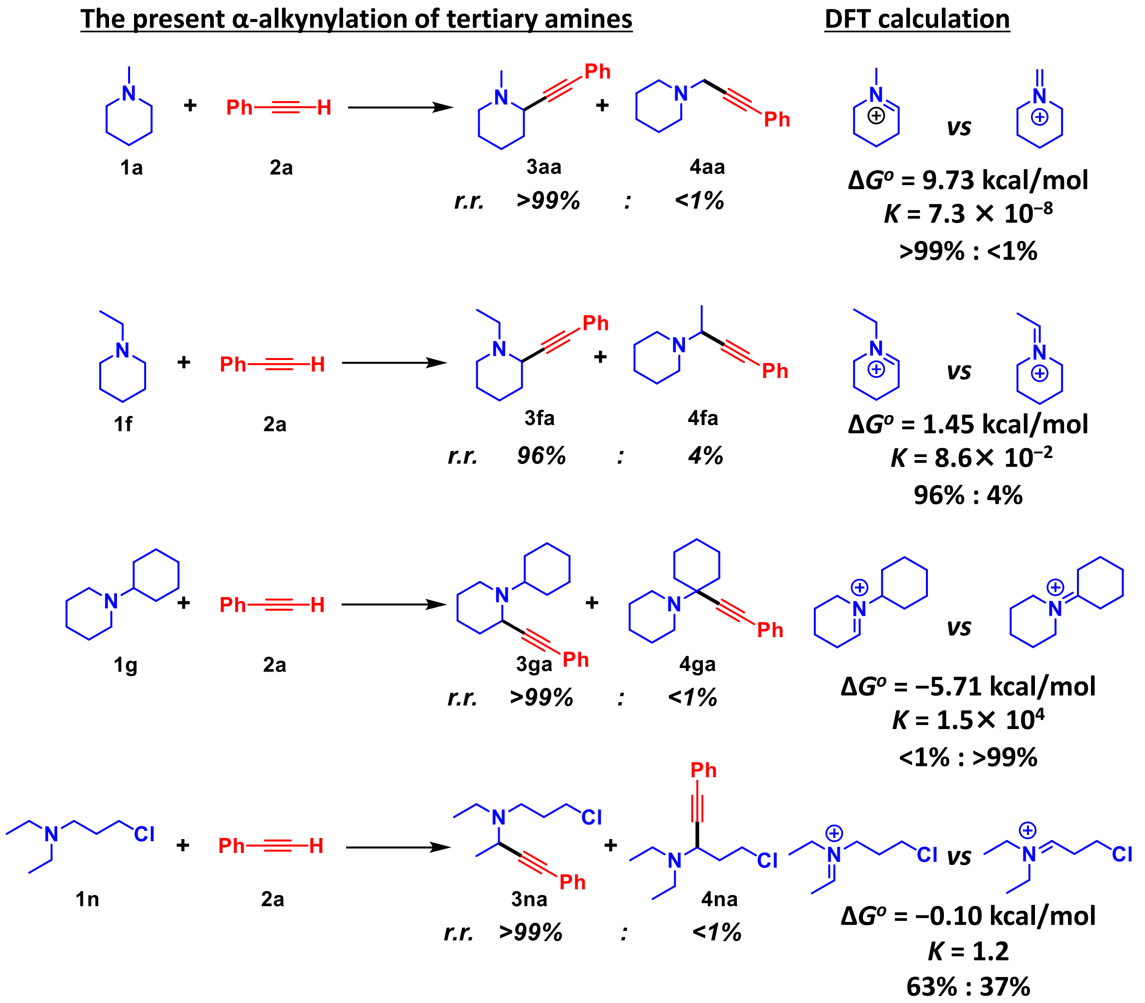

Supplementary Fig. 31 Comparison of regioselectivity to Au/HAP and $ZnBr_2$-catalyzed α-alkynylation of several selected tertiary amines (shown in Table 3) with Gibbs free energy difference given by DFT calculation between iminium cations derived from dehydrogenation at the cyclic methylene positions and the counterparts. Equilibrium constants (K) were calculated from $\Delta G^o = -RT\ln K$ (T = 298.15 K). The number of α-C–H bonds of amines was taken into consideration on the shown ratios of iminium cations based on DFT calculation.

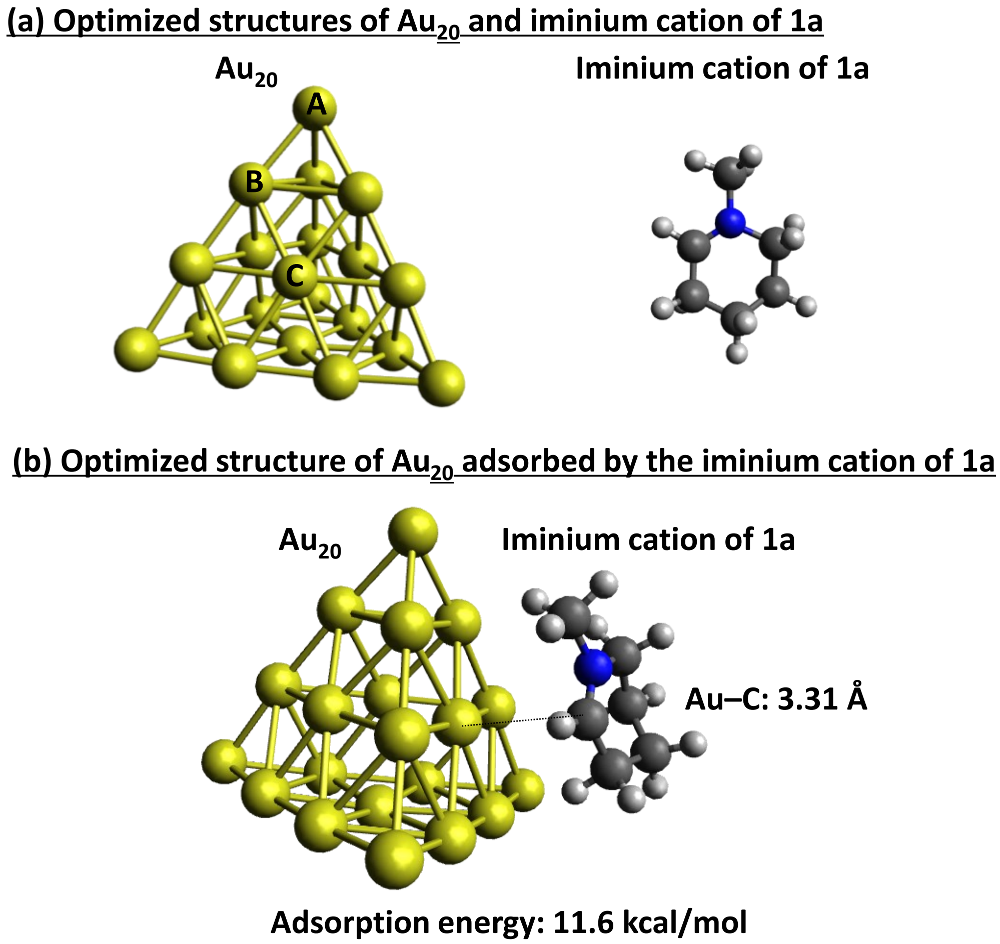

Supplementary Fig. 32 Optimized structures of (a) Au_{20} cluster with no charge, the corresponding iminium cation of **1a**, and (b) Au_{20} cluster adsorbed by the corresponding iminium cation of **1a** based on DFT calculation. The Au_{20} cluster model was constructed by referring to our previous report[S28]. Although three types of initial adsorbed structures were investigated (adsorption site A, B, or C of Au_{20} cluster shown in this figure (a)), all the structures converged to the adsorbed structure at the site C as shown in this figure (b). The adsorption energy was calculated based on Gibbs energies of Au_{20} cluster, the corresponding iminium cation of **1a**, and Au_{20} cluster adsorbed by the corresponding iminium cation of **1a** in this figure.

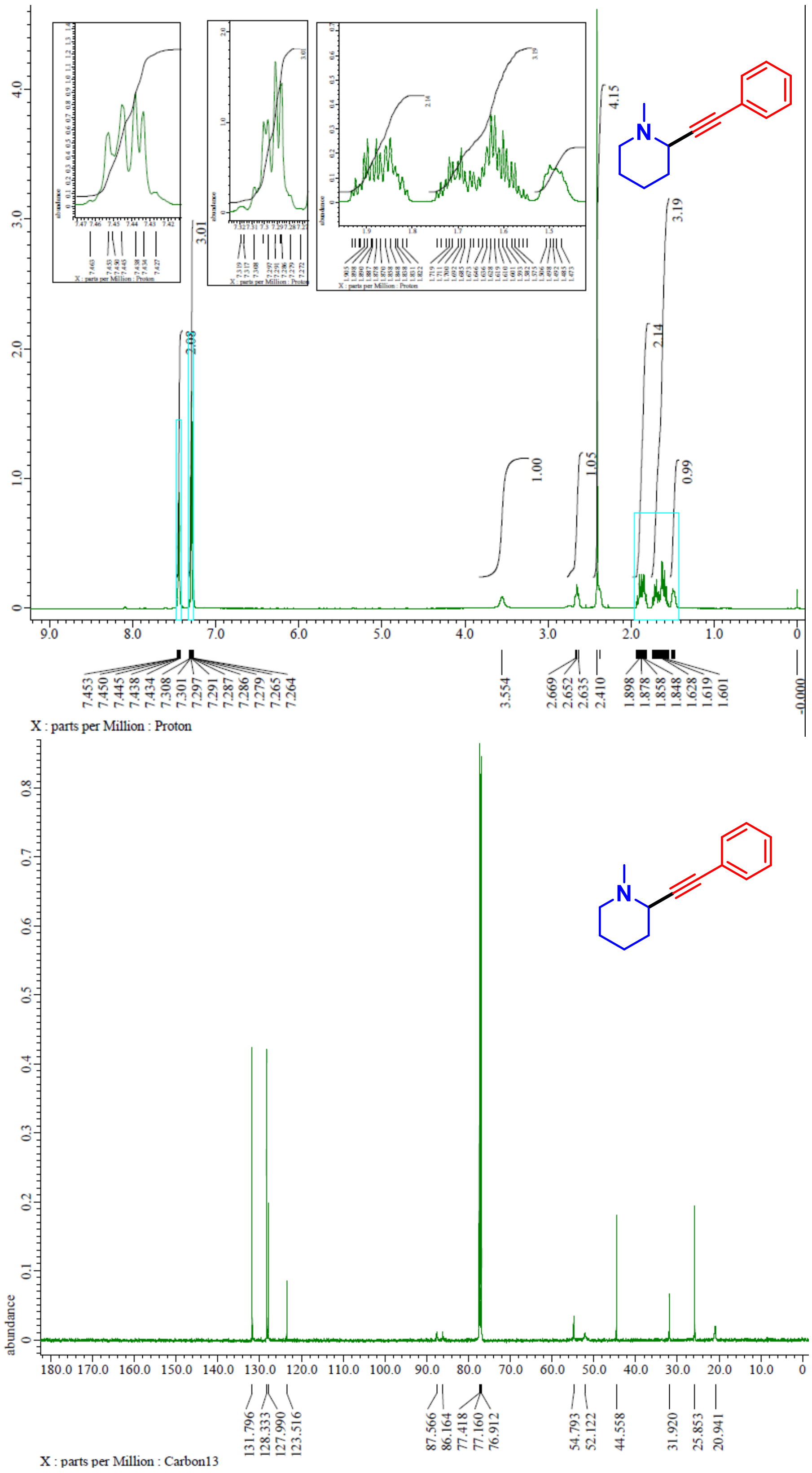

Supplementary Fig. 33. ^{1}H NMR and ^{13}C NMR of **3aa**, recorded at ~25 °C in $CDCl_3$ at 500 MHz and 126 MHz, respectively.

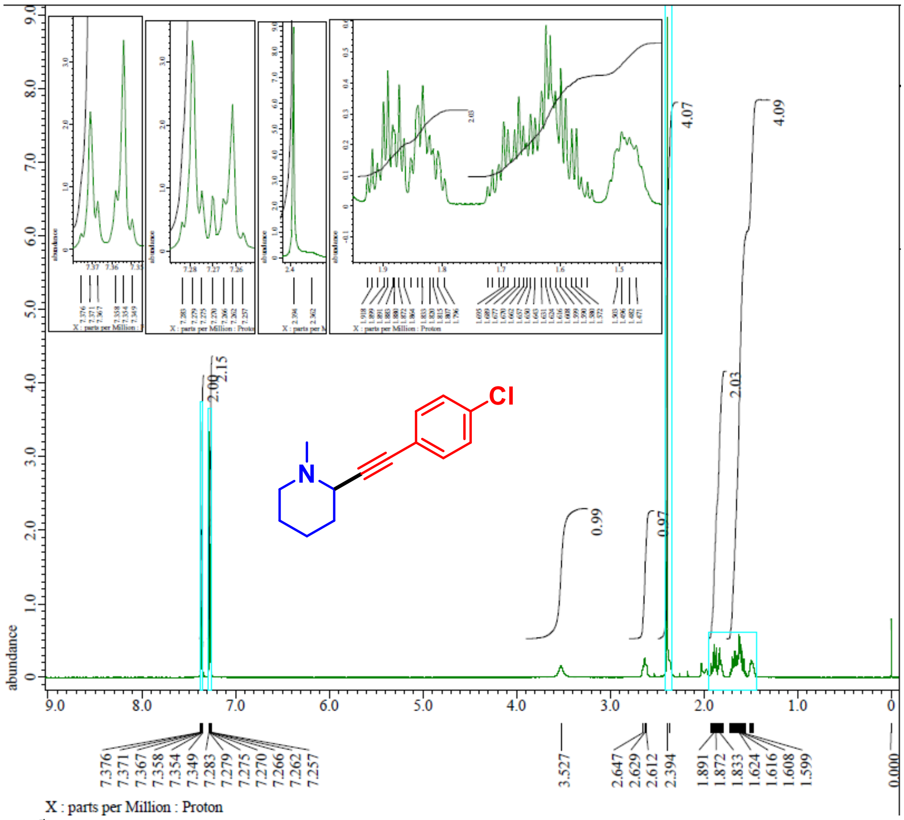

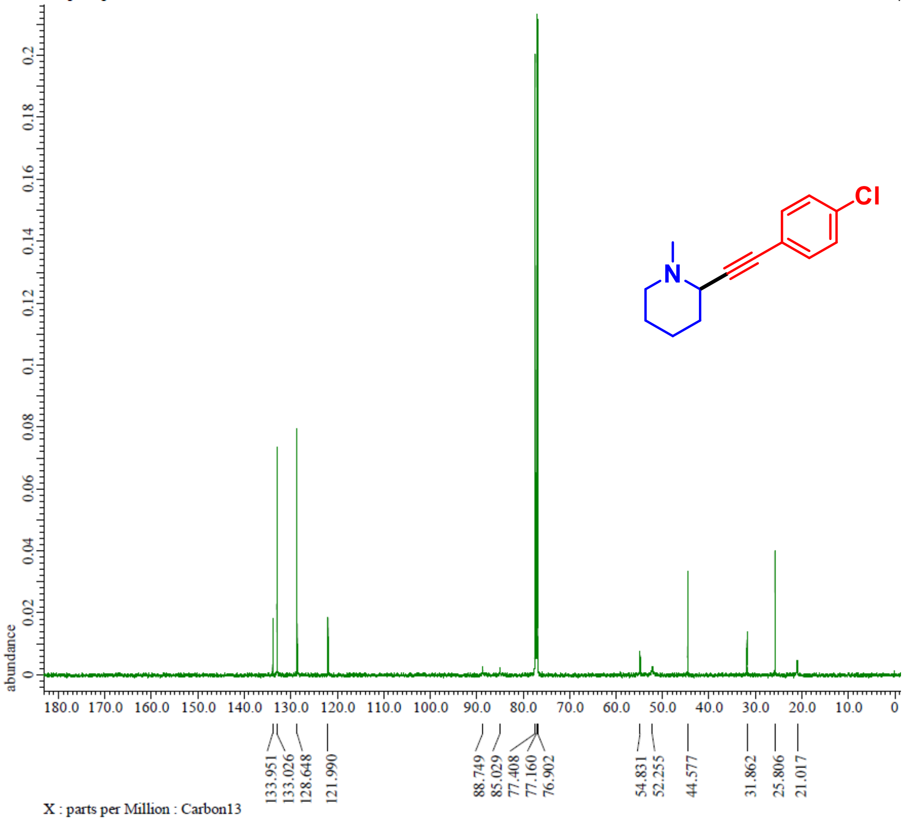

Supplementary Fig. 34. ^{1}H NMR and ^{13}C NMR of **3ab**, recorded at ~25 °C in $CDCl_3$ at 500 MHz and 126 MHz, respectively.

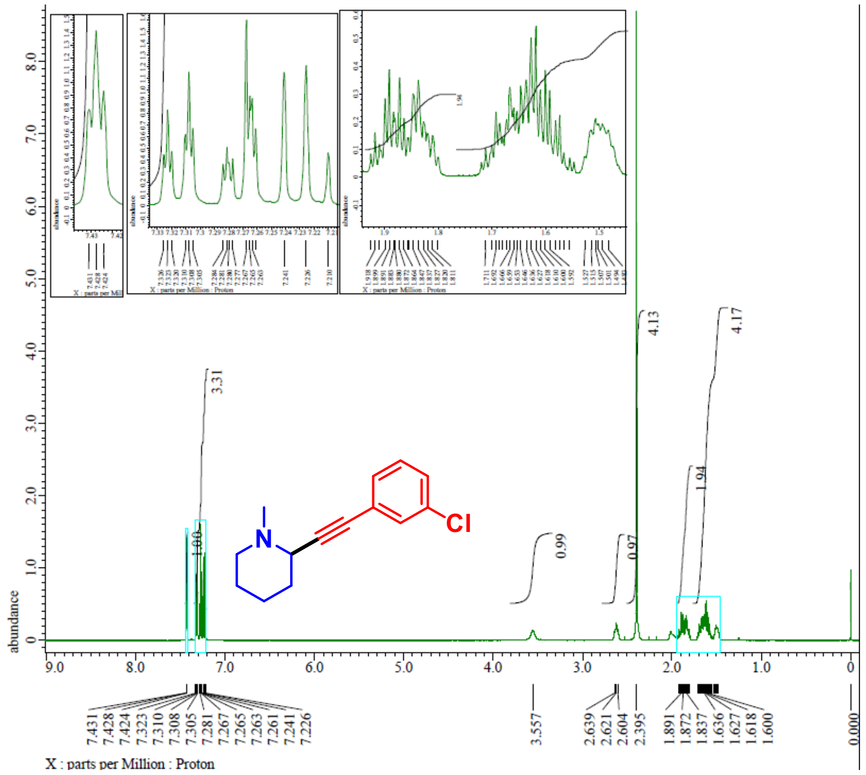

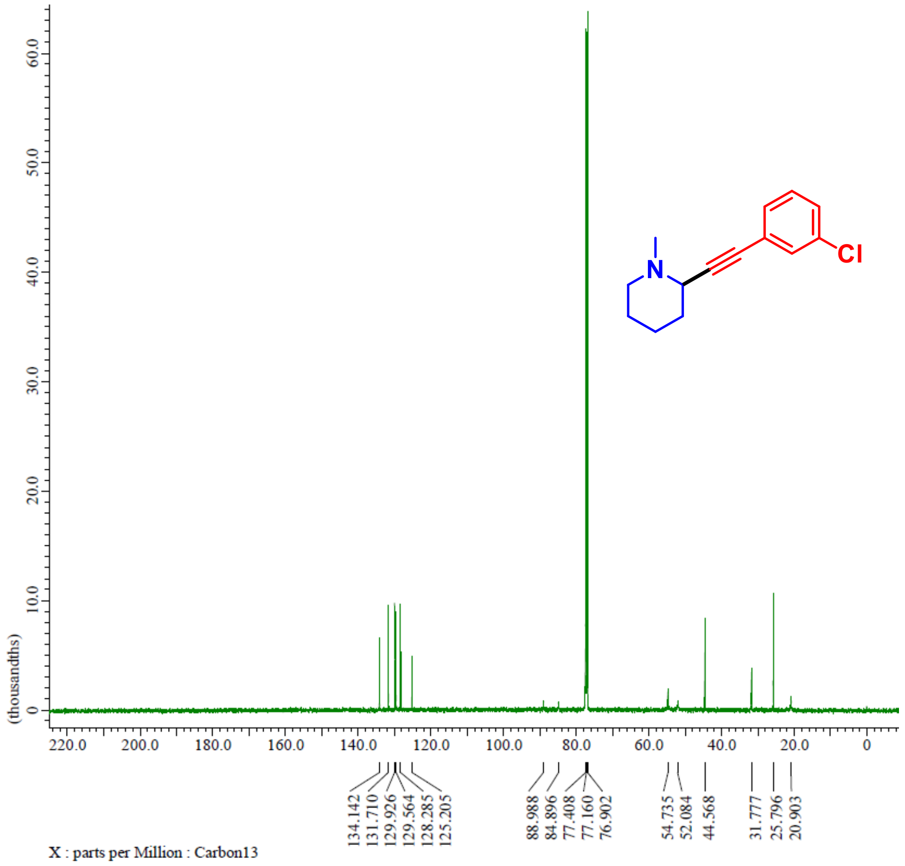

Supplementary Fig. 35. ^{1}H NMR and ^{13}C NMR of **3ac**, recorded at ~25 °C in $CDCl_3$ at 500 MHz and 126 MHz, respectively.

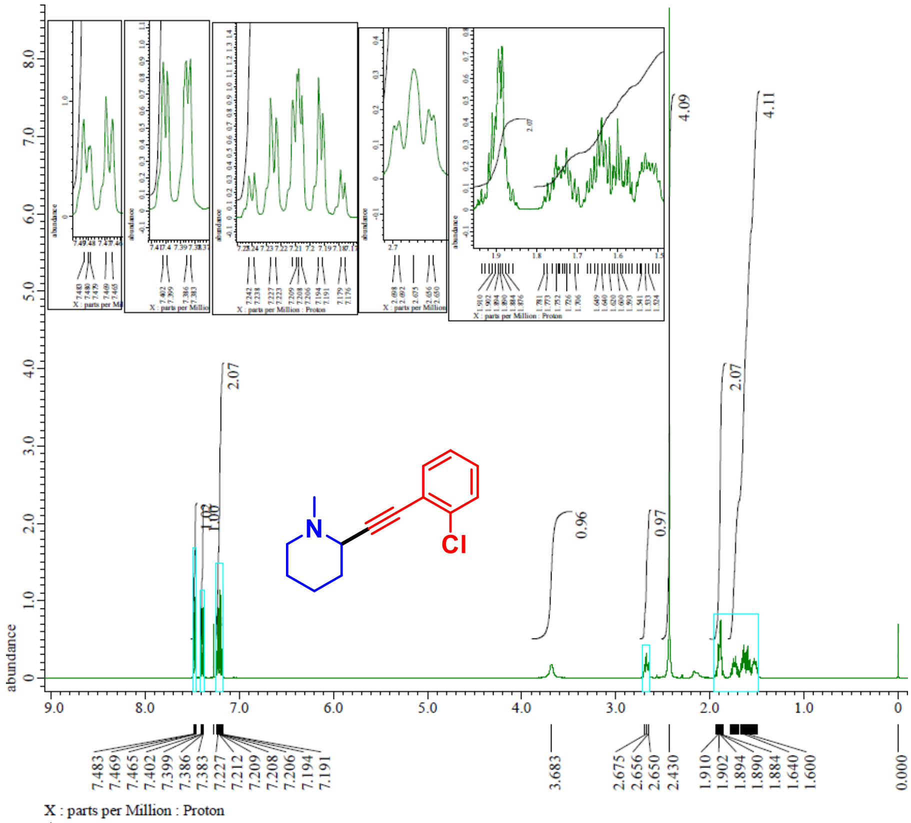

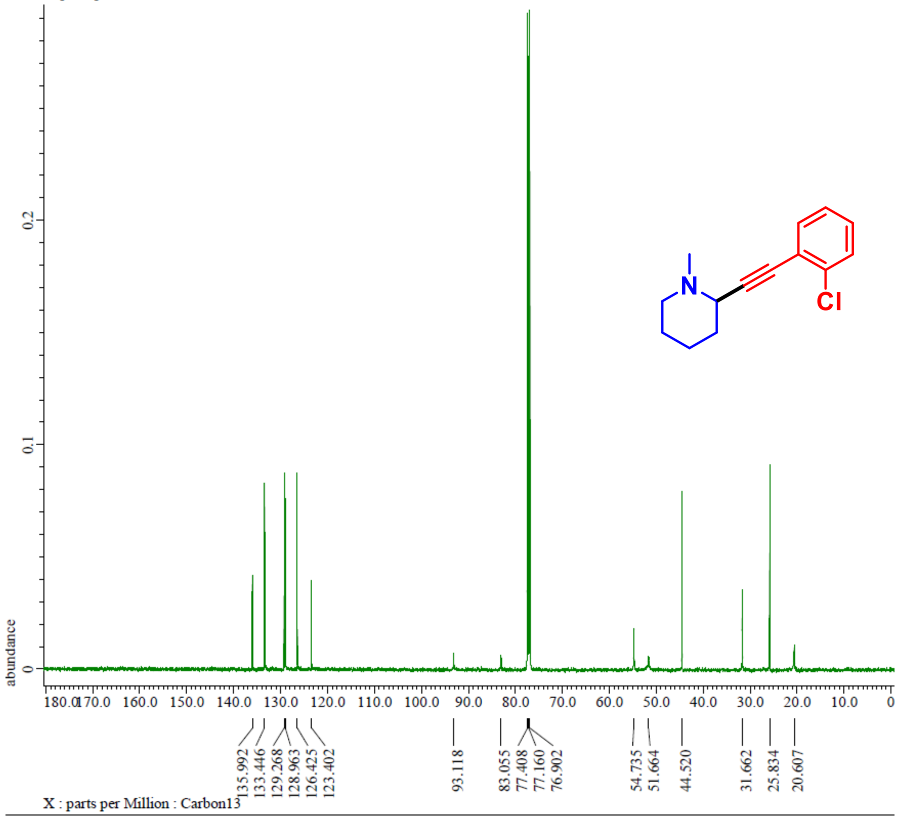

Supplementary Fig. 36. ^{1}H NMR and ^{13}C NMR of **3ad**, recorded at ~25 °C in $CDCl_3$ at 500 MHz and 126 MHz, respectively.

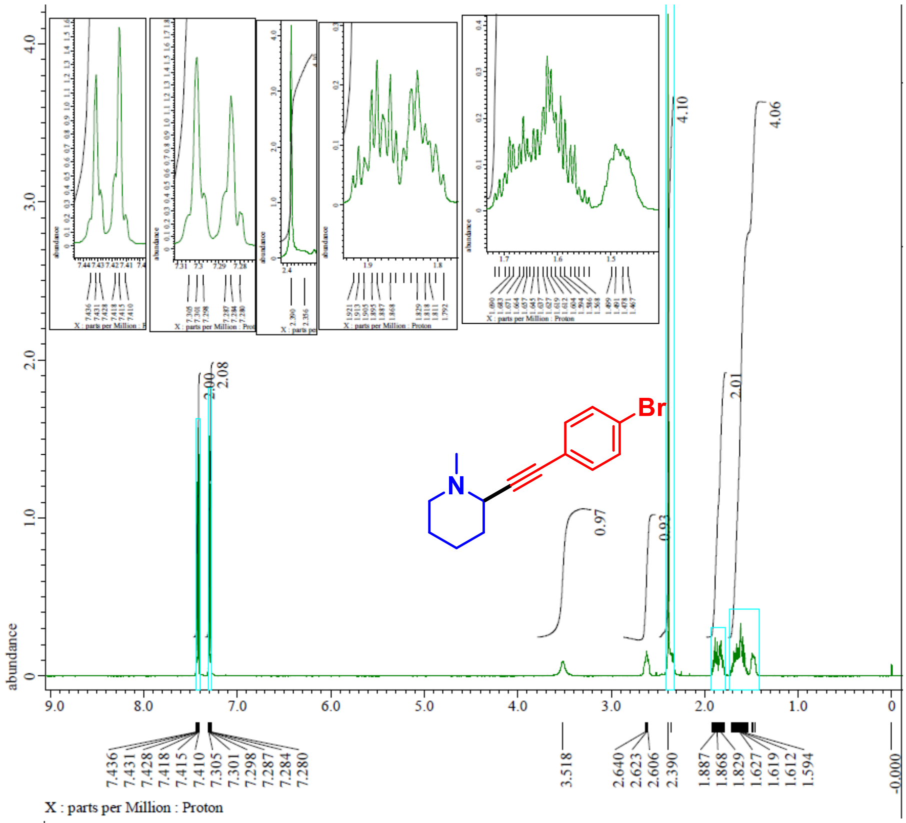

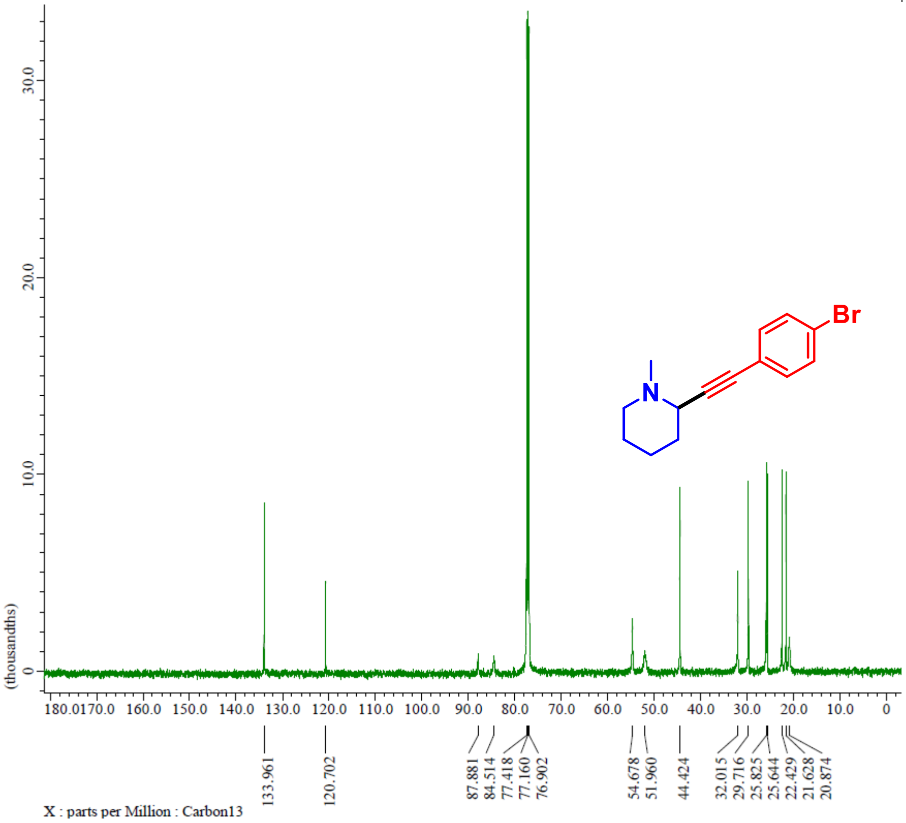

Supplementary Fig. 37. ^{1}H NMR and ^{13}C NMR of **3ae**, recorded at ~25 °C in $CDCl_3$ at 500 MHz and 126 MHz, respectively.

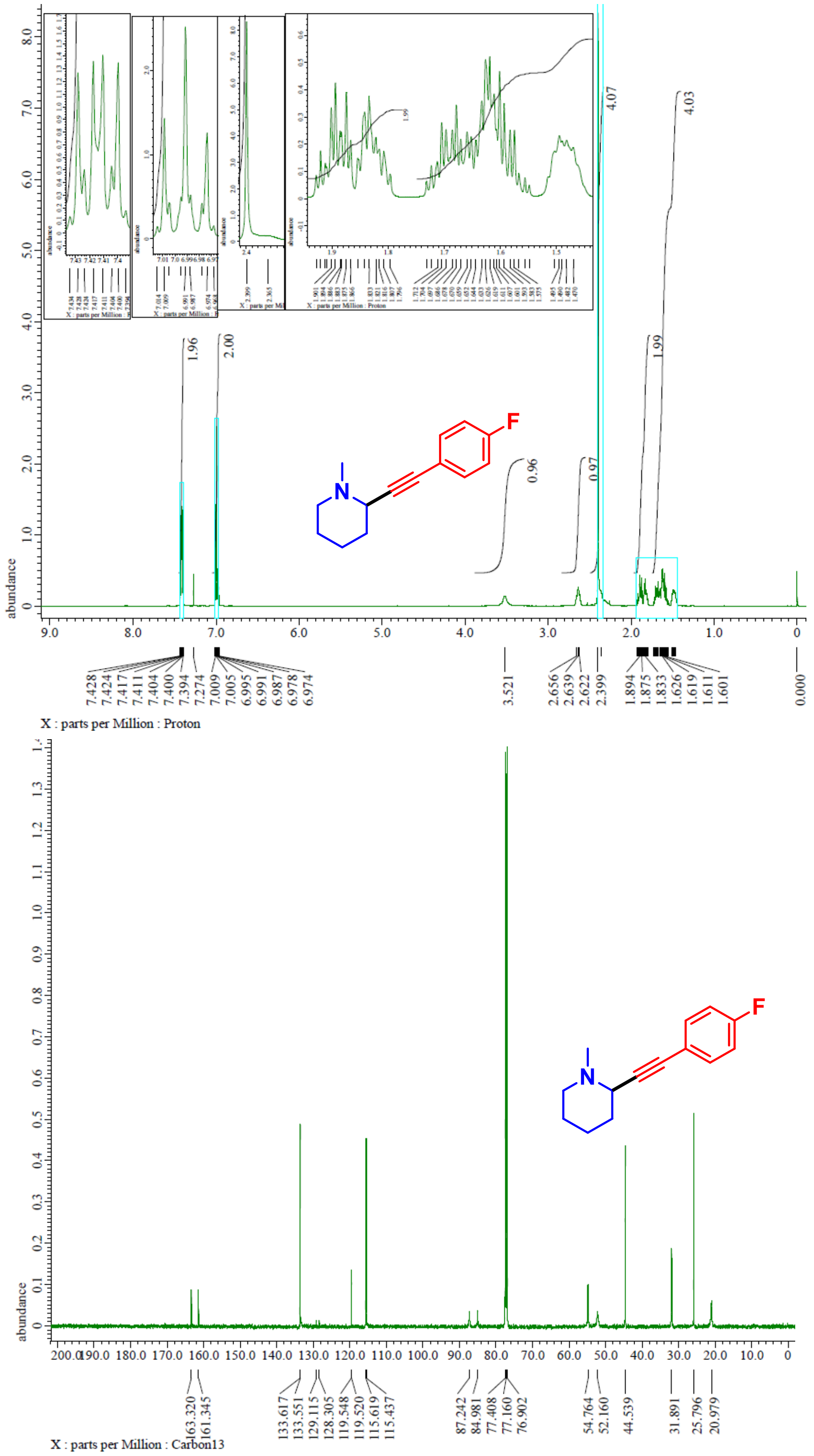

Supplementary Fig. 38. ^{1}H NMR and ^{13}C NMR of **3af**, recorded at ~25 °C in $CDCl_3$ at 500 MHz and 126 MHz, respectively.

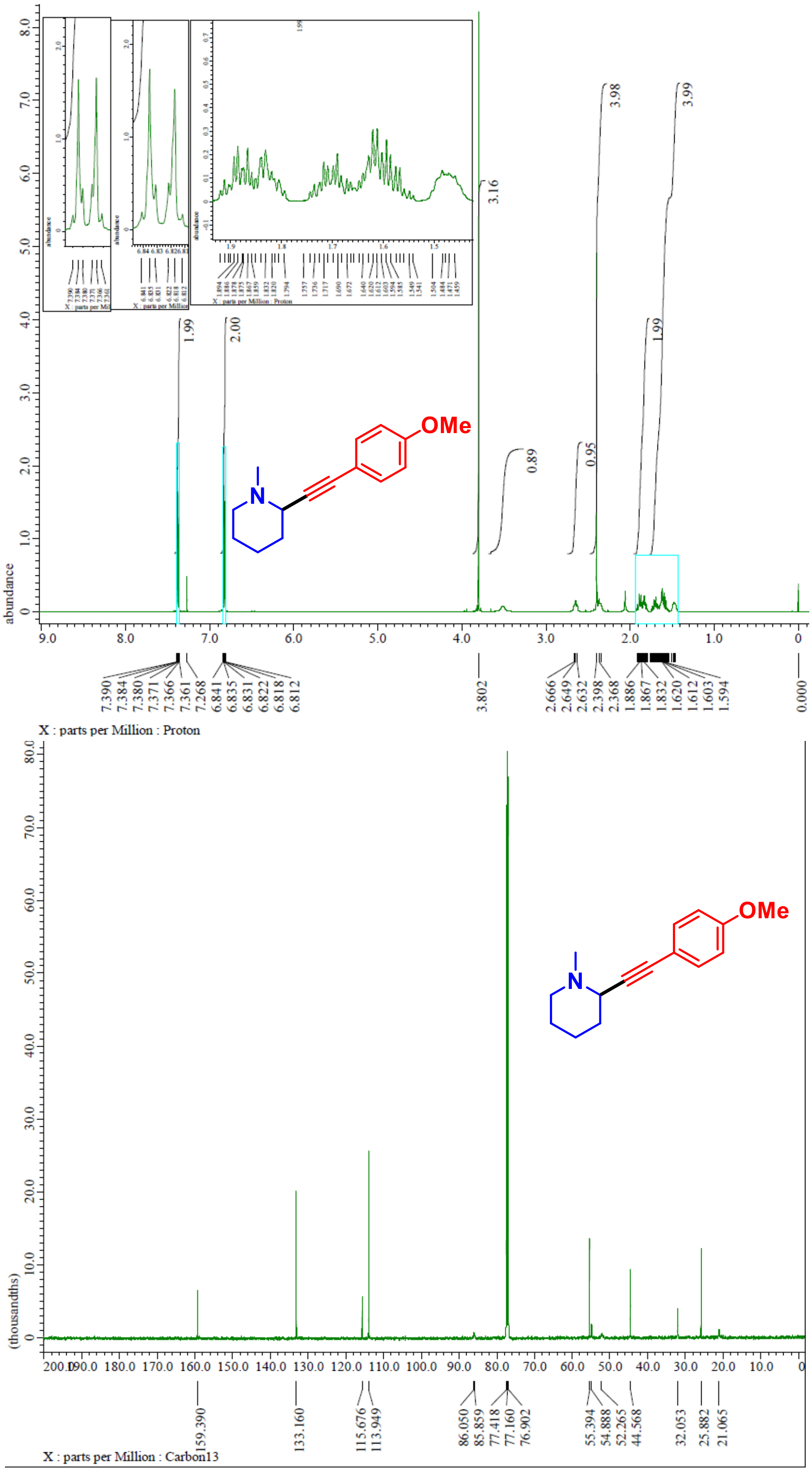

Supplementary Fig. 39. ^{1}H NMR and ^{13}C NMR of **3ag**, recorded at ~25 °C in $CDCl_3$ at 500 MHz and 126 MHz, respectively.

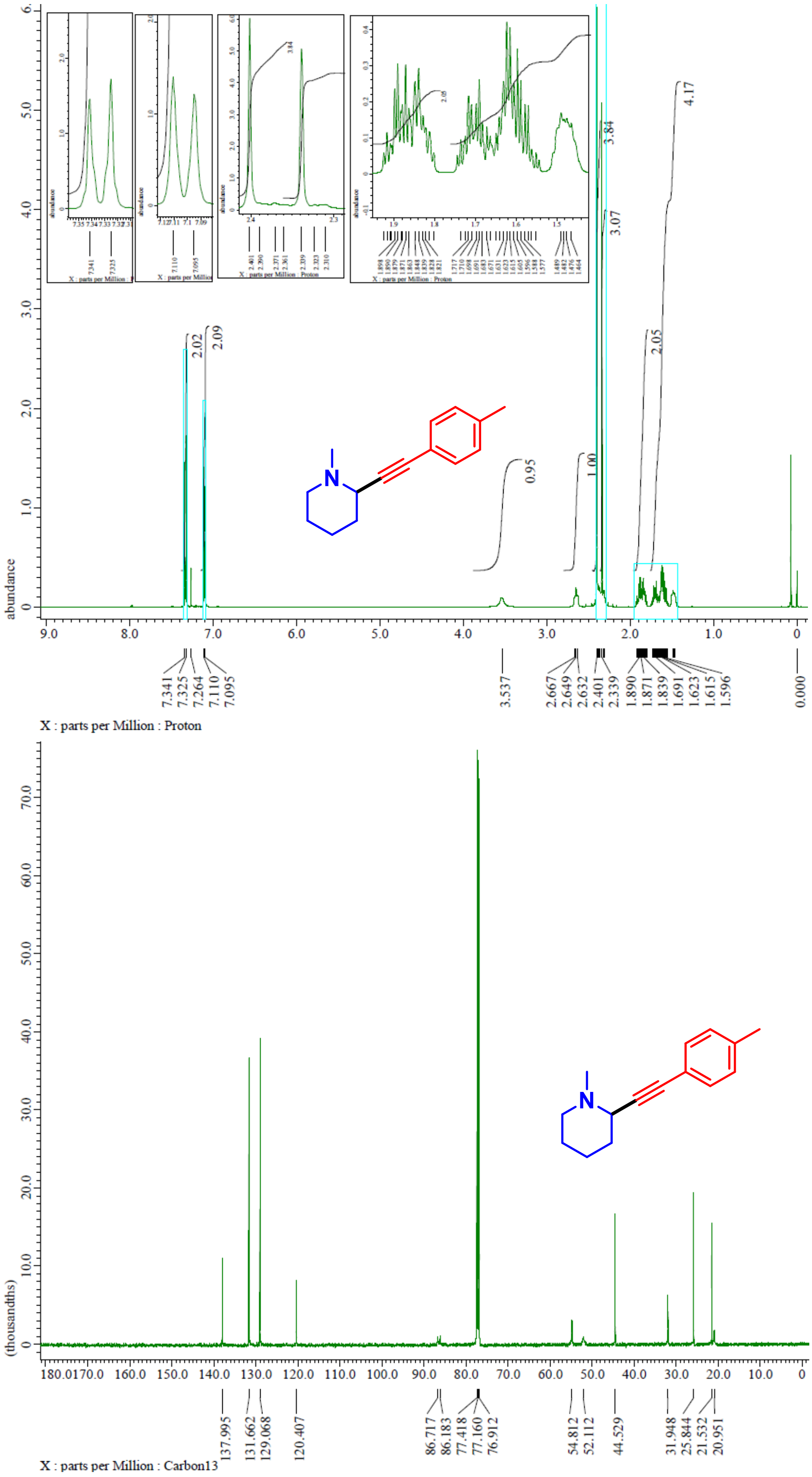

Supplementary Fig. 40. ^1H NMR and ^{13}C NMR of **3ah**, recorded at ~25 °C in $CDCl_3$ at 500 MHz and 126 MHz, respectively.

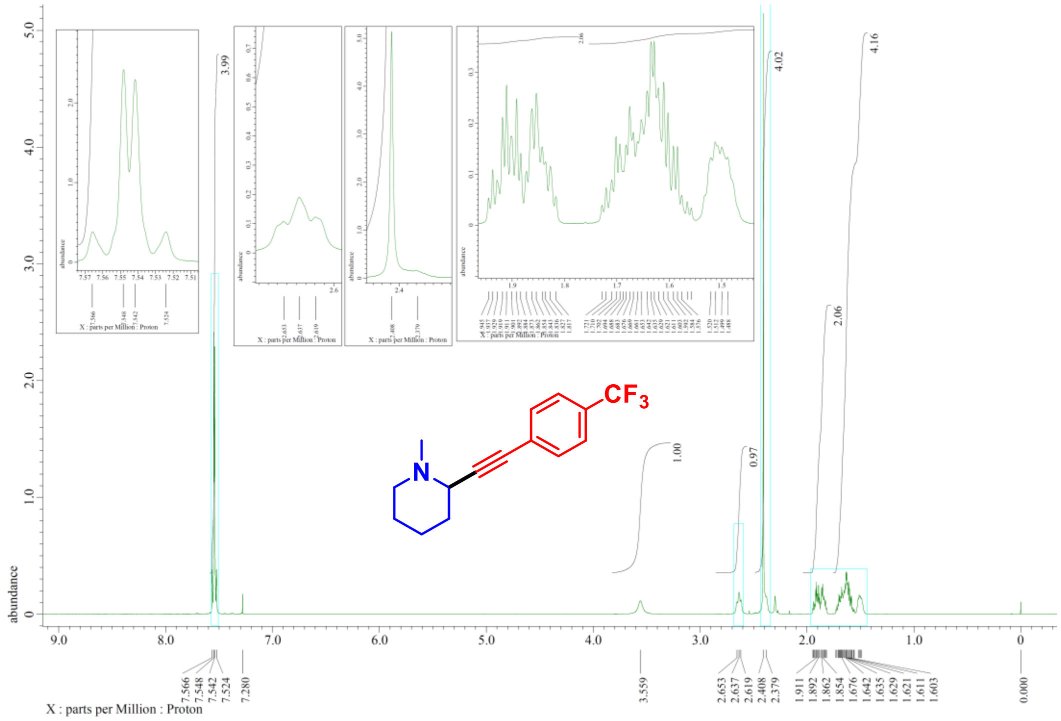

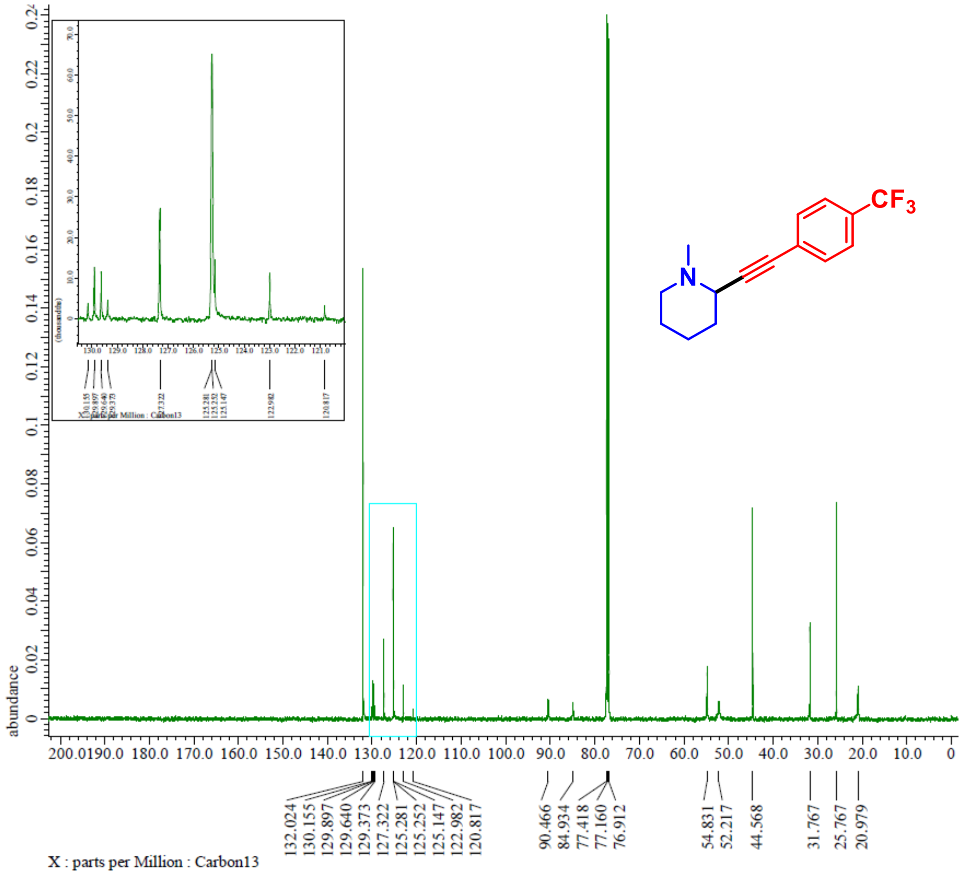

Supplementary Fig. 41. ^{1}H NMR and ^{13}C NMR of **3ai**, recorded at ~25 °C in $CDCl_3$ at 500 MHz and 126 MHz, respectively.

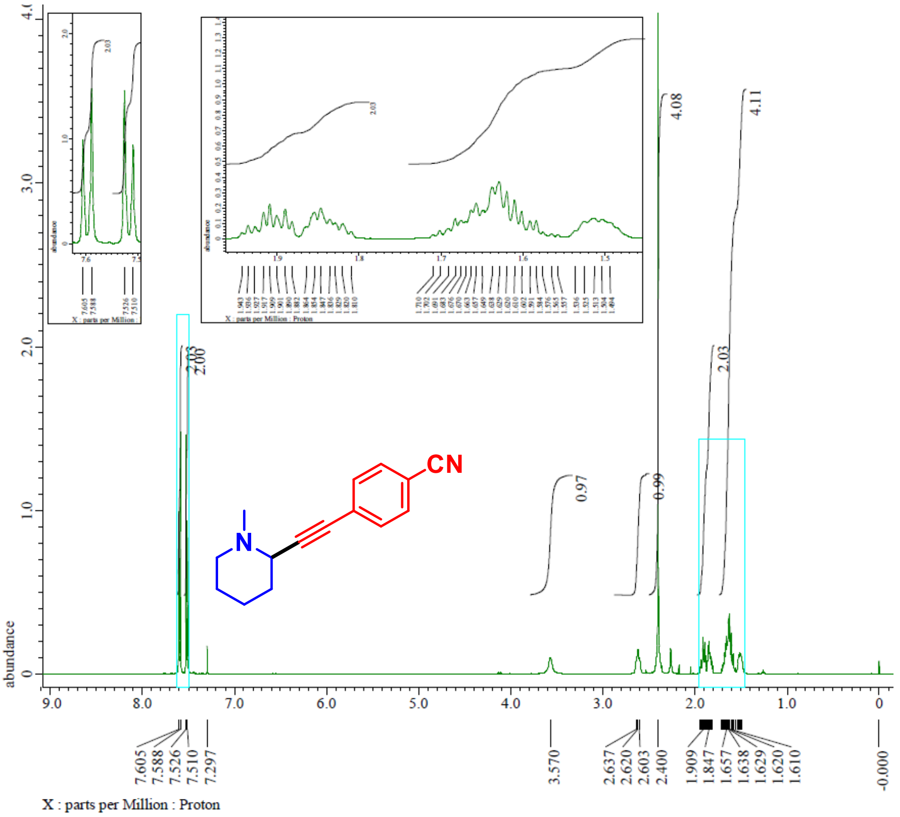

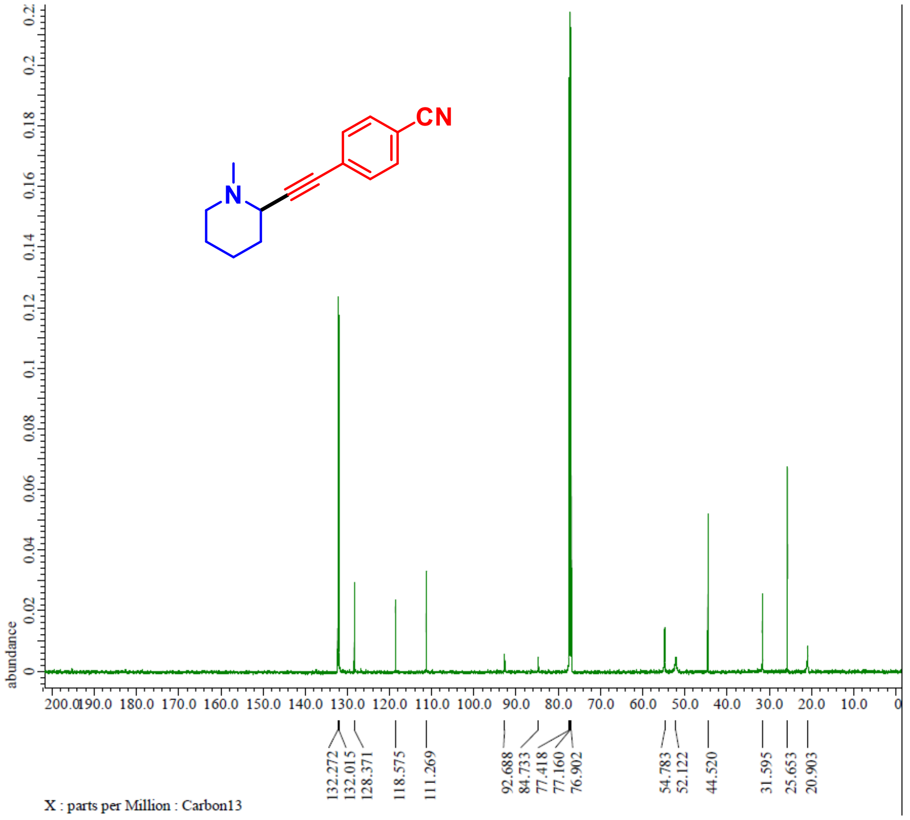

Supplementary Fig. 42. ^{1}H NMR and ^{13}C NMR of **3aj**, recorded at ~25 °C in $CDCl_3$ at 500 MHz and 126 MHz, respectively.

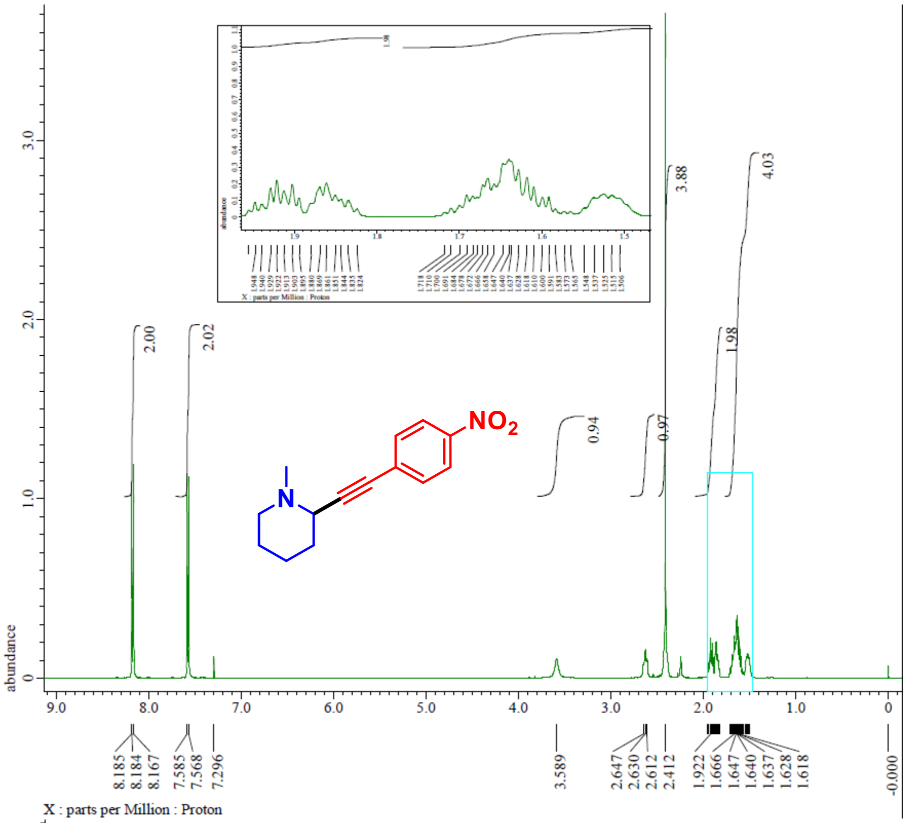

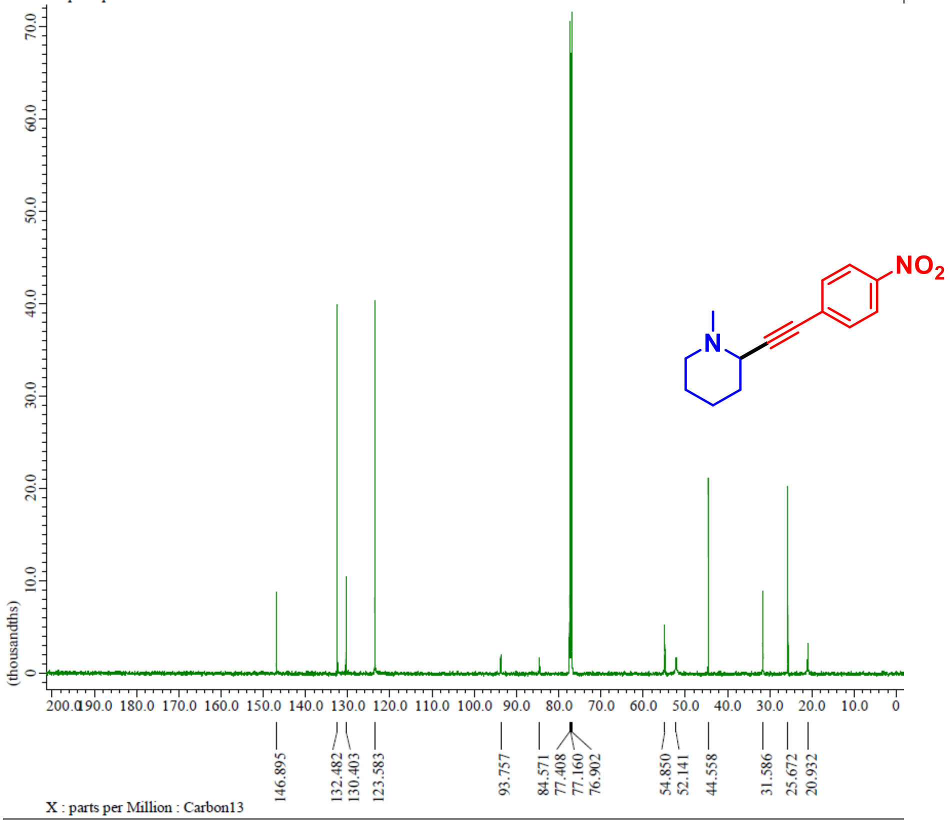

Supplementary Fig. 43. ^{1}H NMR and ^{13}C NMR of **3ak**, recorded at ~25 °C in $CDCl_3$ at 500 MHz and 126 MHz, respectively.

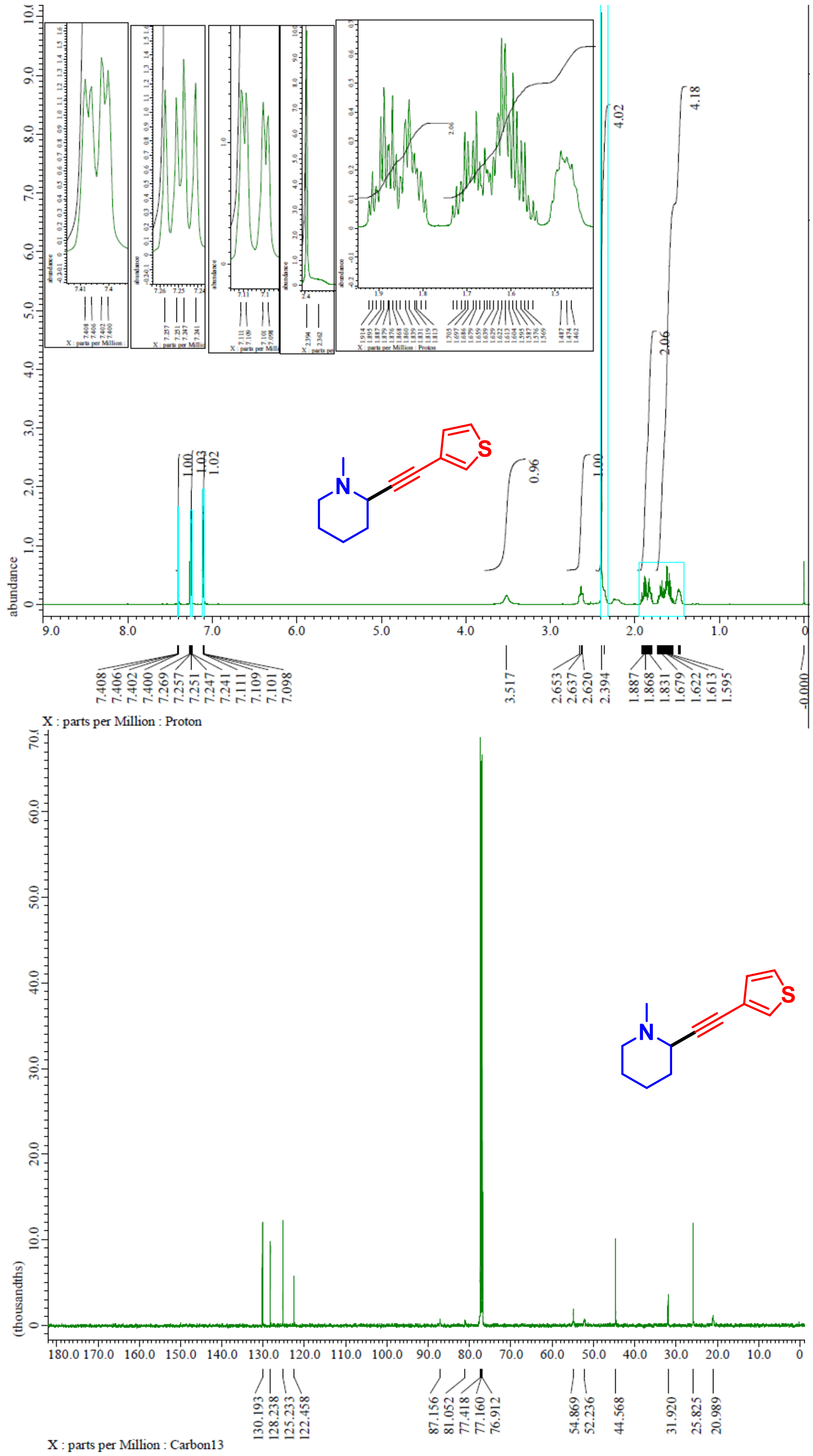

Supplementary Fig. 44. ^{1}H NMR and ^{13}C NMR of **3al**, recorded at ~25 °C in $CDCl_3$ at 500 MHz and 126 MHz, respectively.

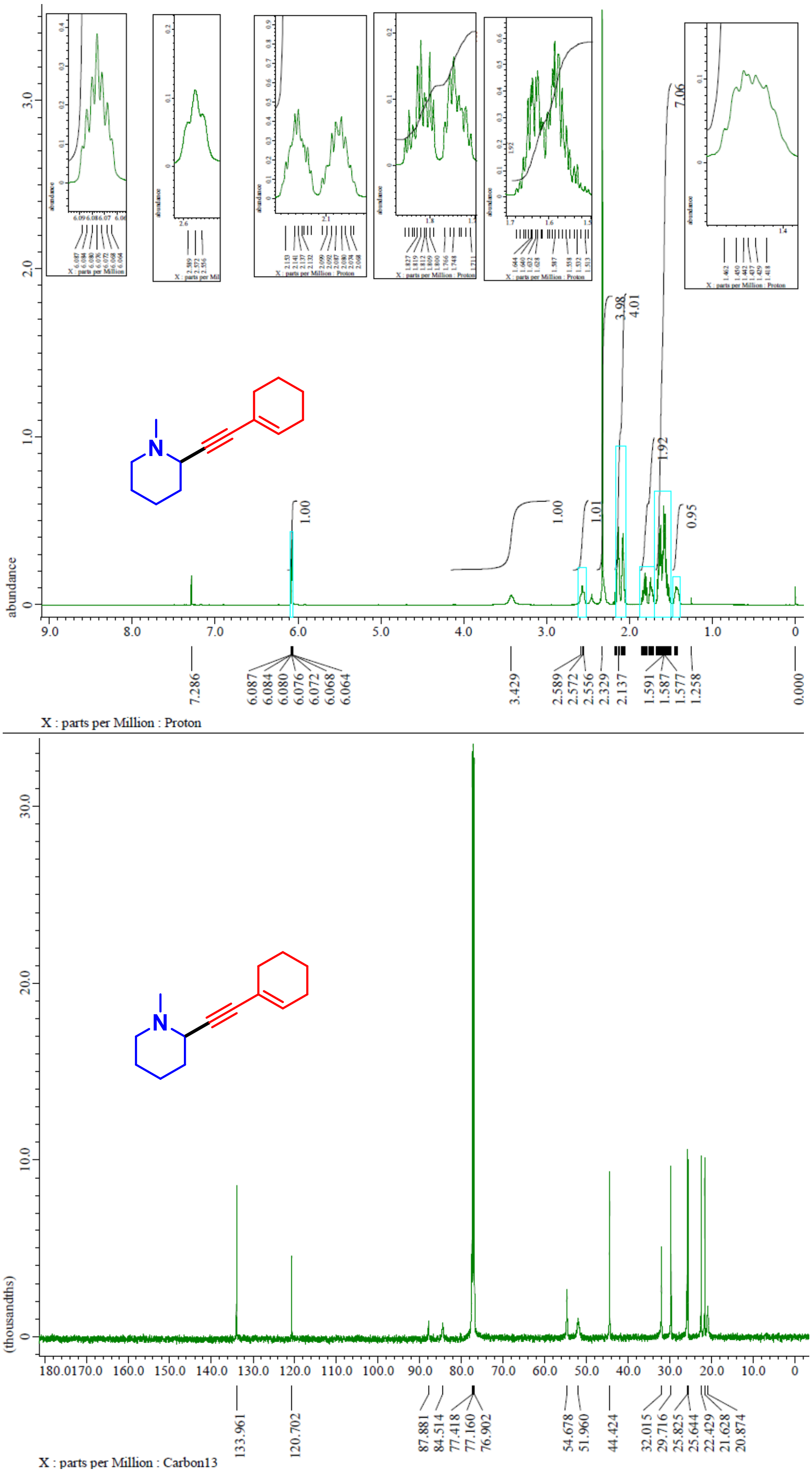

Supplementary Fig. 45. ^{1}H NMR and ^{13}C NMR of **3am**, recorded at ~25 °C in $CDCl_3$ at 500 MHz and 126 MHz, respectively.

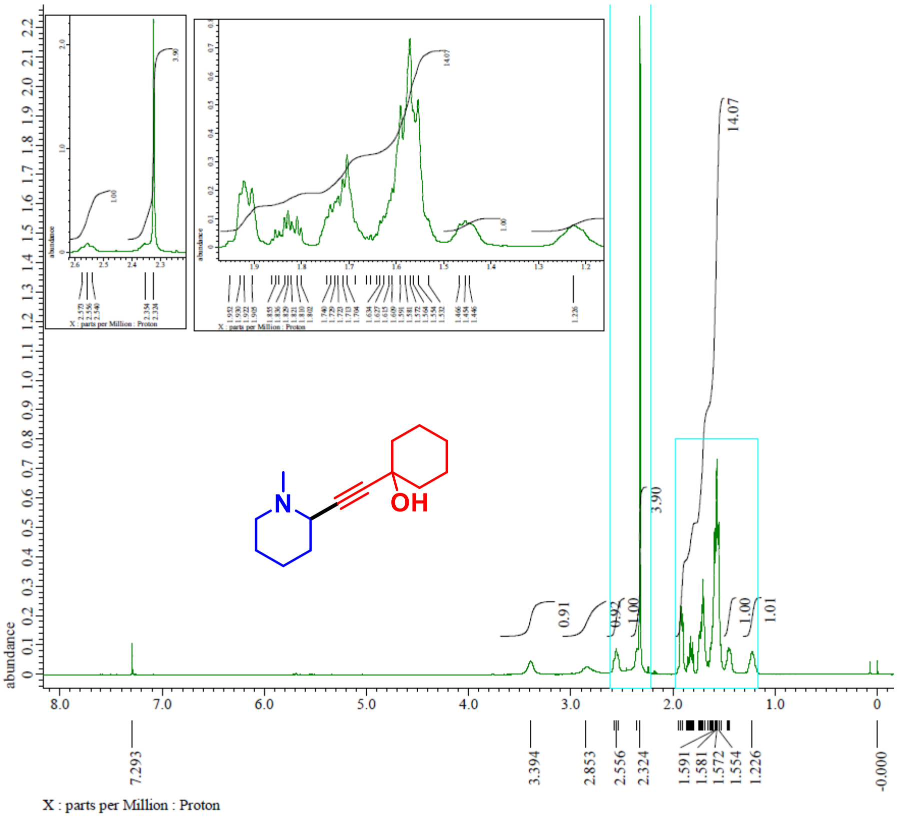

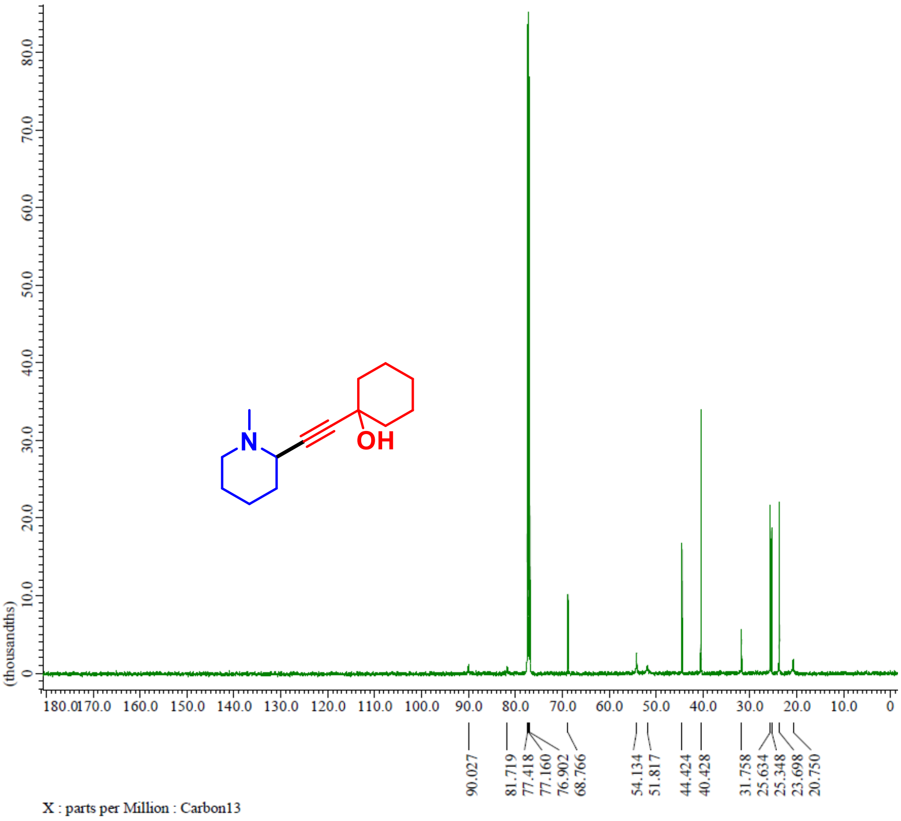

Supplementary Fig. 46. ^{1}H NMR and ^{13}C NMR of **3an**, recorded at ~25 °C in $CDCl_3$ at 500 MHz and 126 MHz, respectively.

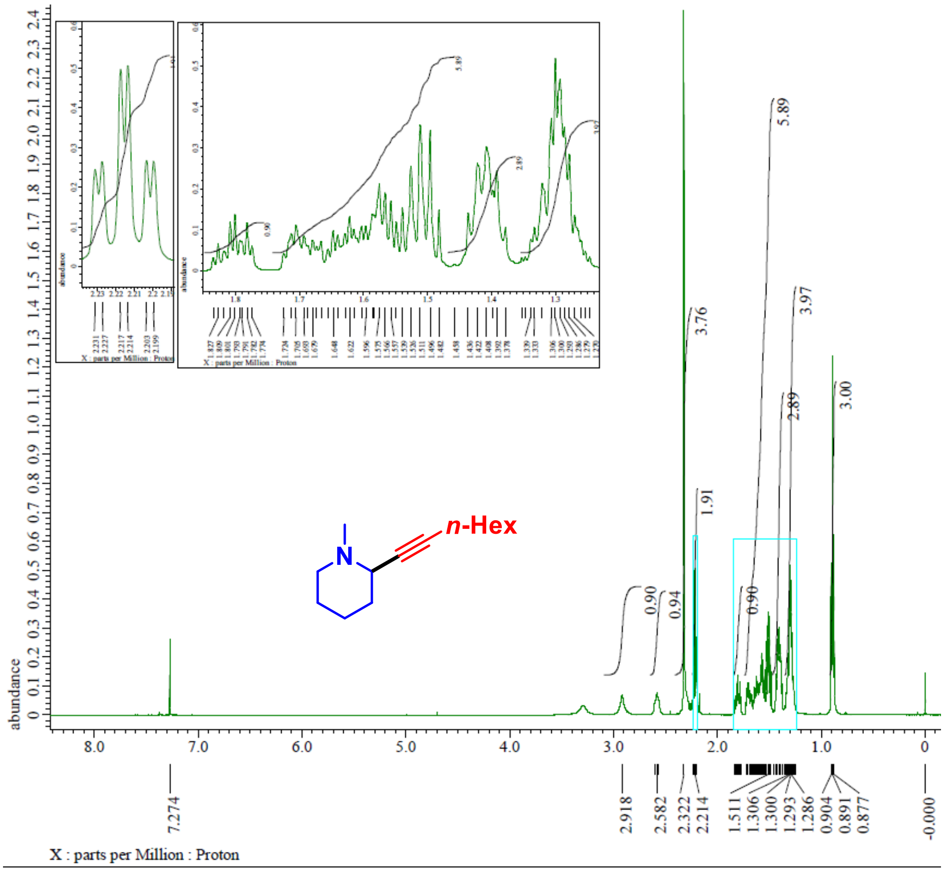

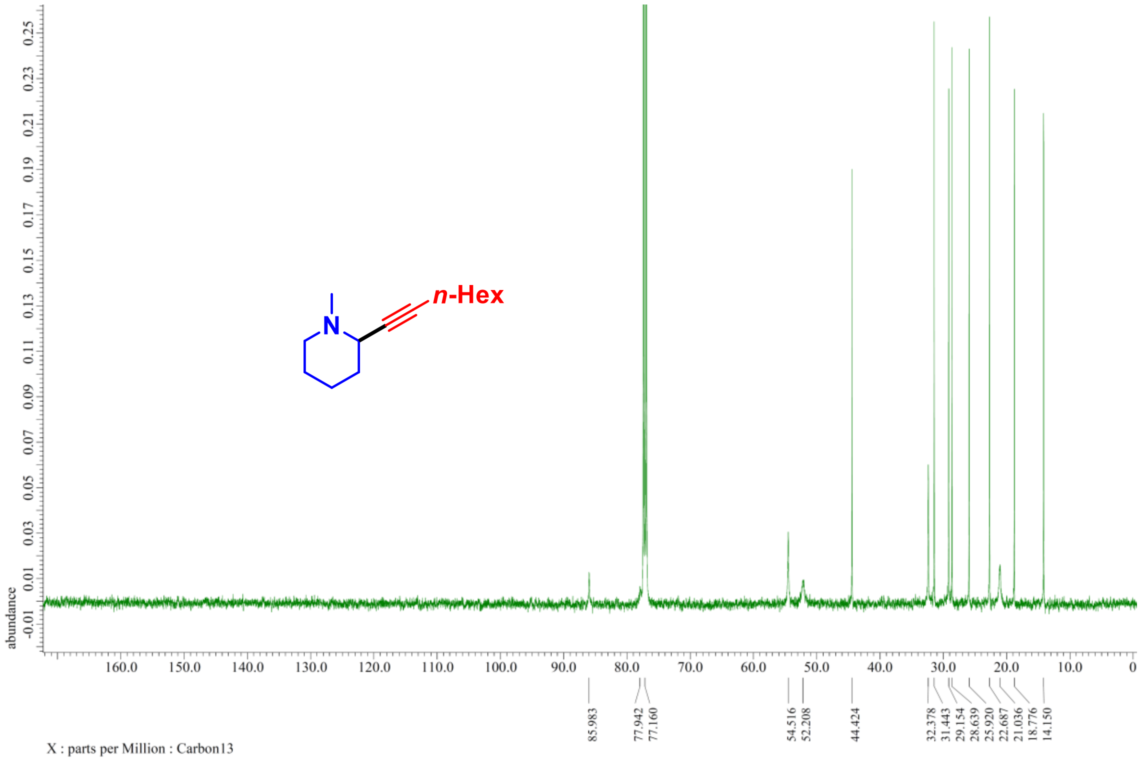

Supplementary Fig. 47. ^{1}H NMR and ^{13}C NMR of **3ao**, recorded at ~25 °C in $CDCl_3$ at 500 MHz and 126 MHz, respectively.

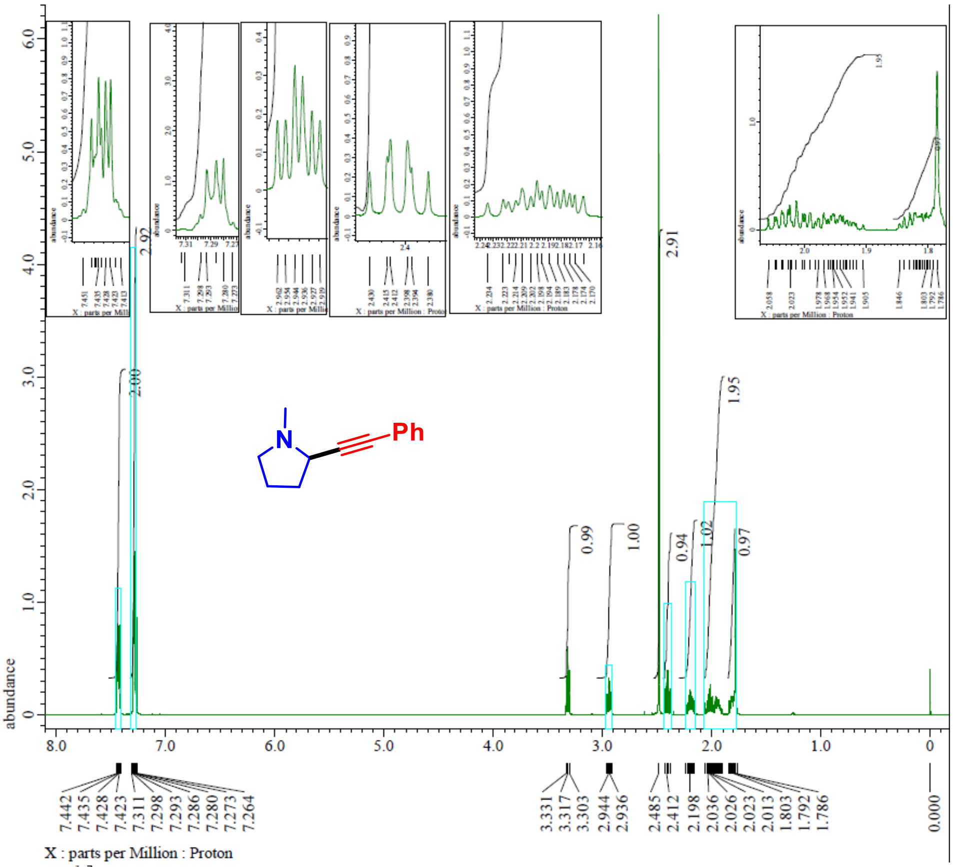

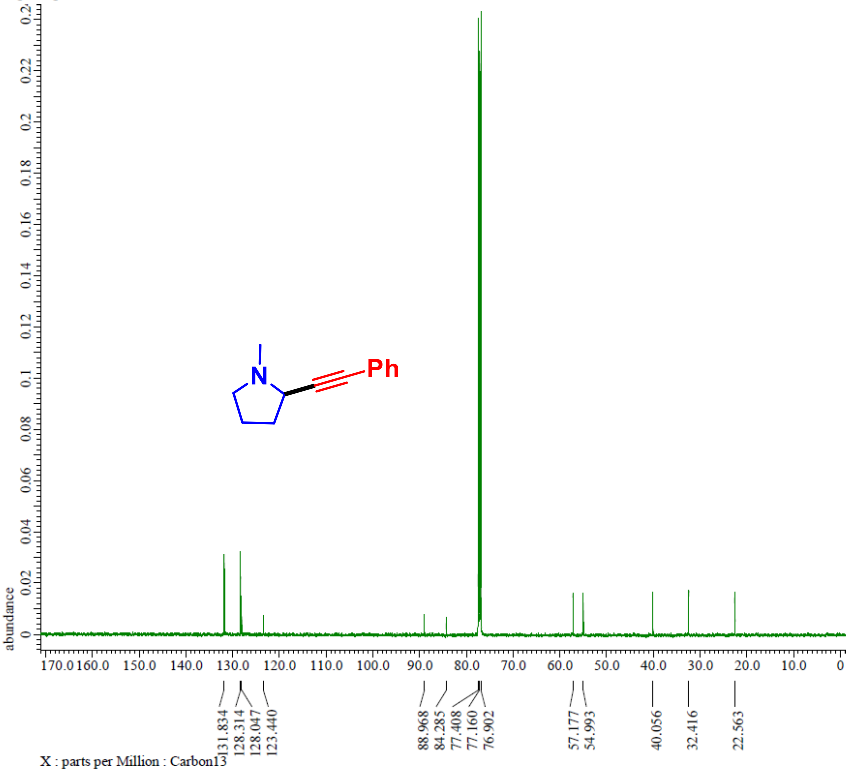

Supplementary Fig. 48. ^{1}H NMR and ^{13}C NMR of **3ba**, recorded at ~25 °C in $CDCl_3$ at 500 MHz and 126 MHz, respectively.

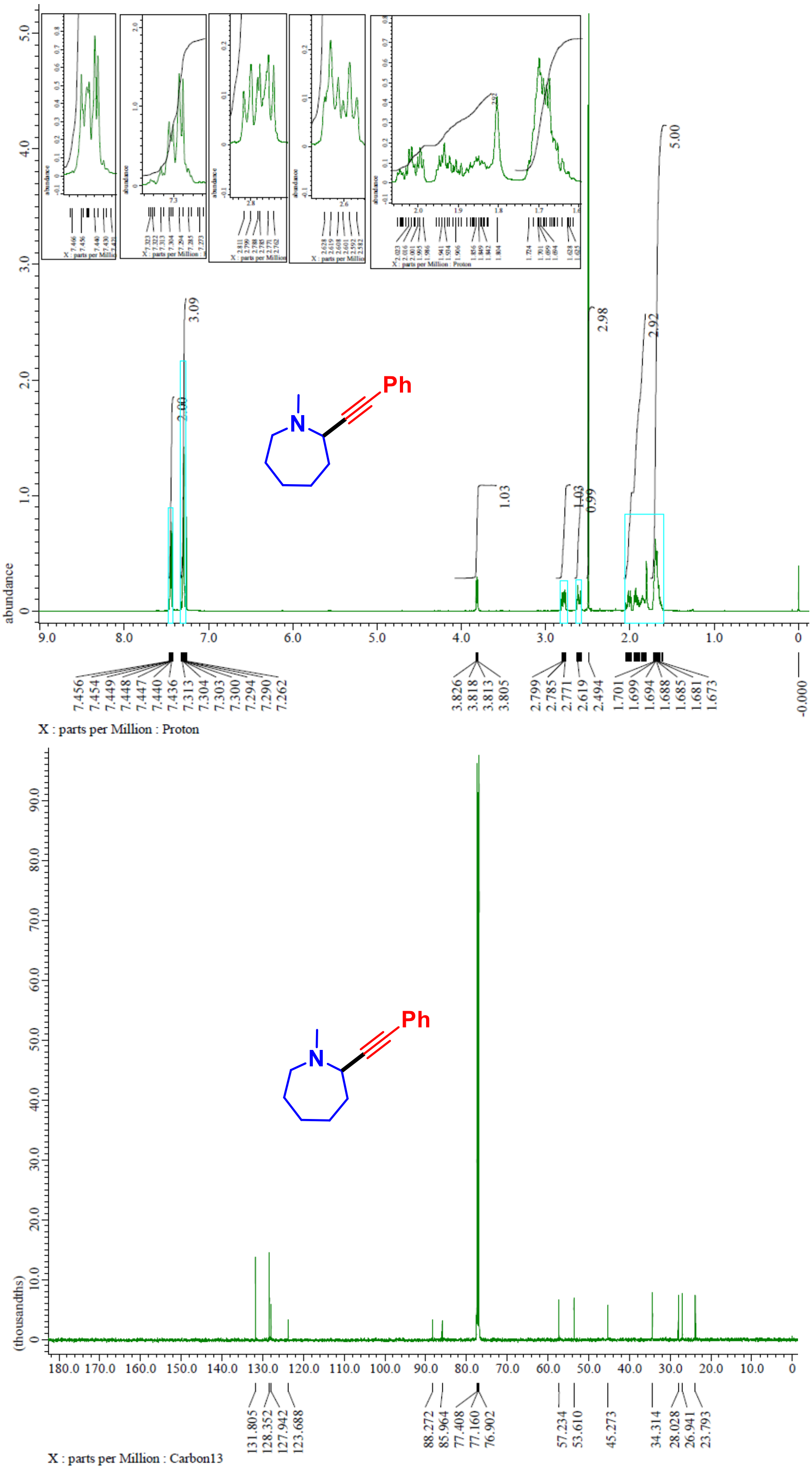

Supplementary Fig. 49. ^{1}H NMR and ^{13}C NMR of **3ca**, recorded at ~25 °C in $CDCl_3$ at 500 MHz and 126 MHz, respectively.

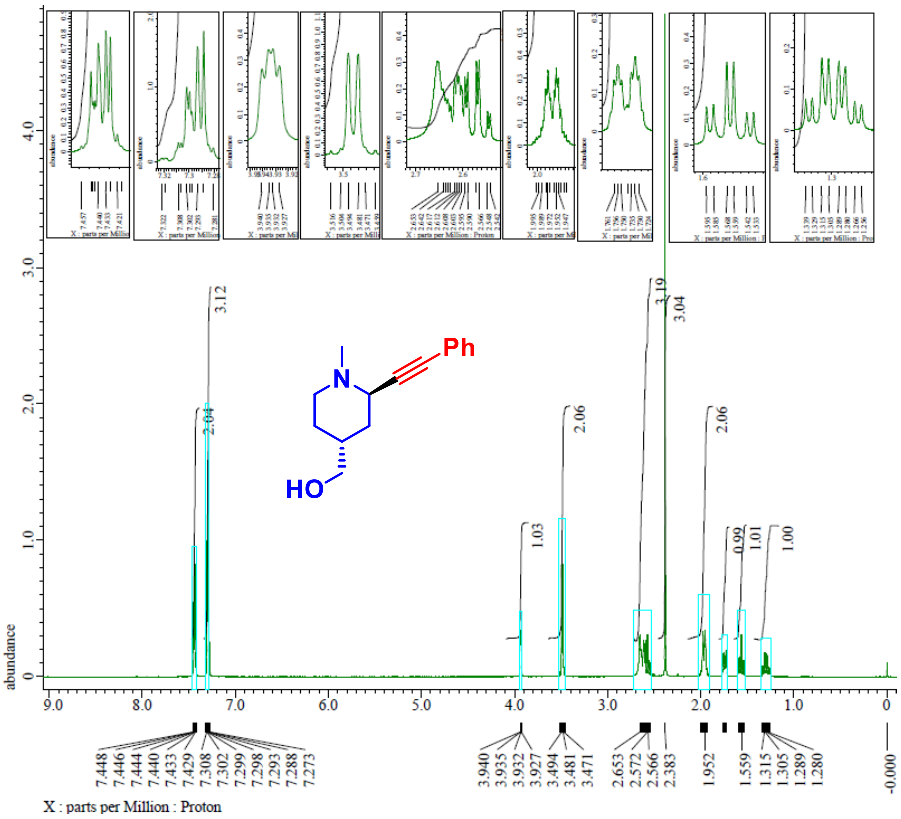

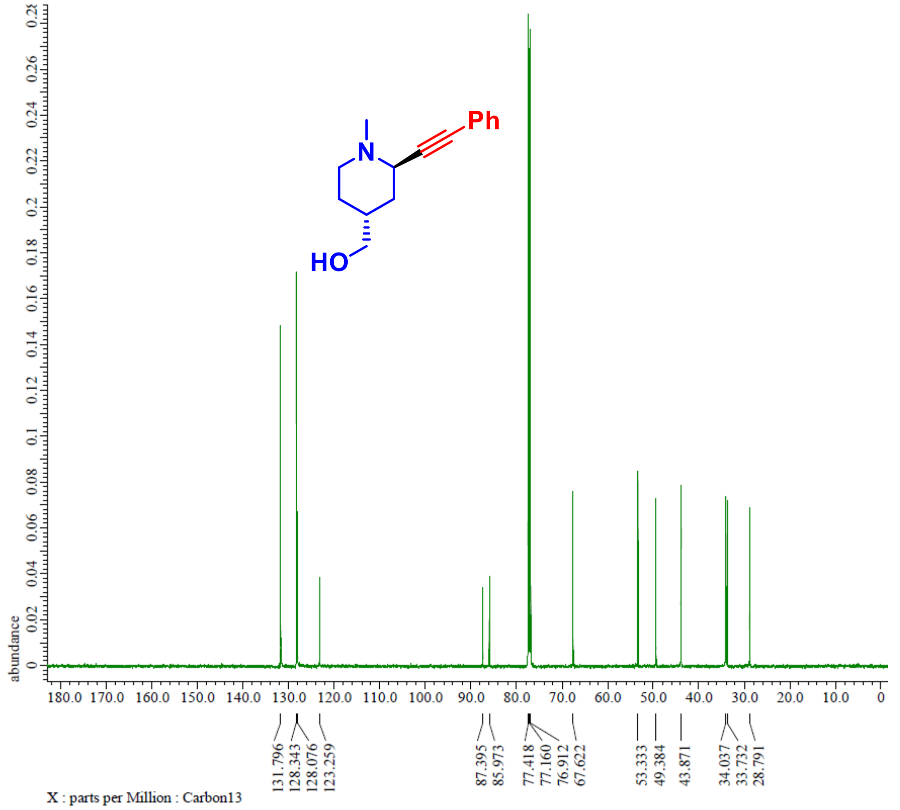

Supplementary Fig. 50. ^{1}H NMR and ^{13}C NMR of **3da**, recorded at ~25 °C in $CDCl_3$ at 500 MHz and 126 MHz, respectively.

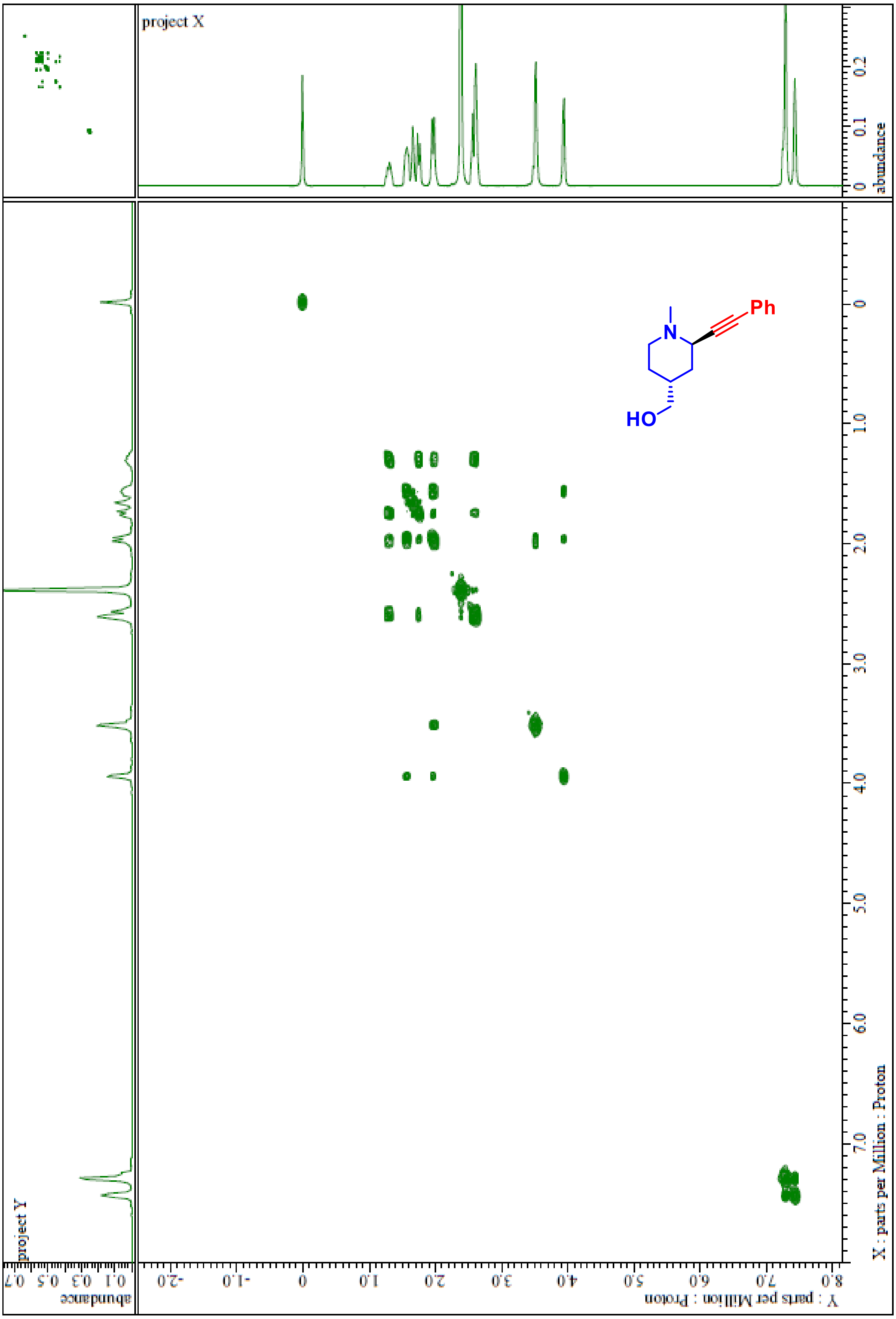

Supplementary Fig. 51. ^{1}H–^{1}H COSY of **3da**, recorded at ~25 °C in $CDCl_3$ at 500 MHz.

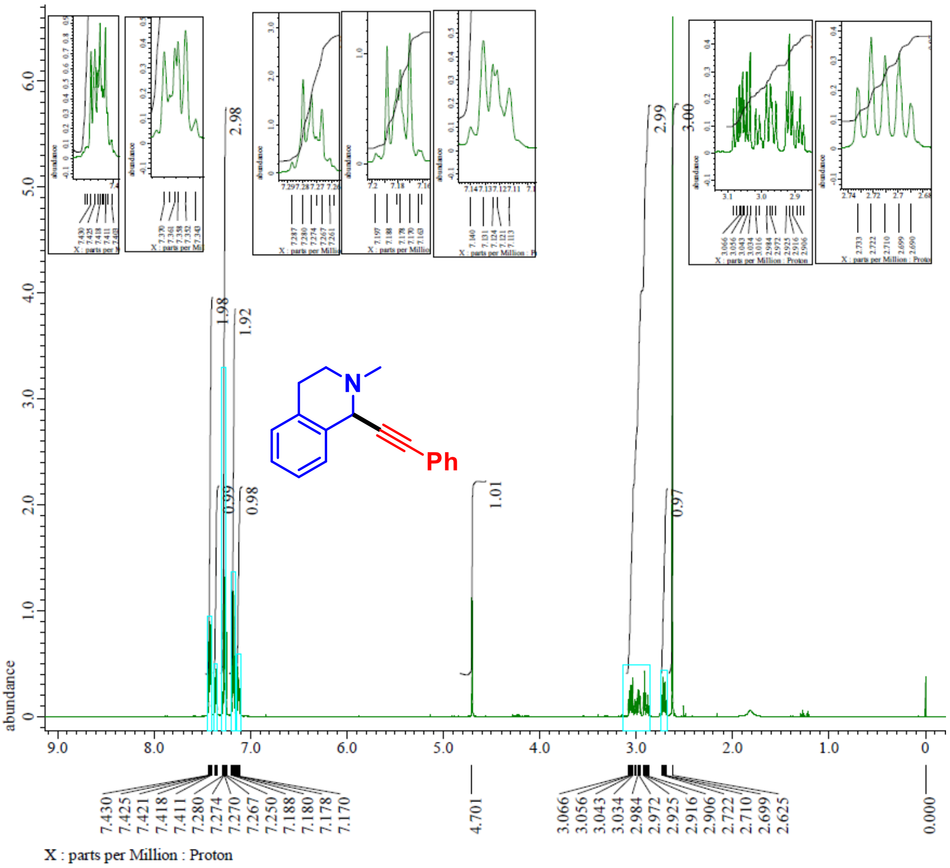

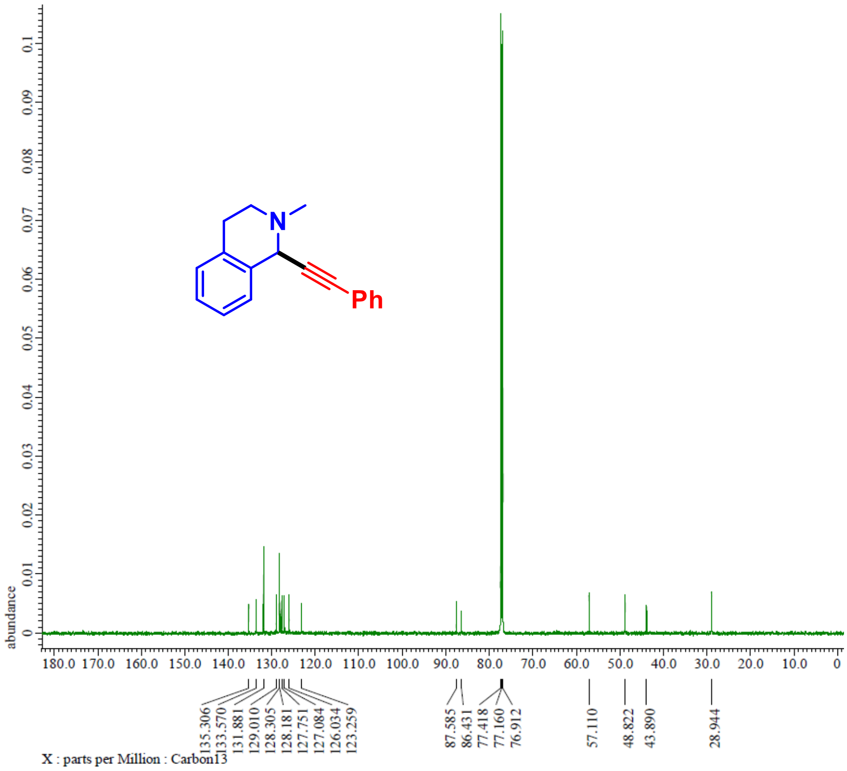

Supplementary Fig. 52. ^{1}H NMR and ^{13}C NMR of **3ea**, recorded at ~25 °C in $CDCl_3$ at 500 MHz and 126 MHz, respectively.

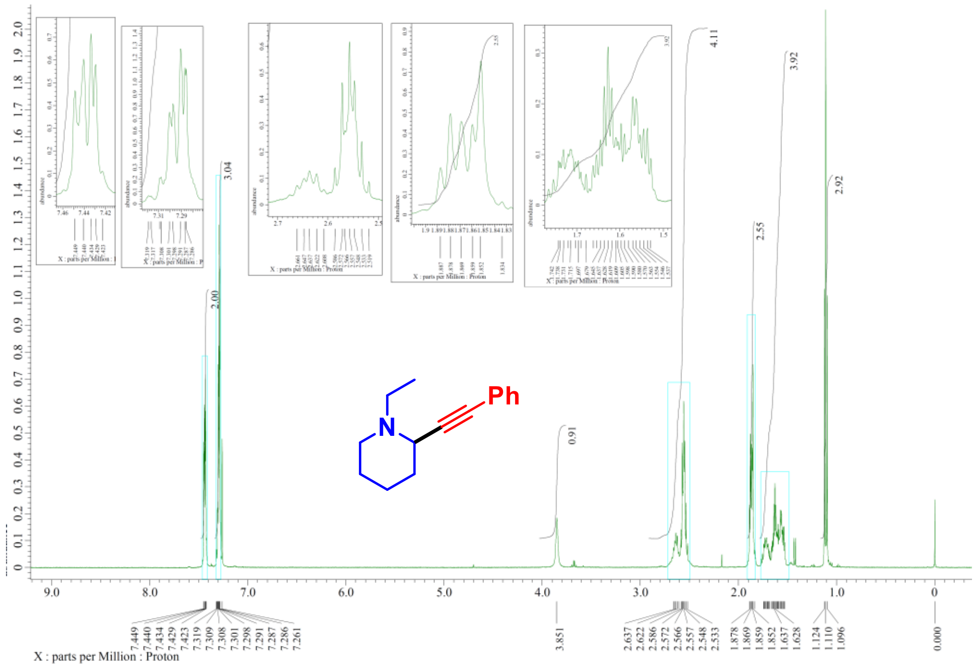

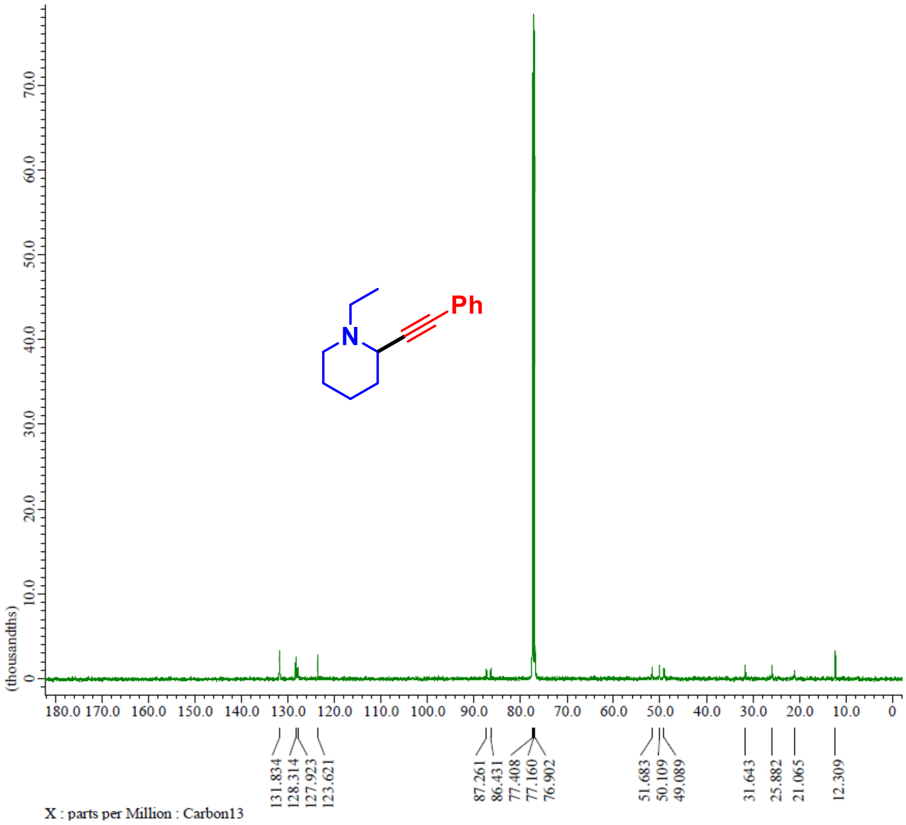

Supplementary Fig. 53. ¹H NMR and ¹³C NMR of **3fa**, recorded at ~25 °C in $CDCl_3$ at 500 MHz and 126 MHz, respectively.

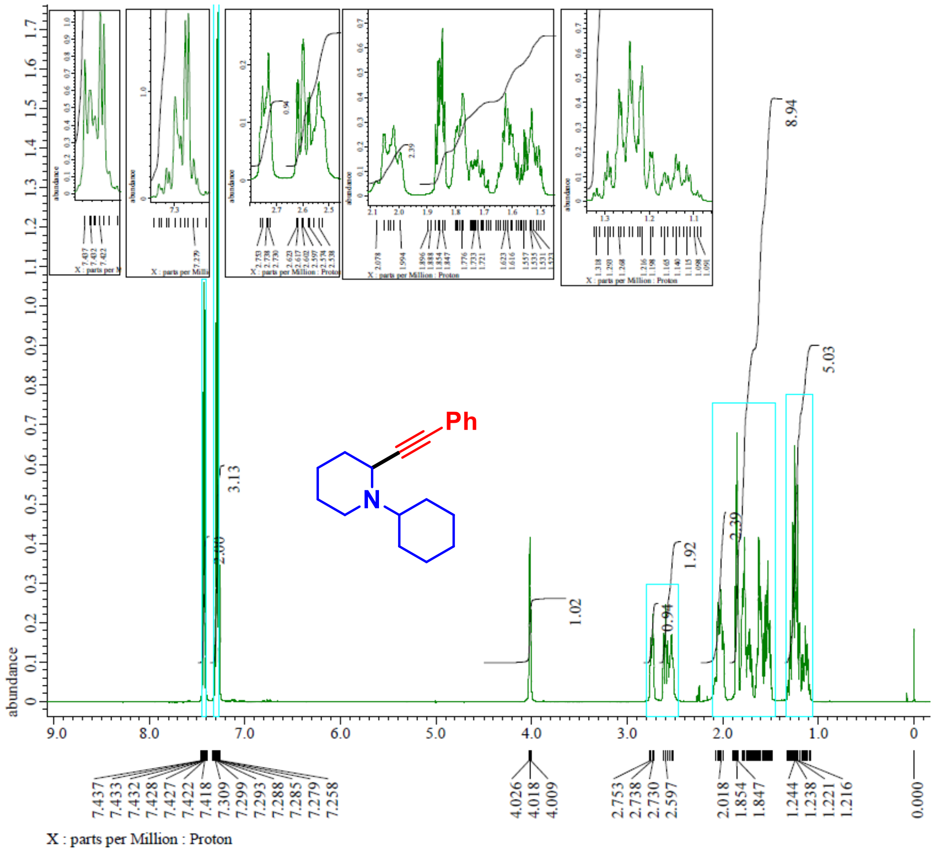

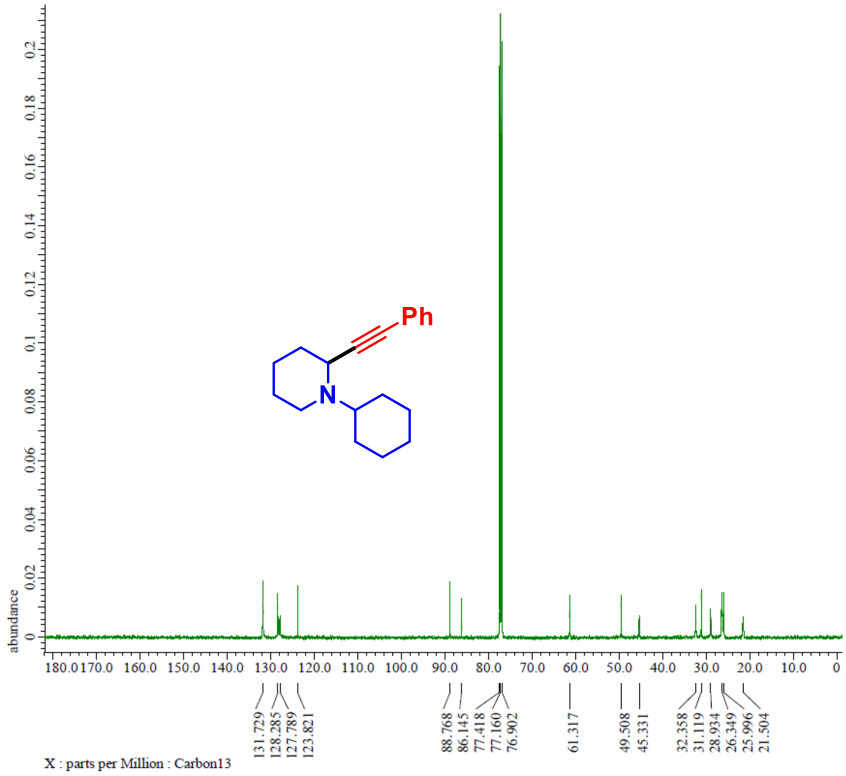

Supplementary Fig. 54. ^{1}H NMR and ^{13}C NMR of **3ga**, recorded at ~25 °C in $CDCl_3$ at 500 MHz and 126 MHz, respectively.

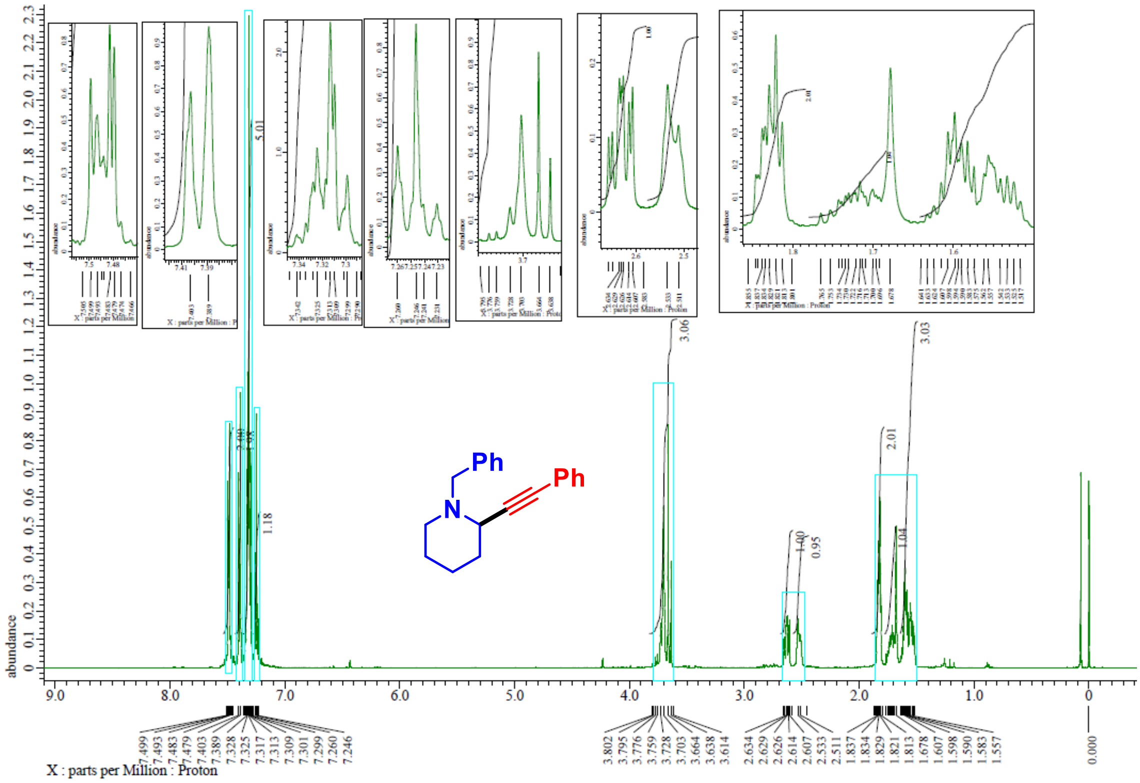

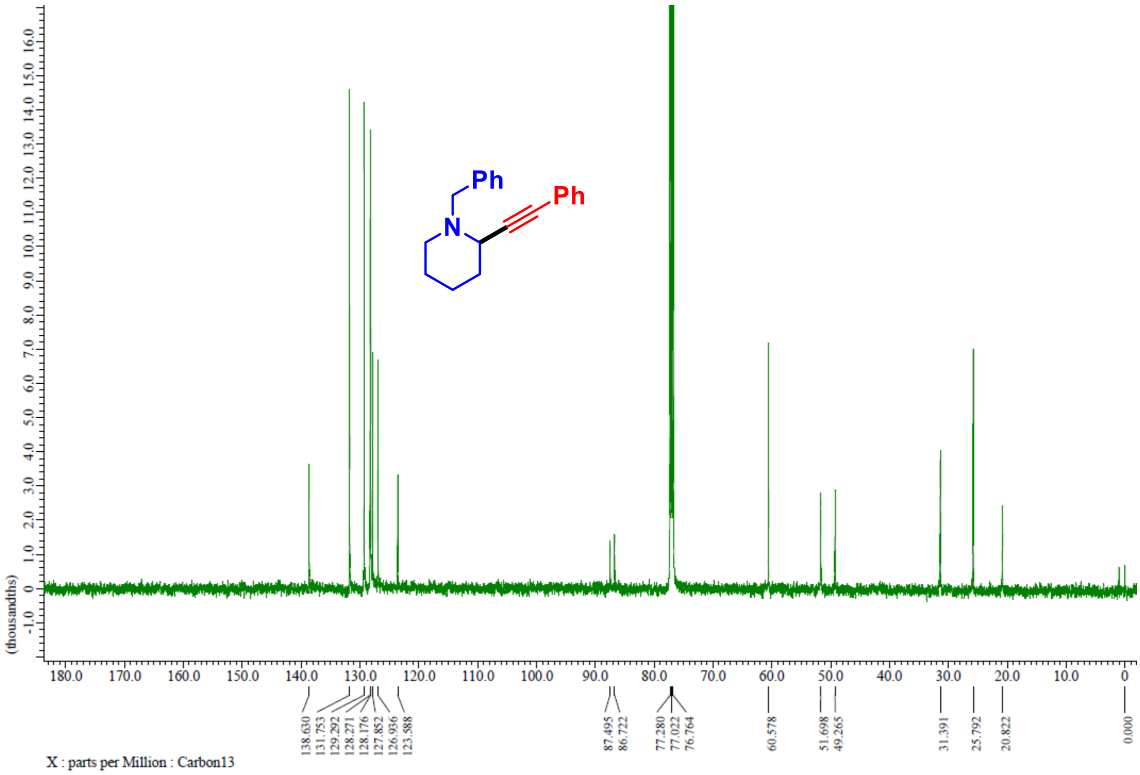

Supplementary Fig. 55. ^{1}H NMR and ^{13}C NMR of **3ha**, recorded at ~25 °C in $CDCl_3$ at 500 MHz and 126 MHz, respectively.

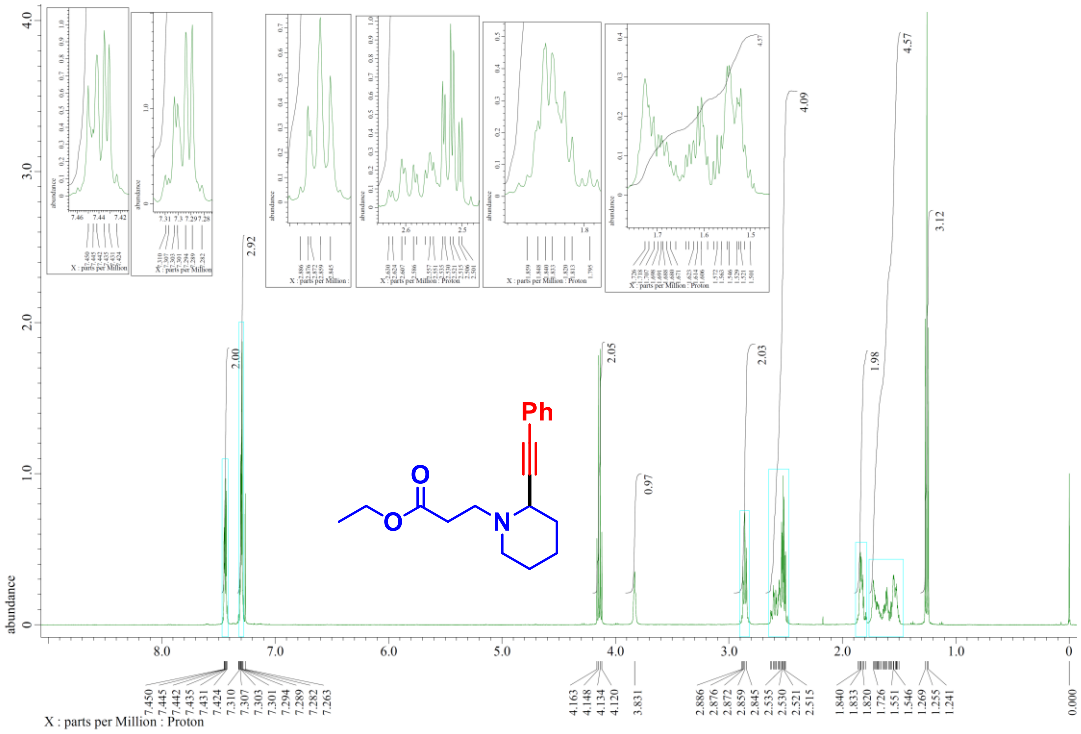

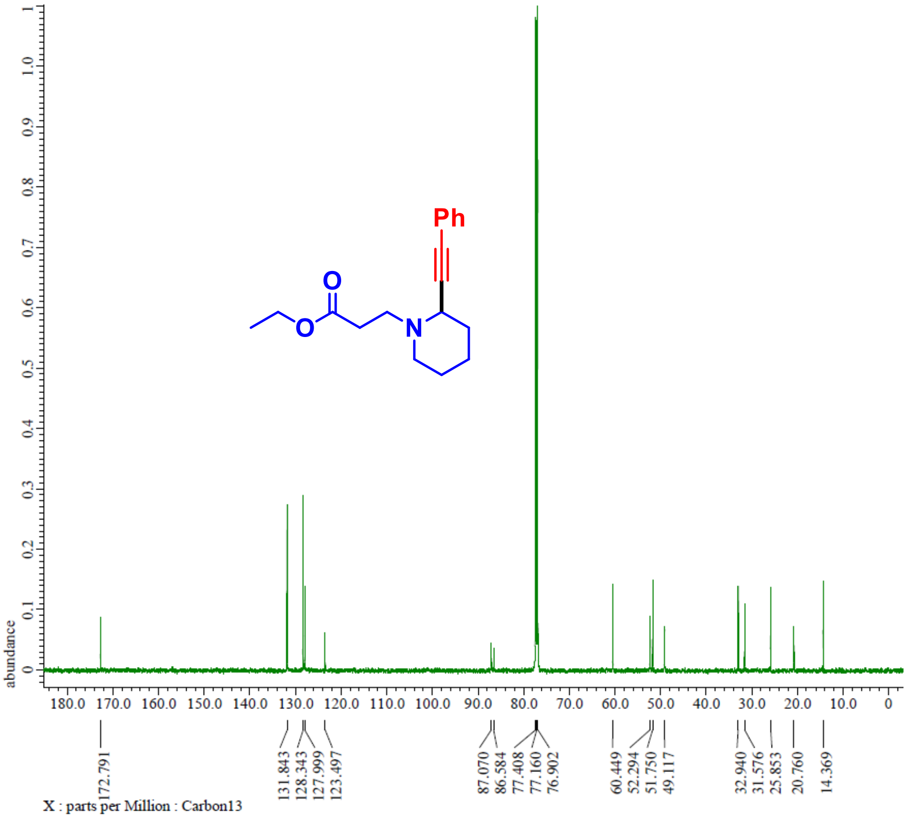

Supplementary Fig. 56. ^{1}H NMR and ^{13}C NMR of **3ia**, recorded at ~25 °C in $CDCl_3$ at 500 MHz and 126 MHz, respectively.

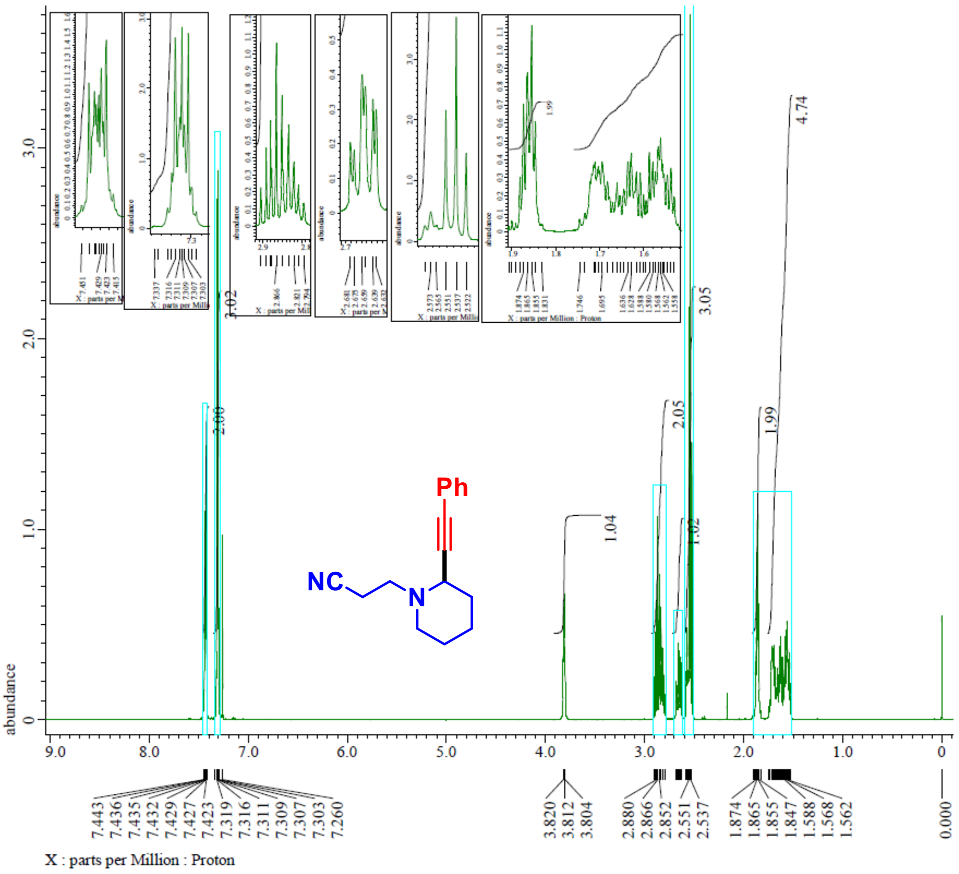

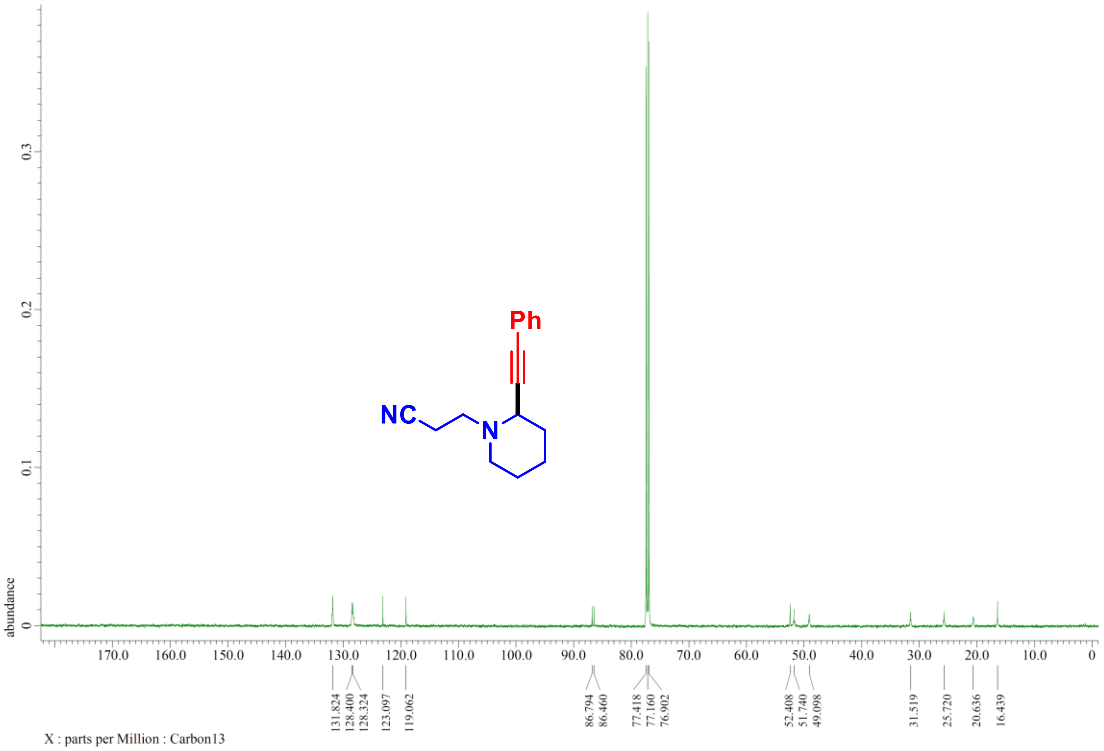

Supplementary Fig. 57. ^{1}H NMR and ^{13}C NMR of **3ja**, recorded at ~25 °C in $CDCl_3$ at 500 MHz and 126 MHz, respectively.

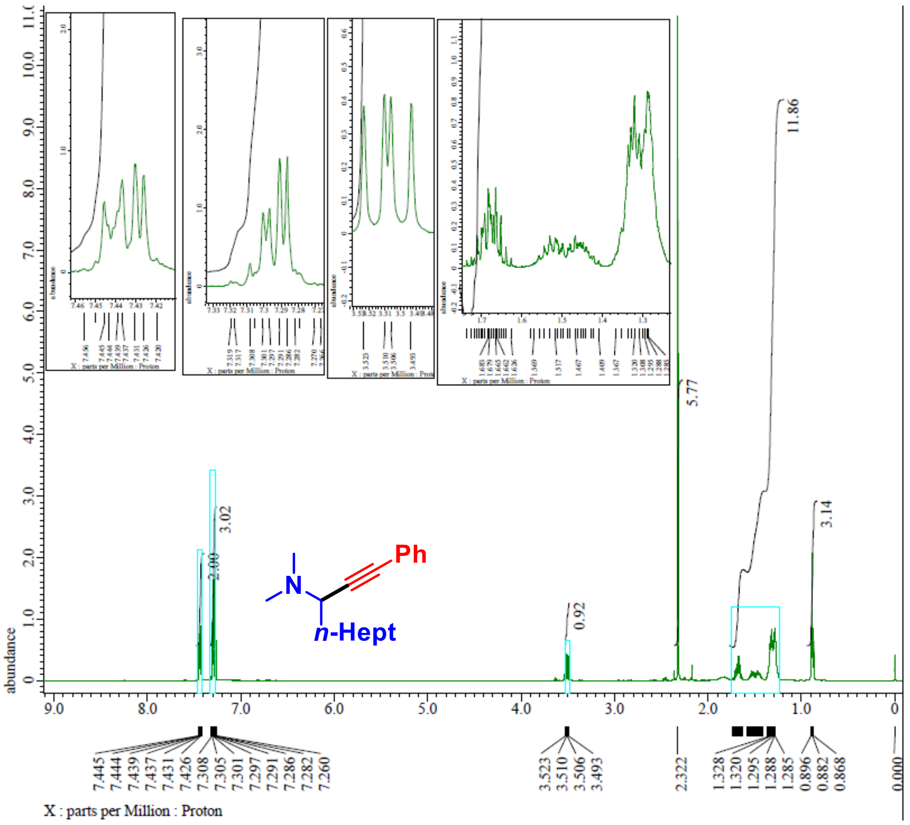

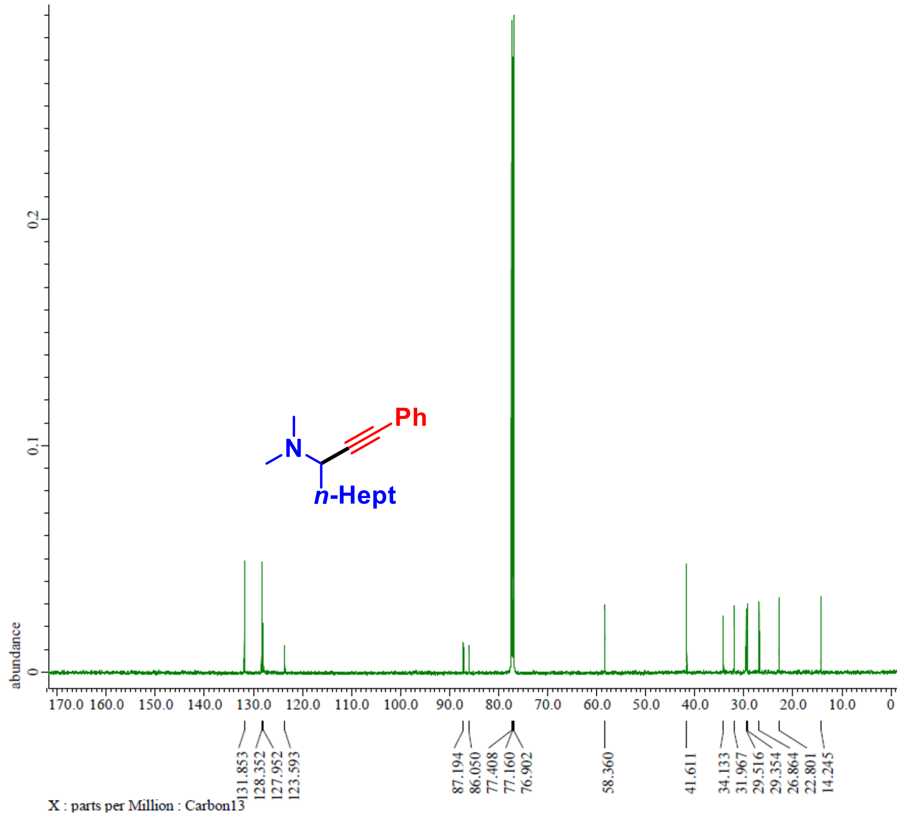

Supplementary Fig. 58. ^{1}H NMR and ^{13}C NMR of **3ka**, recorded at ~25 °C in $CDCl_3$ at 500 MHz and 126 MHz, respectively.

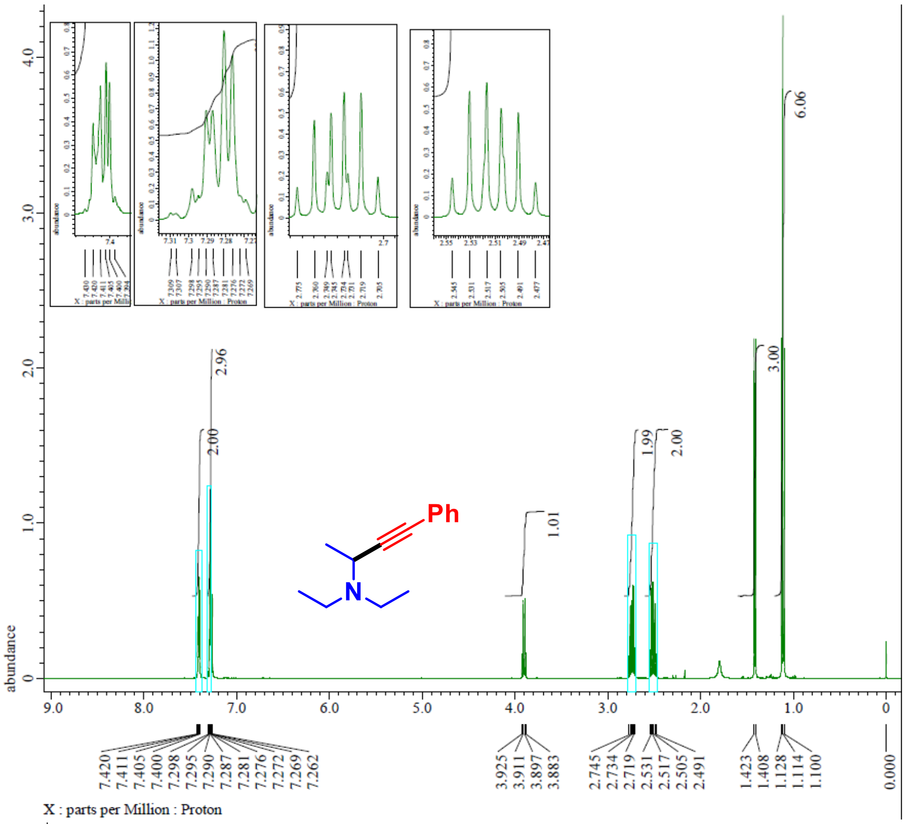

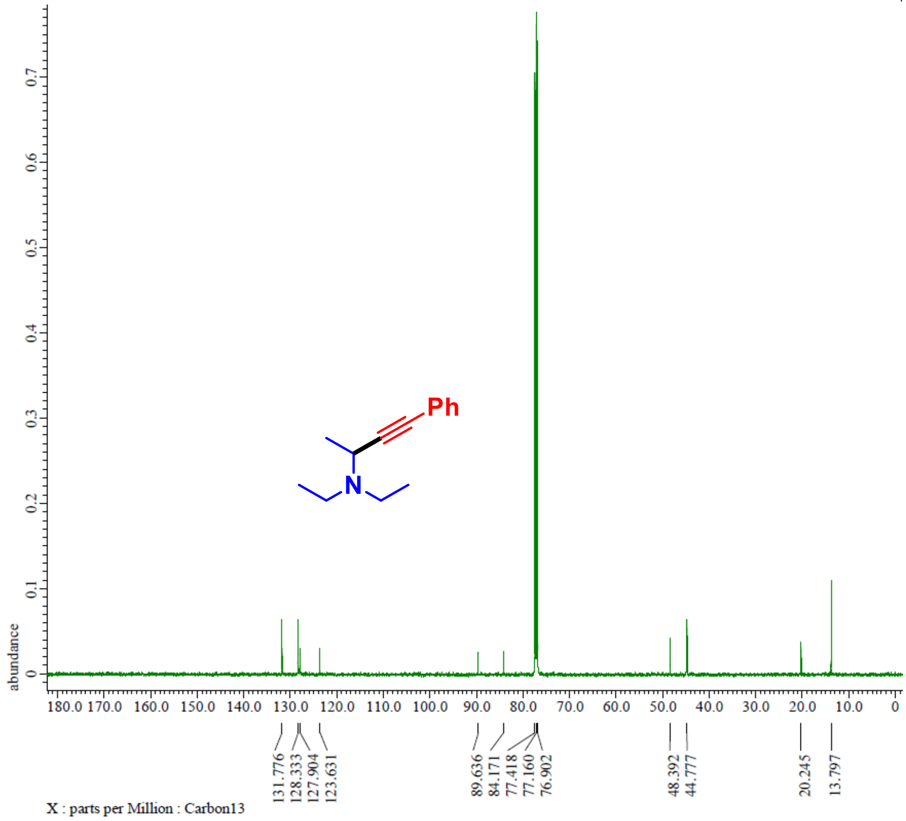

Supplementary Fig. 59. ^{1}H NMR and ^{13}C NMR of **3la**, recorded at ~25 °C in $CDCl_3$ at 500 MHz and 126 MHz, respectively.

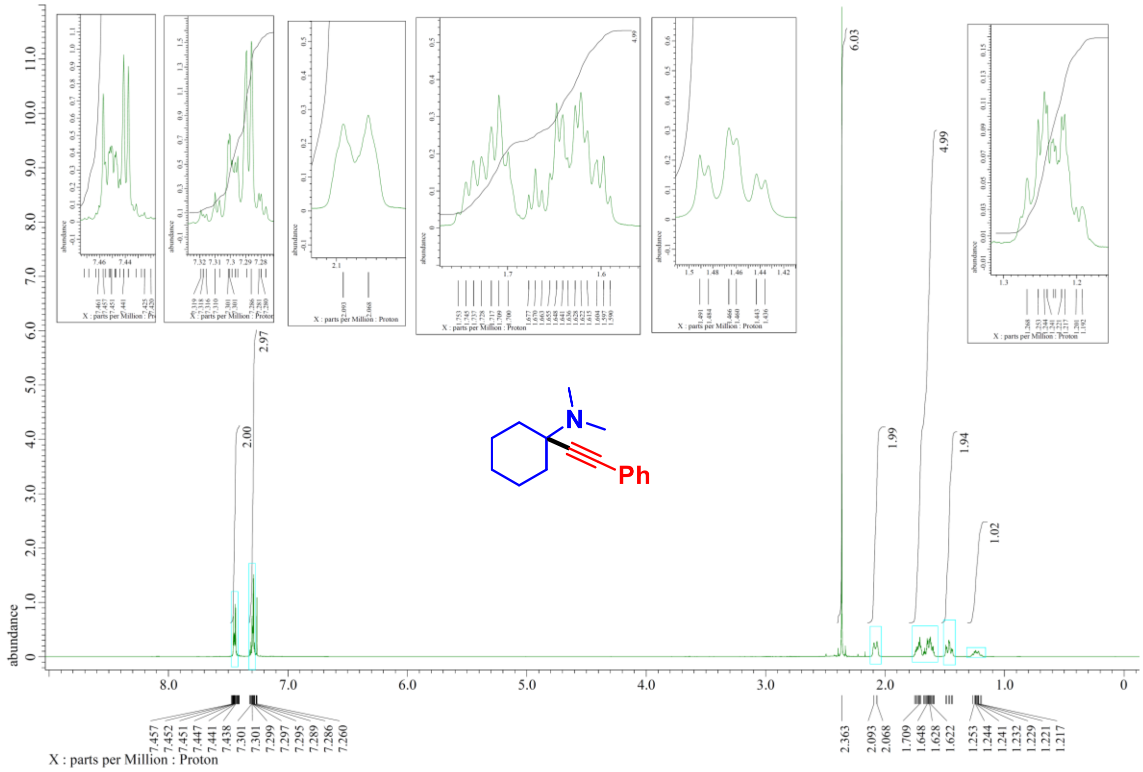

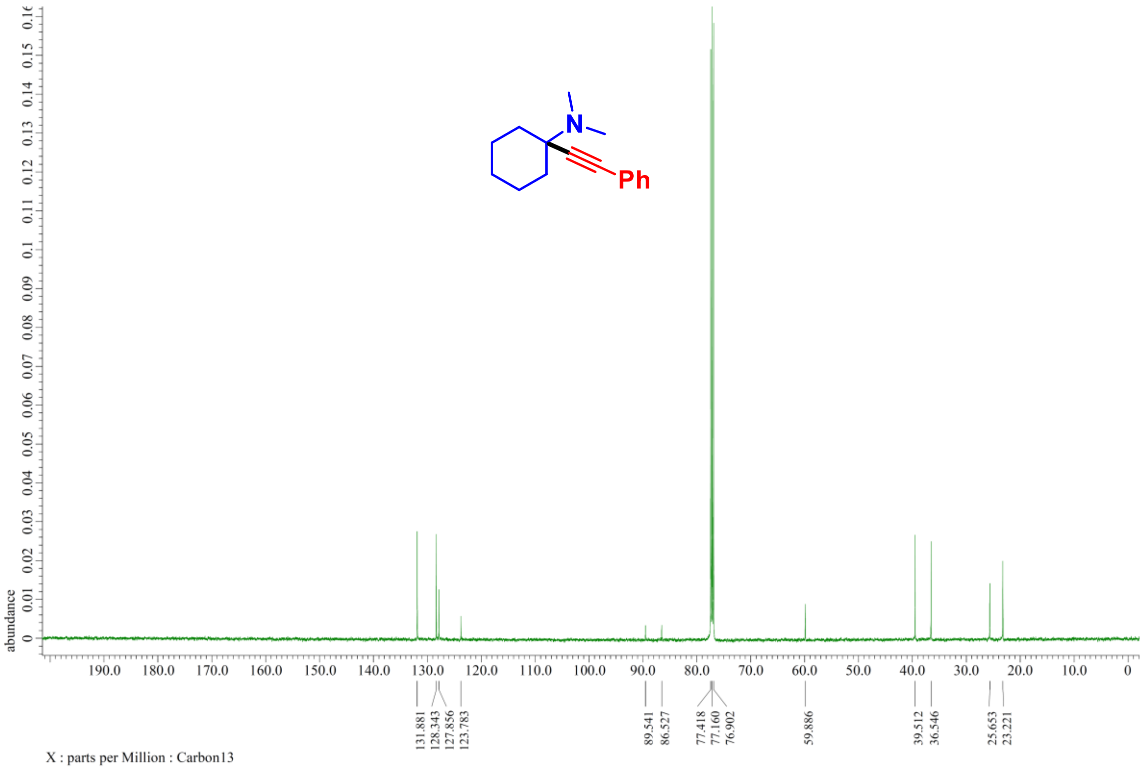

Supplementary Fig. 60. ^{1}H NMR and ^{13}C NMR of **3ma**, recorded at ~25 °C in $CDCl_3$ at 500 MHz and 126 MHz, respectively.

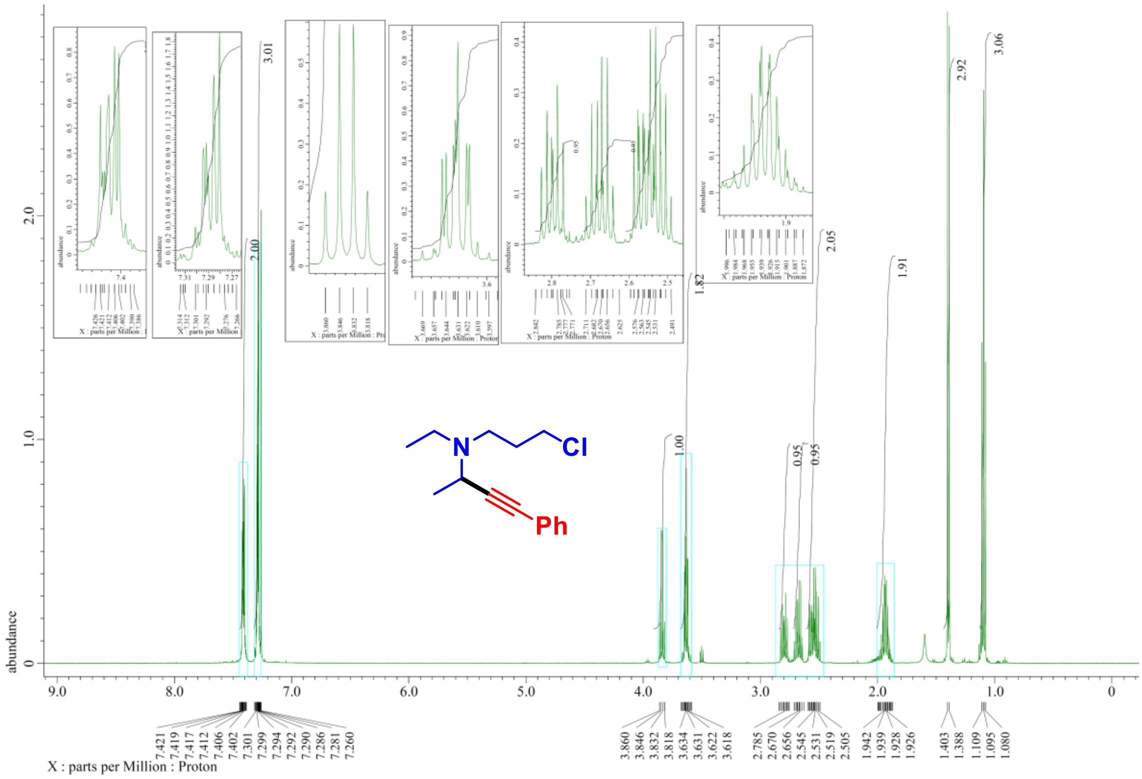

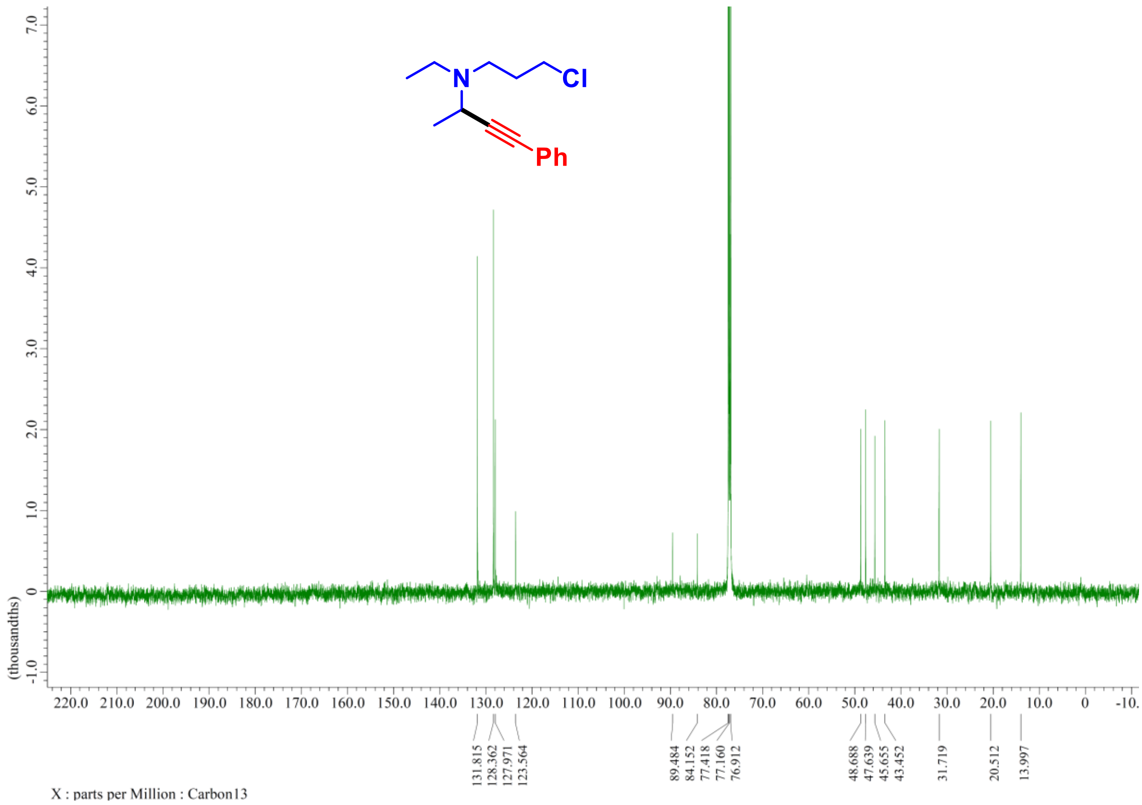

Supplementary Fig. 61. ^{1}H NMR and ^{13}C NMR of **3na**, recorded at ~25 °C in $CDCl_3$ at 500 MHz and 126 MHz, respectively.

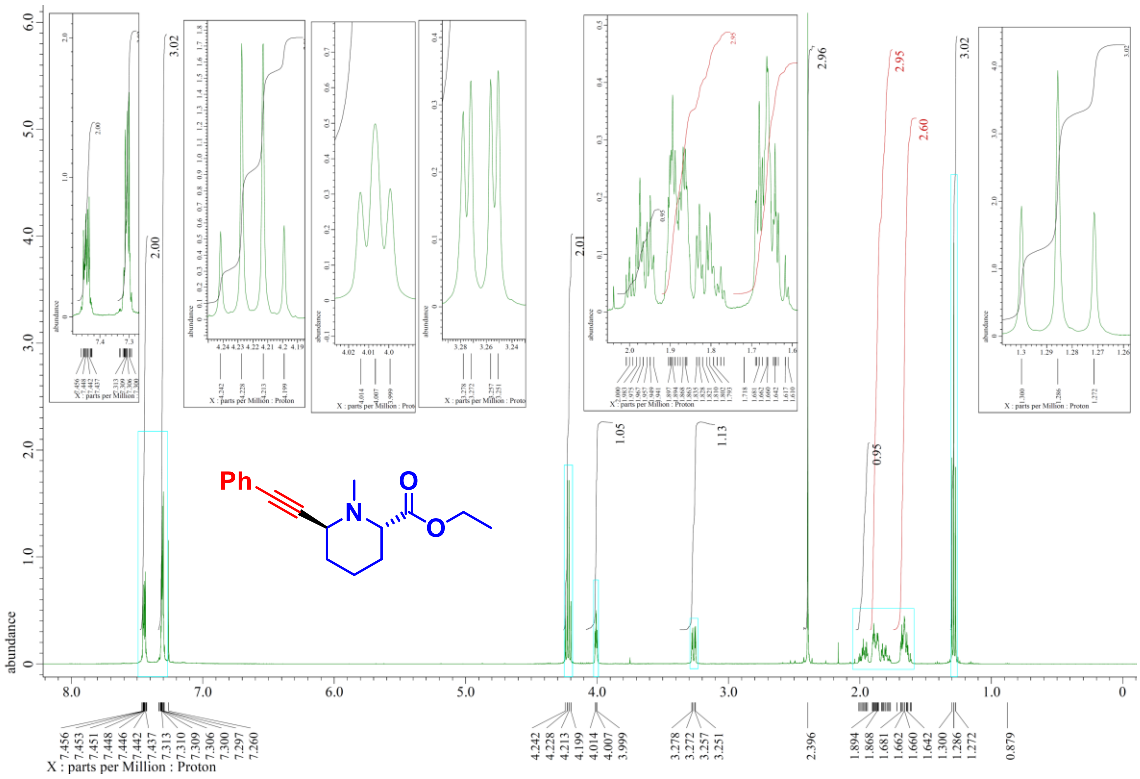

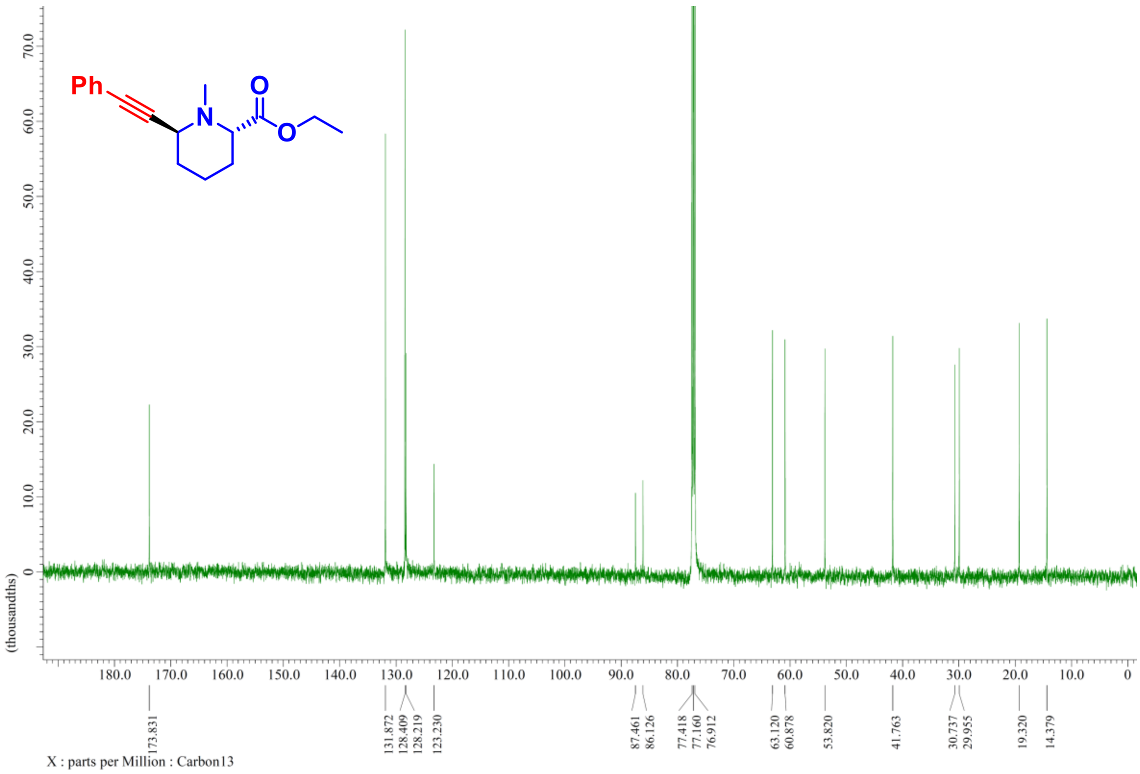

Supplementary Fig. 62. ^{1}H NMR and ^{13}C NMR of **3oa** (*trans*), recorded at ~25 °C in $CDCl_3$ at 500 MHz and 126 MHz, respectively.

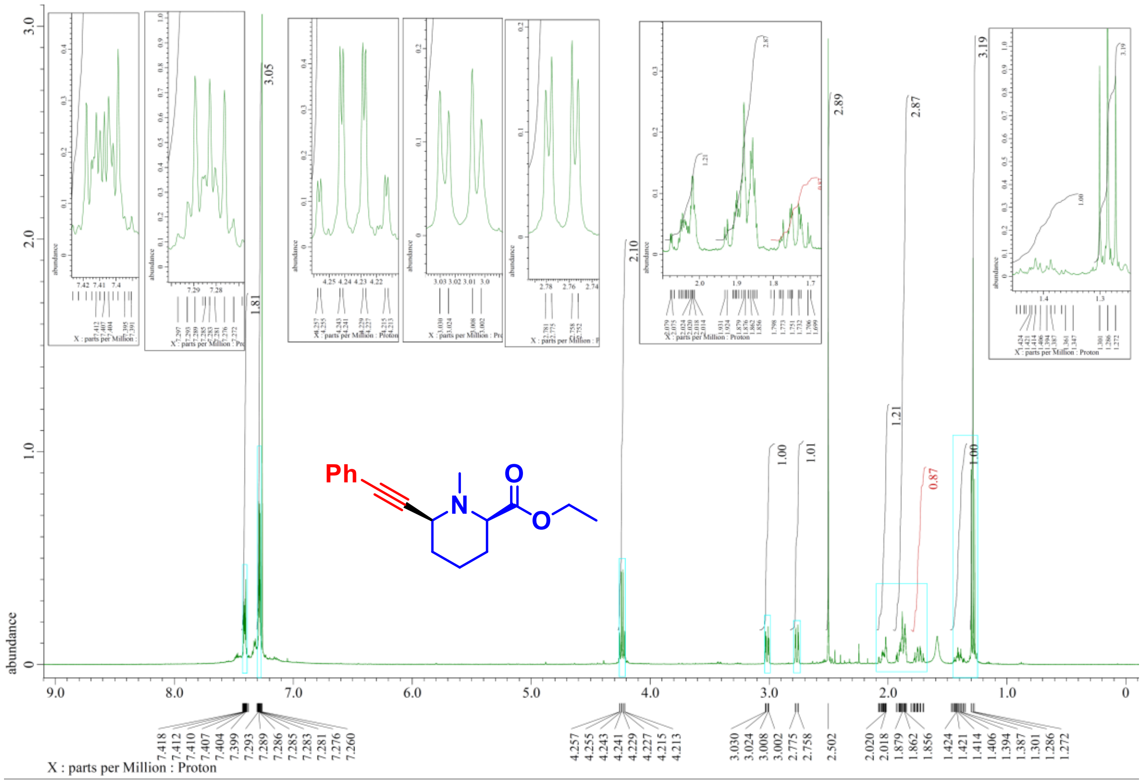

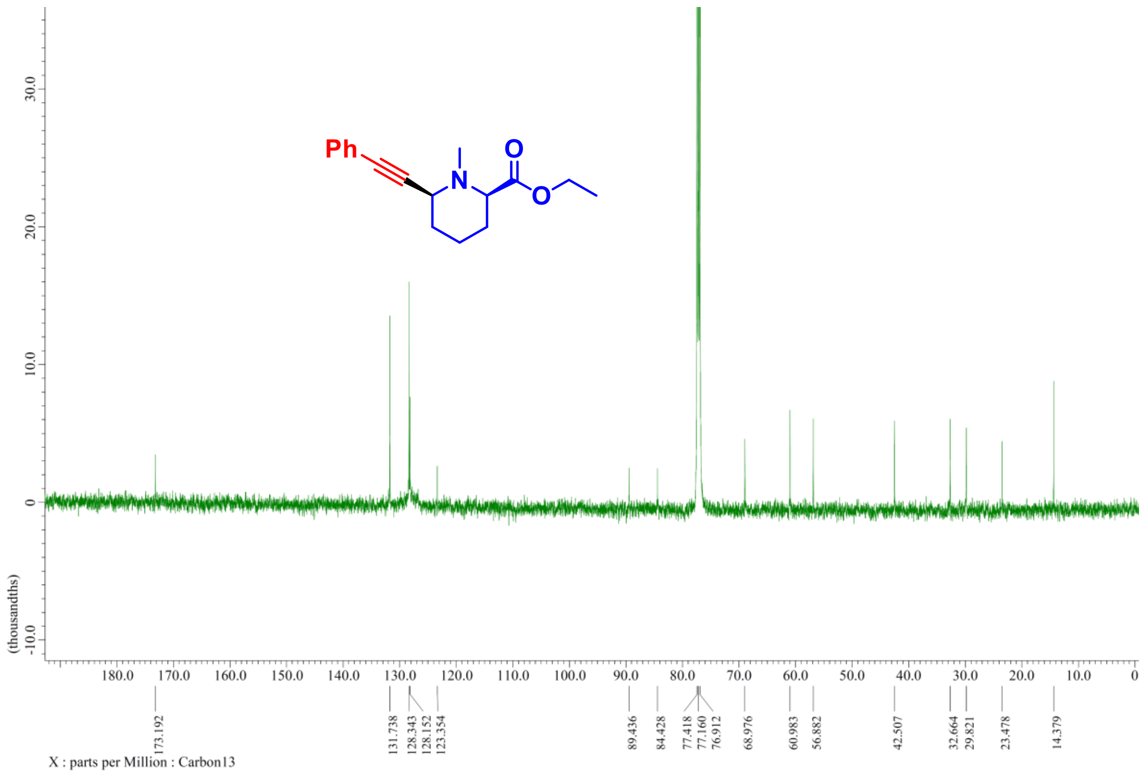

Supplementary Fig. 63. ^{1}H NMR and ^{13}C NMR of **3oa** (*cis*), recorded at ~25 °C in $CDCl_3$ at 500 MHz and 126 MHz, respectively.

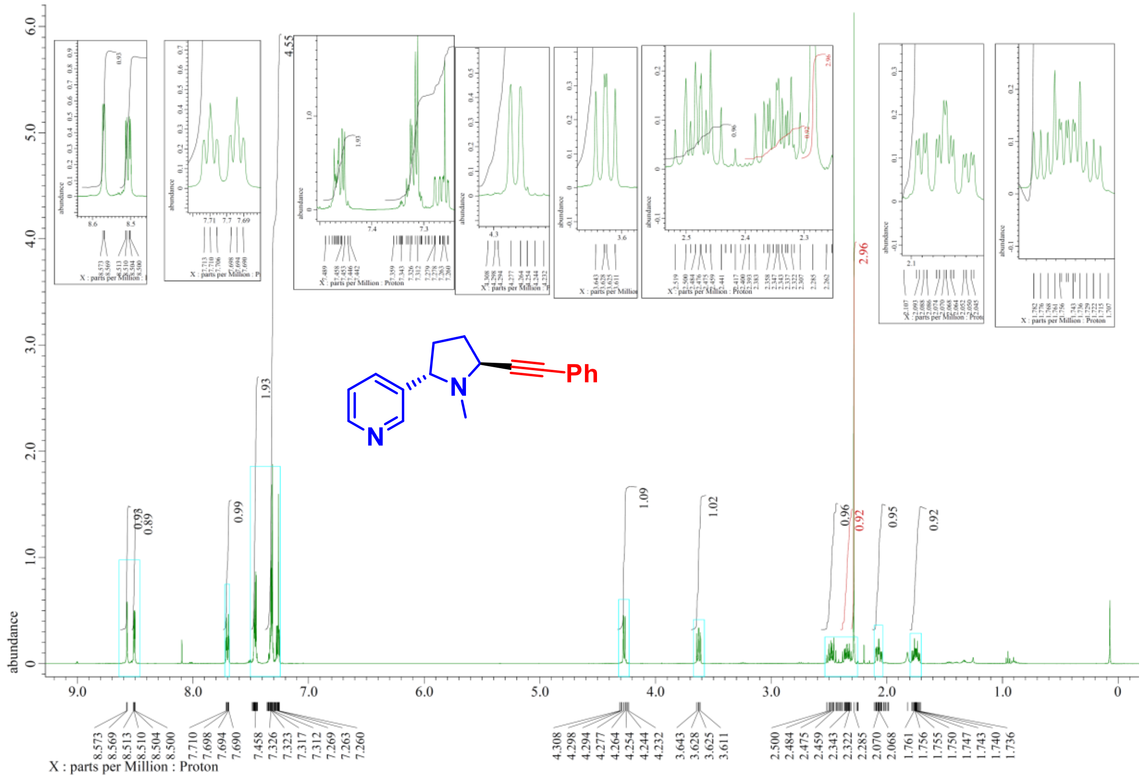

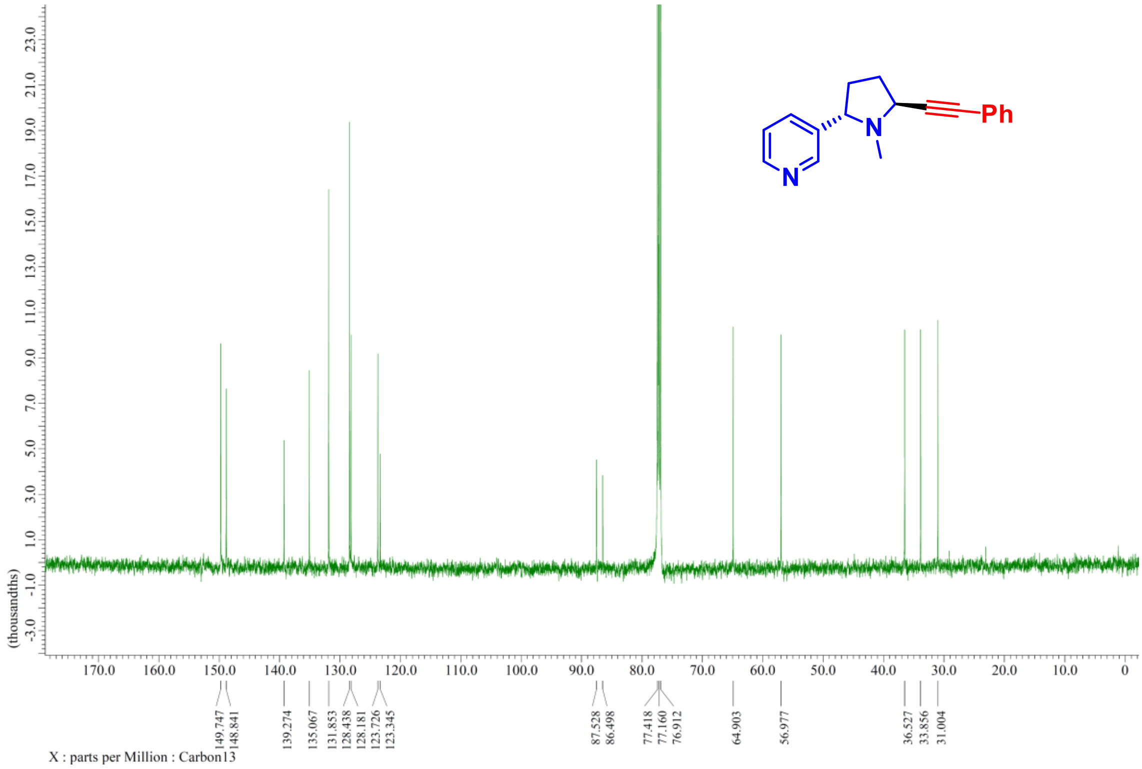

Supplementary Fig. 64. ^{1}H NMR and ^{13}C NMR of **3pa** (*trans*), recorded at ~25 °C in $CDCl_3$ at 500 MHz and 126 MHz, respectively. Eluent: hexane/EtOAc = 4/6 (1st), hexane/EtOAc = 3/7 (2nd).

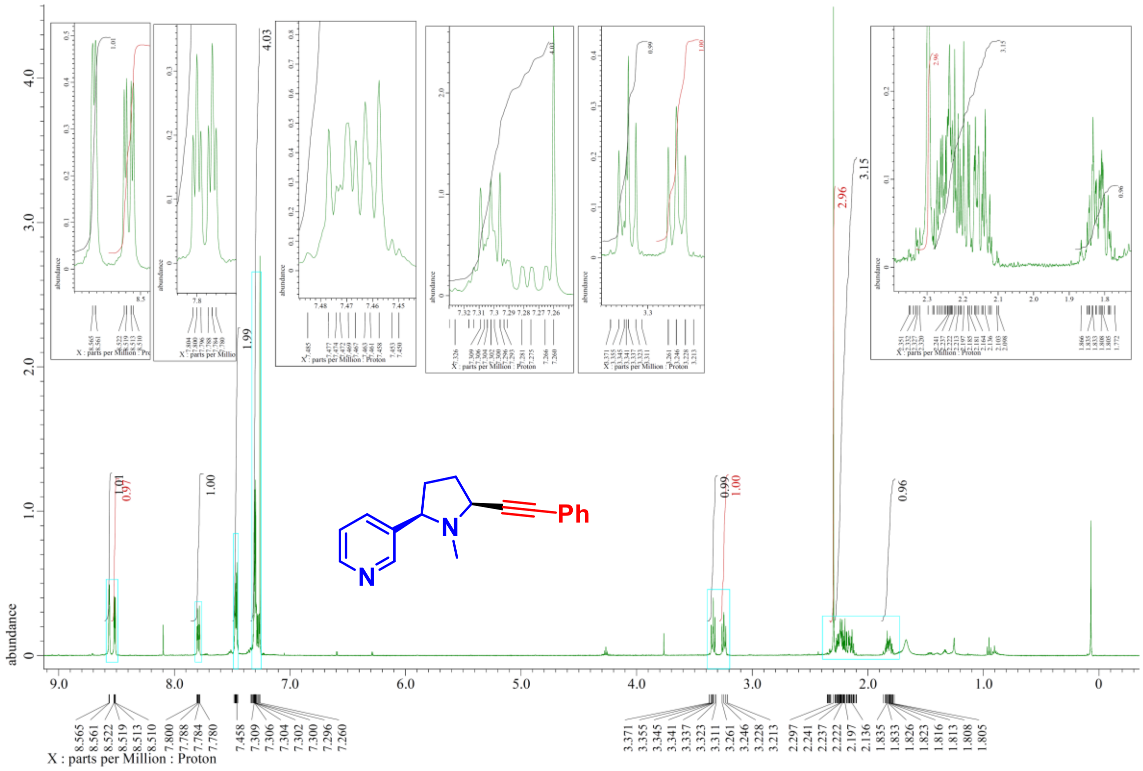

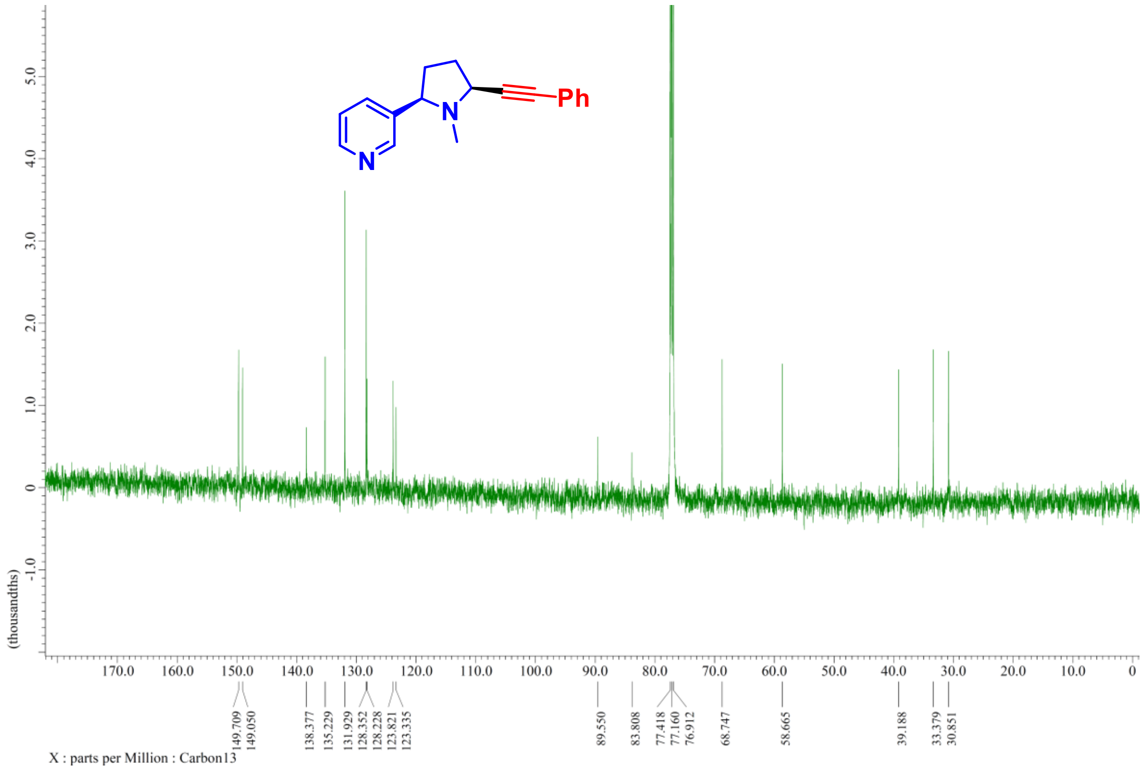

Supplementary Fig. 65. ^{1}H NMR and ^{13}C NMR of **3pa** (*cis*), recorded at ~25 °C in $CDCl_3$ at 500 MHz and 126 MHz, respectively. Eluent: hexane/EtOAc = 4/6 (1st), hexane/EtOAc = 3/7 (2nd).

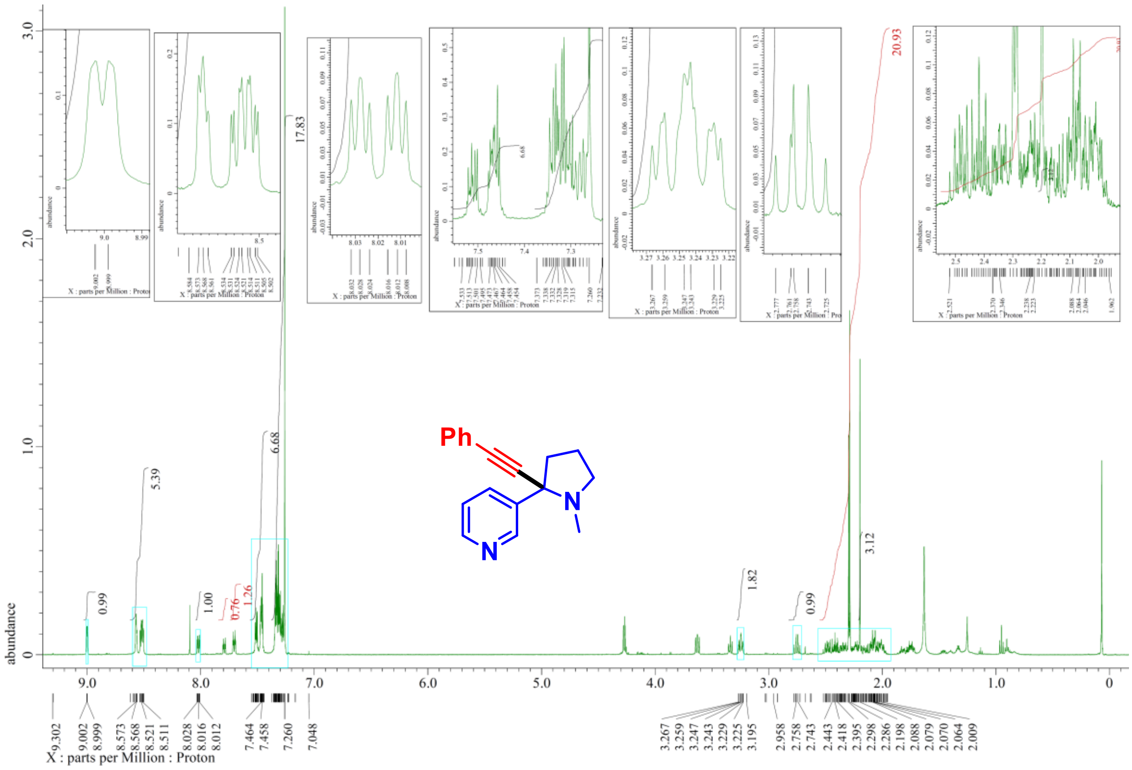

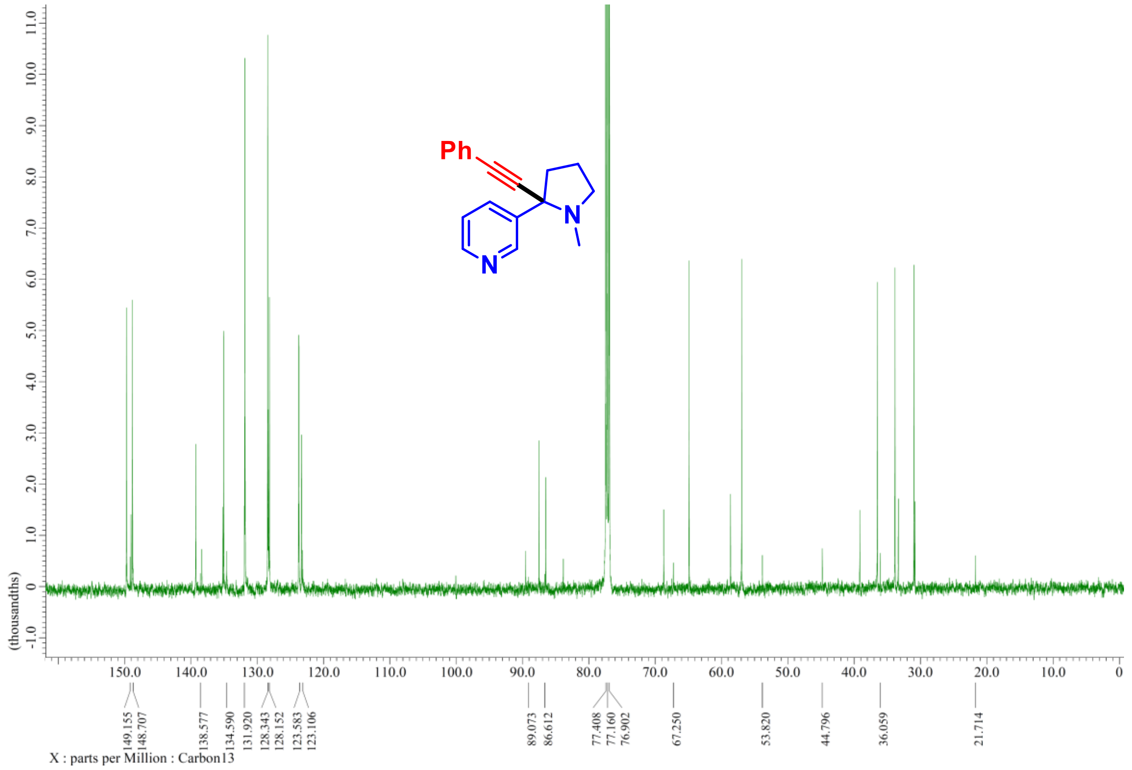

Supplementary Fig. 66. ^{1}H NMR and ^{13}C NMR of **4pa**, recorded at ~25 °C in $CDCl_3$ at 500 MHz and 126 MHz, respectively. Eluent: hexane/EtOAc = 4/6 (1st), hexane/EtOAc = 3/7 (2nd). Peaks of the *regio*-isomer were picked up from the mixture of *trans*/*cis*/*regio*-isomers.

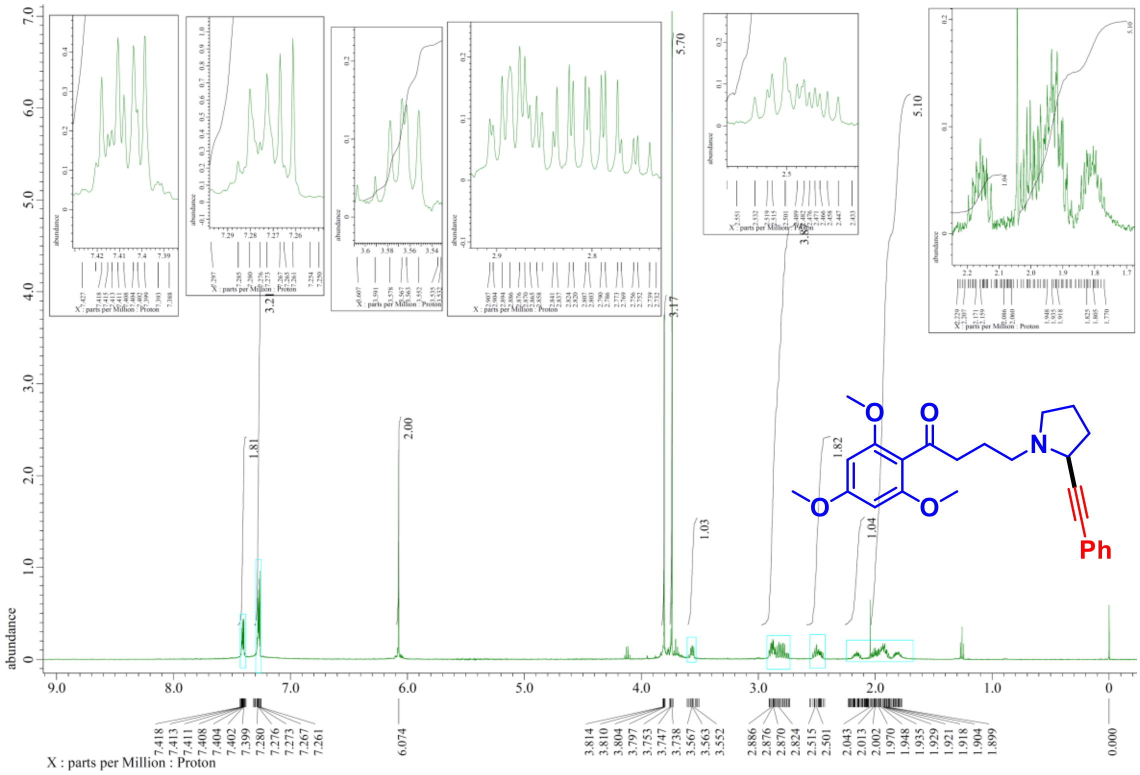

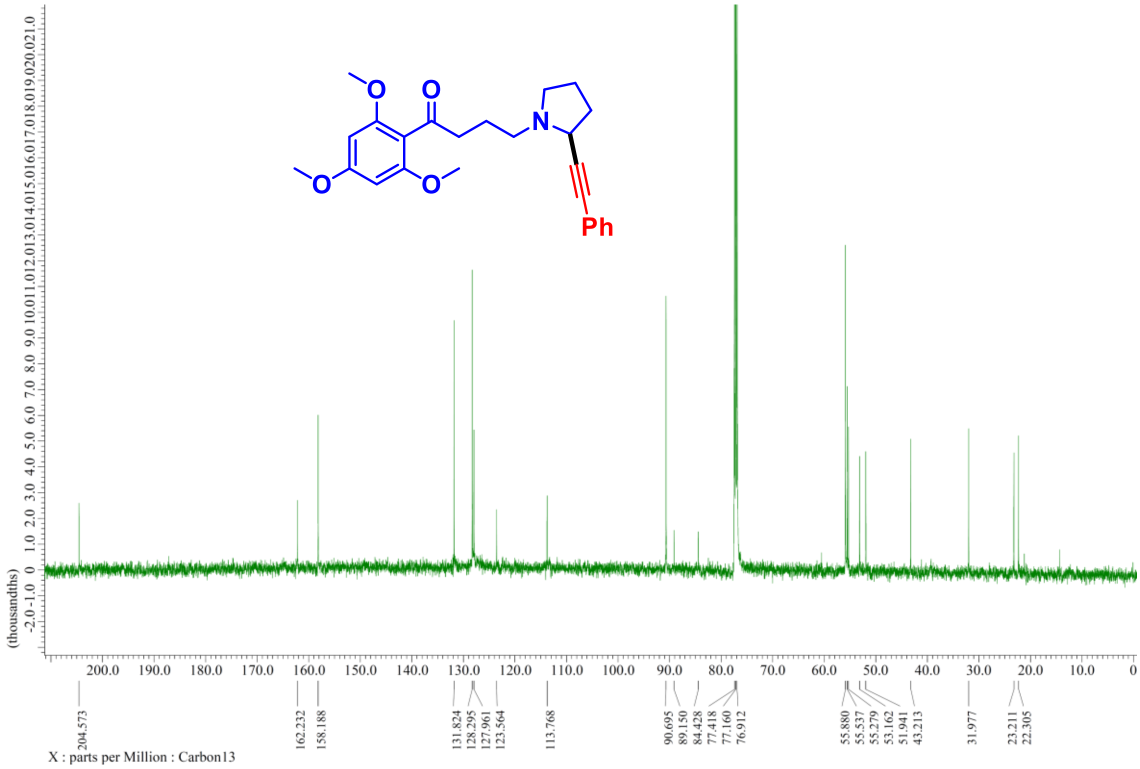

Supplementary Fig. 67. ^{1}H NMR and ^{13}C NMR of **3qa**, recorded at ~25 °C in $CDCl_3$ at 500 MHz and 126 MHz, respectively.

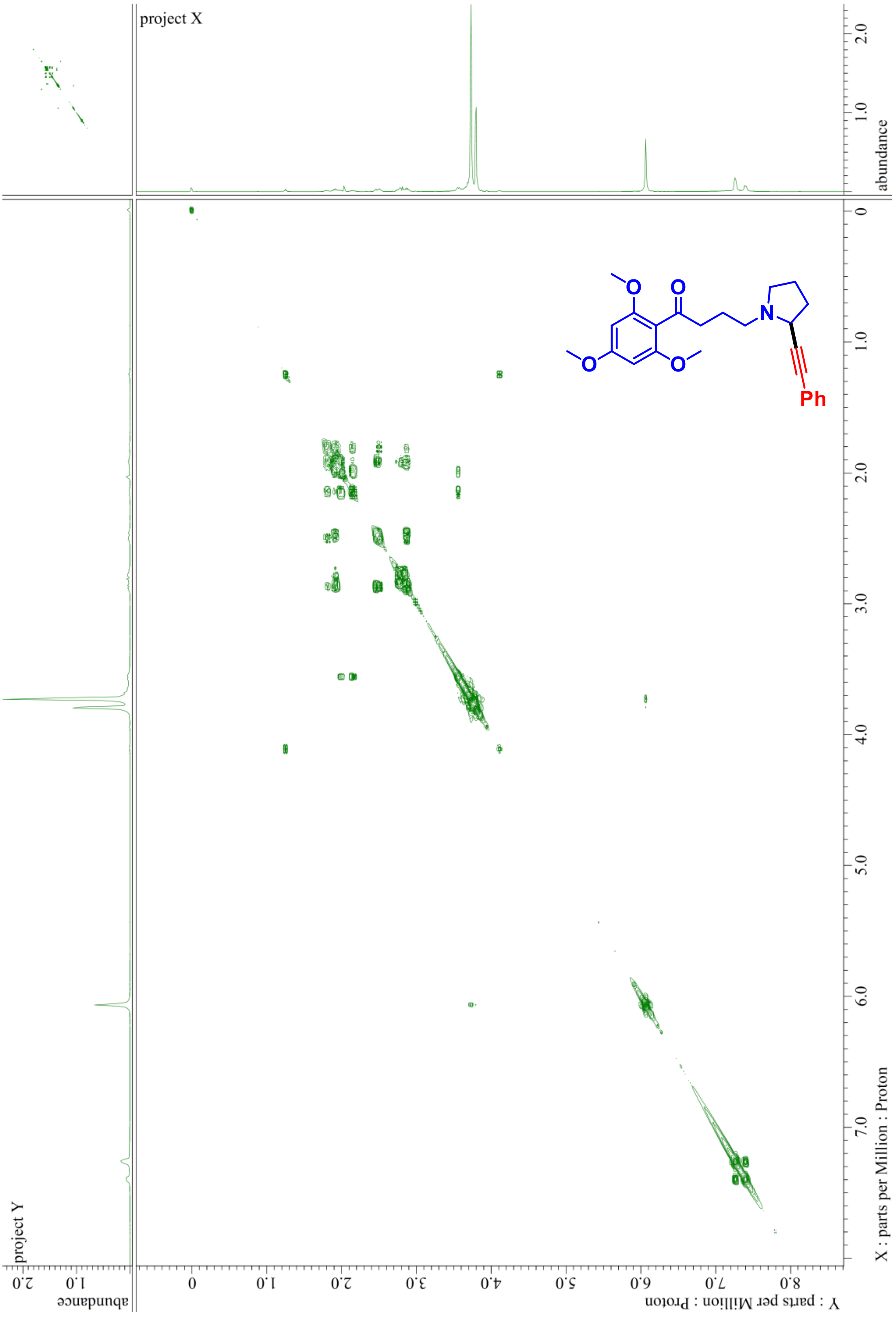

Supplementary Fig. 68. ^{1}H–^{1}H COSY of **3qa**, recorded at ~25 °C in $CDCl_3$ at 500 MHz.

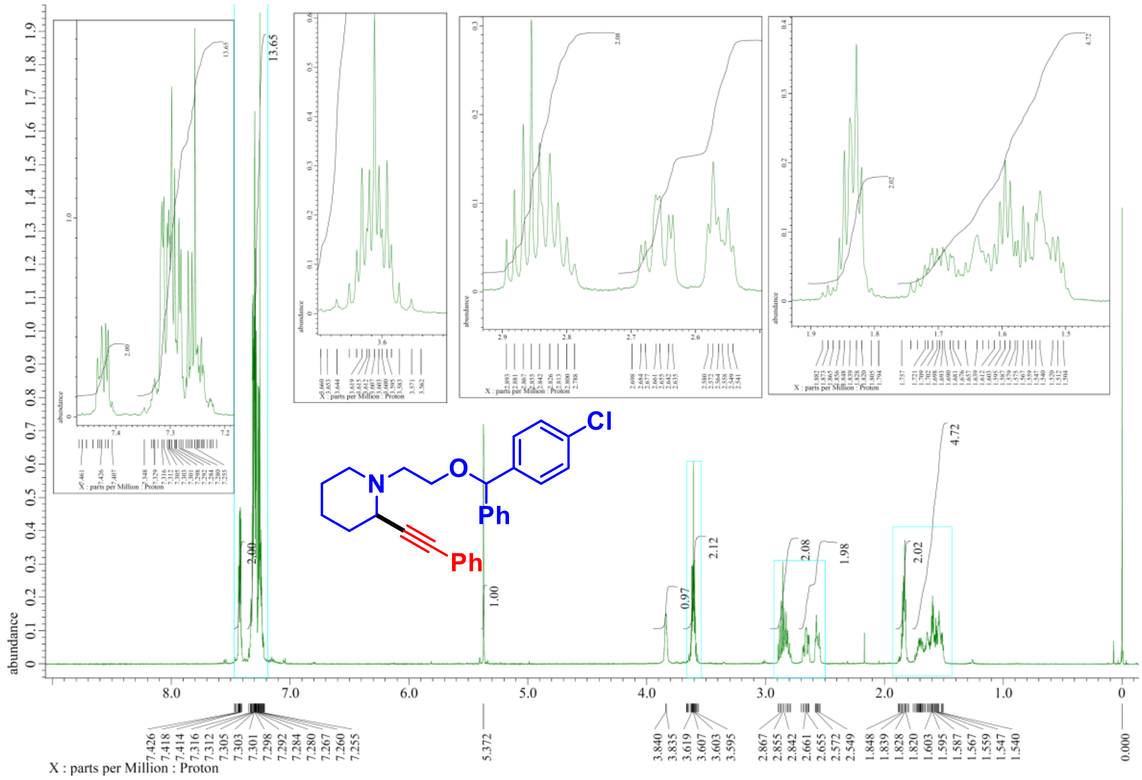

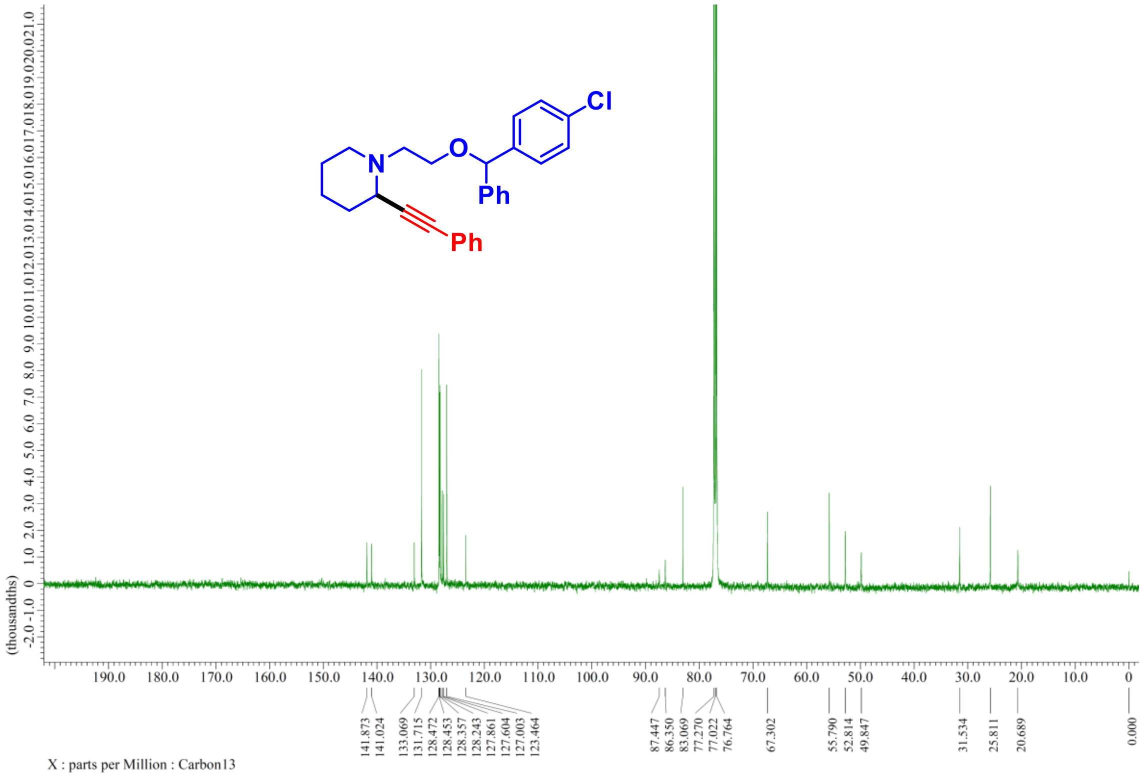

Supplementary Fig. 69. ^{1}H NMR and ^{13}C NMR of **3ra**, recorded at ~25 °C in $CDCl_3$ at 500 MHz and 126 MHz, respectively.

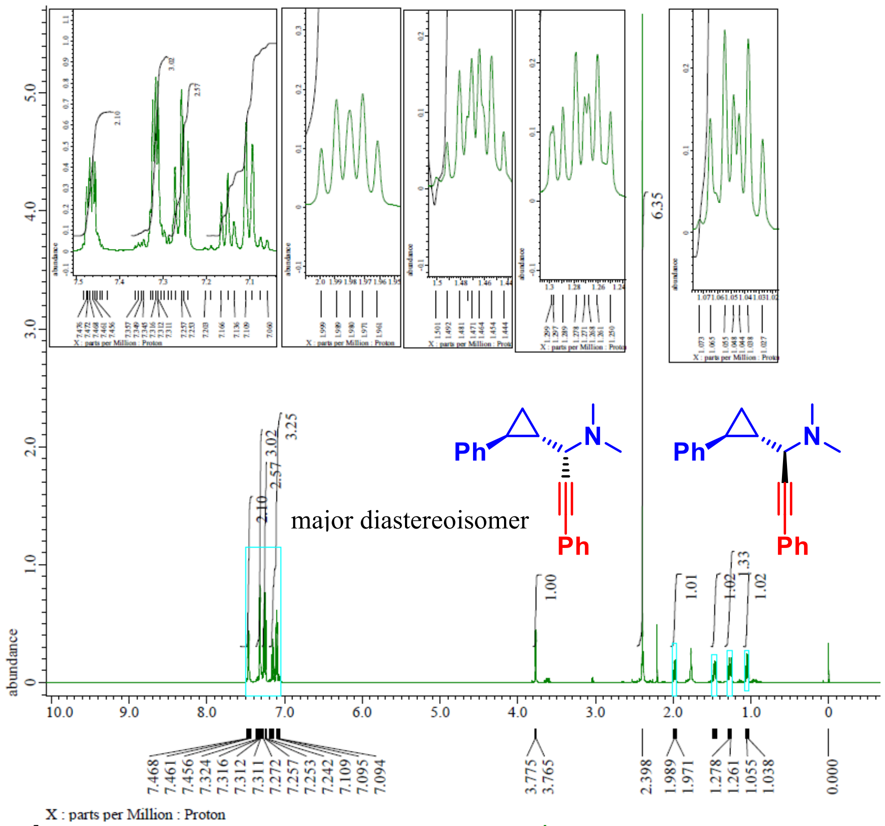

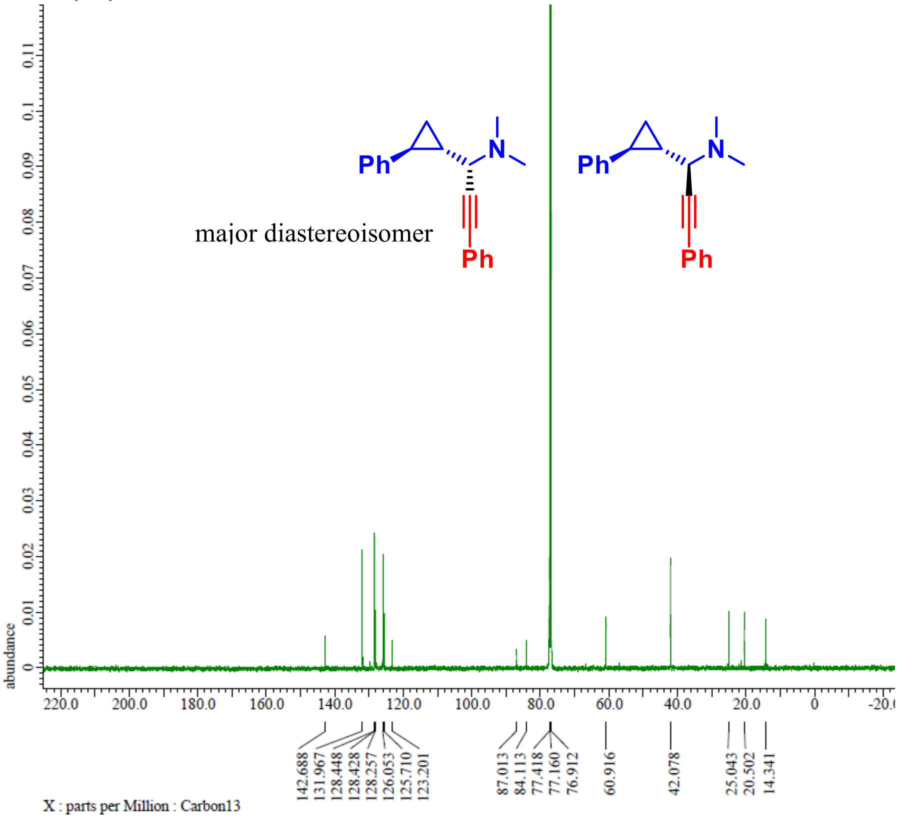

Supplementary Fig. 70. ^{1}H NMR and ^{13}C NMR of **3sa** (major diastereoisomer), recorded at ~25 °C in $CDCl_3$ at 500 MHz and 126 MHz, respectively.

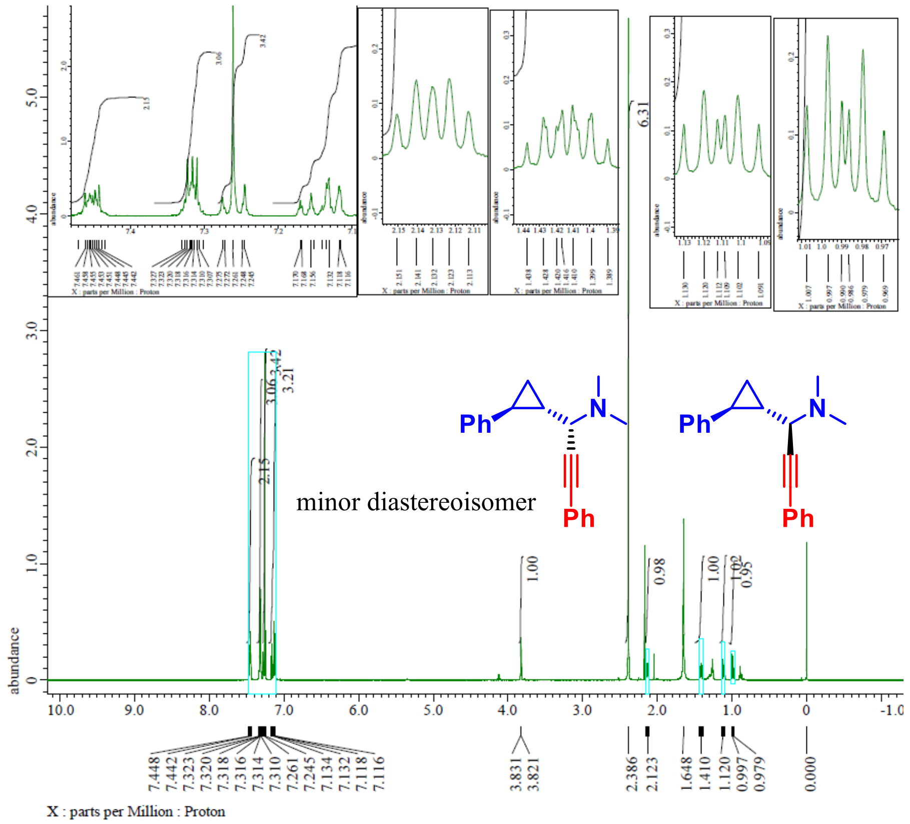

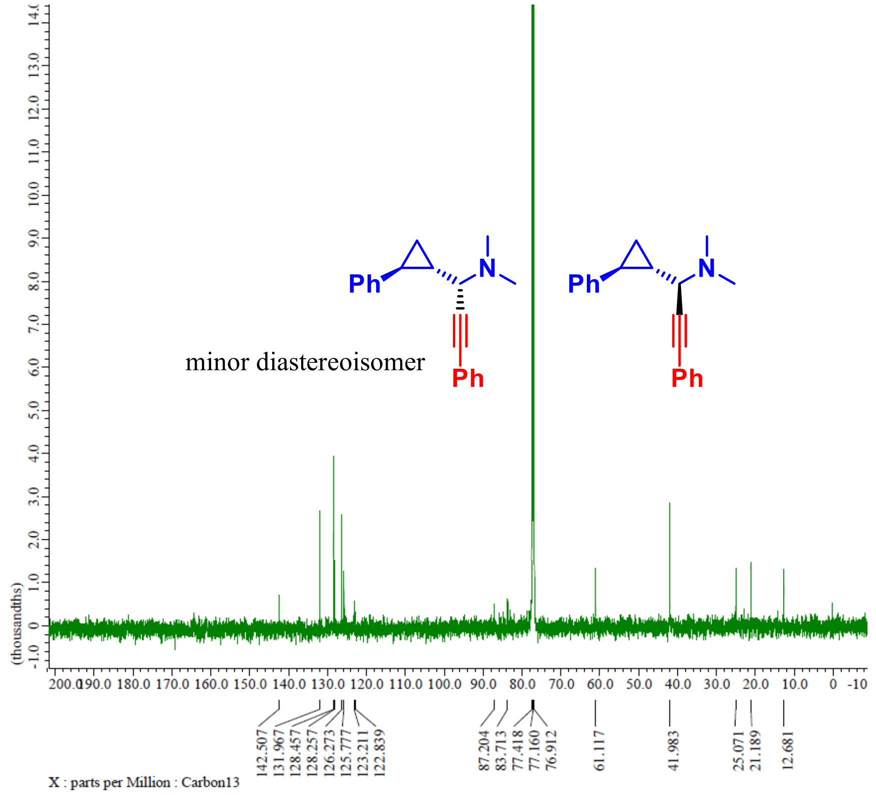

Supplementary Fig. 71. ^{1}H NMR and ^{13}C NMR of **3sa** (minor diastereoisomer), recorded at ~25 °C in $CDCl_3$ at 500 MHz and 126 MHz, respectively.

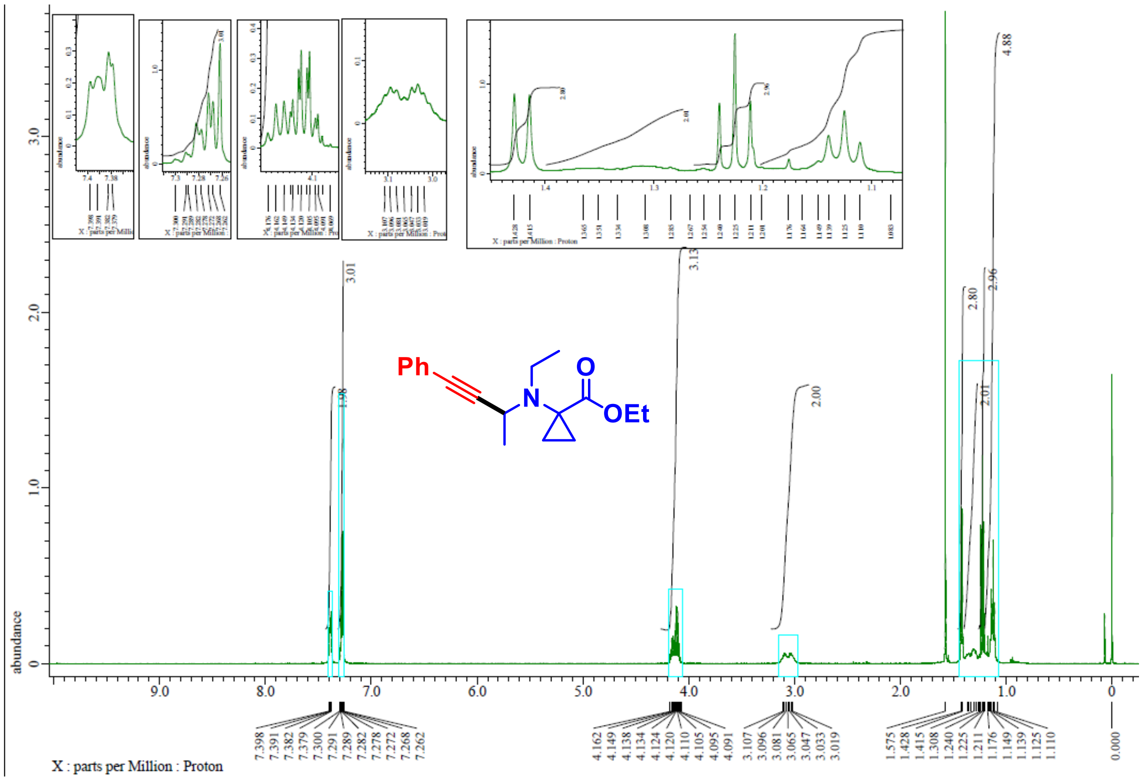

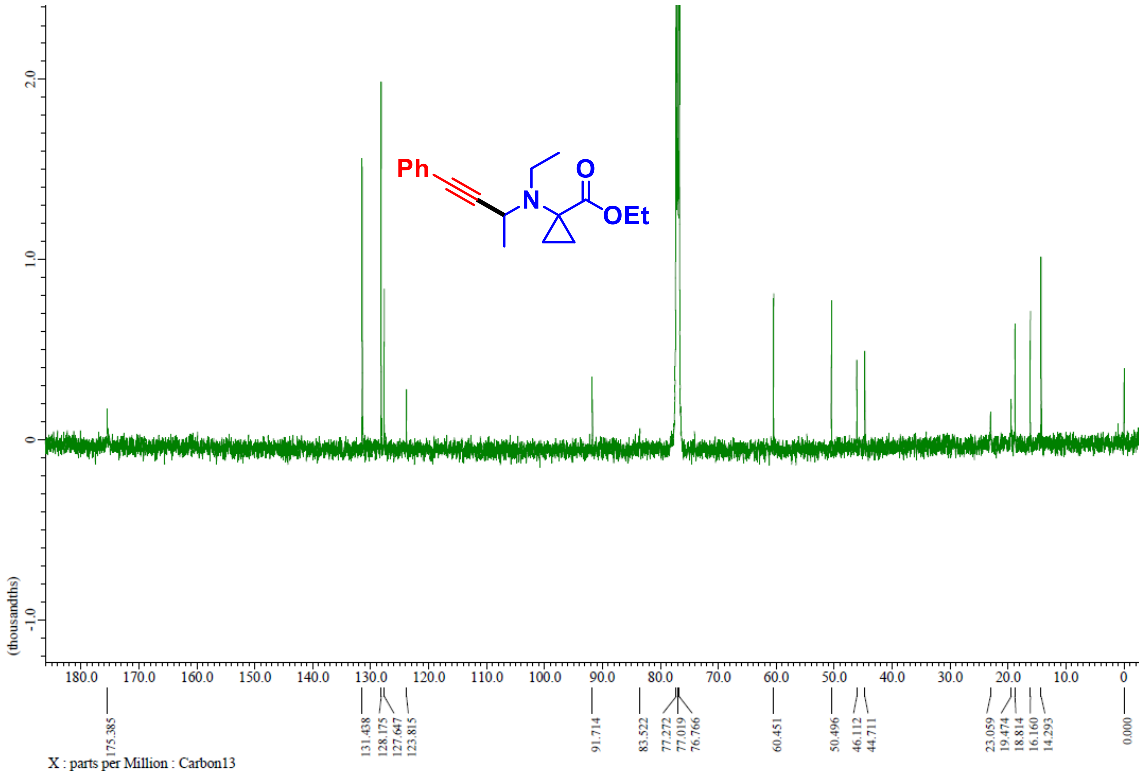

Supplementary Fig. 72. ^{1}H NMR and ^{13}C NMR of **3ta**, recorded at ~25 °C in $CDCl_3$ at 500 MHz and 126 MHz, respectively.

Supplementary References

S1. Frisch, M. J., Trucks, G. W., Schlegel, H. B., Scuseria, G. E., Robb, M. A., Cheeseman, J. R., Scalmani, G., Barone, V., Petersson, G. A., Nakatsuji, H., Li, X., Caricato, M., Marenich, A. V., Bloino, J., Janesko, B. G., Gomperts, R., Mennucci, B., Hratchian, H. P., Ortiz, J. V., Izmaylov, A. F., Sonnenberg, J. L., Williams-Young, D., Ding, F., Lipparini, F., Egidi, F., Goings, J., Peng, B., Petrone, A., Henderson, T., Ranasinghe, D., Zakrzewski, V. G., Gao, J., Rega, N., Zheng, G., Liang, W., Hada, M., Ehara, M., Toyota, K., Fukuda, R., Hasegawa, J., Ishida, M., Nakajima, T., Honda, Y., Kitao, O., Nakai, H., Vreven, T., Throssell, K., Montgomery, J. A., Jr., Peralta, J. E., Ogliaro, F., Bearpark, M. J., Heyd, J. J., Brothers, E. N., Kudin, K. N., Staroverov, V. N., Keith, T. A., Kobayashi, R., Normand, J., Raghavachari, K., Rendell, A. P., Burant, J. C., Iyengar, S. S., Tomasi, J., Cossi, M., Millam, J. M., Klene, M., Adamo, C., Cammi, R., Ochterski, J. W., Martin, R. L., Morokuma, K., Farkas, O., Foresman, J. B. & Fox, D. J. *Gaussian 16, Gaussian, Inc., Wallingford CT* (2016).

S2. Becke, A. D. Density-functional thermochemistry. III. The role of exact exchange. *J. Chem. Phys.* **98**, 5648–5652 (1993).

S3. Lee, C. T., Yang, W. T. & Parr, R. G. Development of the Colle-Salvetti correlation-energy formula into a functional of the electron density. *Phys. Rev. B* **37**, 785–789 (1988).

S4. Zhao, Y. & Truhlar, D. G. The M06 suite of density functionals for main group thermochemistry, thermochemical kinetics, noncovalent interactions, excited states, and transition elements: two new functionals and systematic testing of four M06-class functionals and 12 other functionals. *Theor. Chem. Acc.* **120**, 215−241 (2008).

S5. Andrae, D.; Häußermann, U.; Dolg, M.; Stoll, H. & Preuß, H. Energy-adjusted ab initio pseudopotentials for the second and third row transition elements. *Theor. Chim. Acta* **77**, 123−141 (1990).

S6 Hehre, W. J.; Ditchfield, R. & Pople, J. A. Self–consistent molecular orbital methods. XII. Further extensions of gaussian–type basis sets for use in molecular orbital studies of organic molecules. *J. Chem. Phys.* **56**, 2257−2261 (1972).

S7. Cabello, N., Kizirian, J. C., Gille, S, Alexakis, A., Bernardinelli, G., Pinchard, L. & Caille, J. C. Simple 1,2-diamine ligands for asymmetric addition of aryllithium reagents to imines. *Eur. J. Org. Chem.* 4835–4842 (2005).

S8. Fish, I., Stößel, A., Eitel, K., Valant, C., Albold, S., Huebner, H., Möller, D., Clark, M. J., Sunahara, R. K., Christopoulos, A., Shoichet, B. K. & Gmeiner, P. Structure-based design and discovery of new M_2 receptor agonists. *J. Med. Chem.* **60**, 9239–9250 (2017).

S9. Arvidsson, L.-E., Johansson, A. M., Hacksell, U., Nilsson, J. L. G., Svensson, K., Hjorth, S., Magnusson, T., Carlsson, A., Lindberg, P., Andersson, B., Sanchez, D., Wikstrom, H. & Sundell, S. *N,N*-Dialkylated monophenolic trans-2-phenylcyclopropylamines: novel central 5-hydroxytryptamine-receptor agonists. *J. Med. Chem.* **31**, 92–99 (1988).

S10. Marson, C. M., Khan, A. & Porter, R. A. Stereocontrolled formation of epoxy peroxide functionality appended to a lactam ring. *J. Org. Chem.* **66**, 4771-4775 (2001).

S11. Alonso, F., Moglie, Y., Radivoy, G. & Yus, M. Multicomponent click synthesis of 1,2,3-triazoles from epoxides in water catalyzed by copper nanoparticles on activated carbon. *J. Org. Chem.* **76**, 8394–8405 (2011).

S12. Jovel, I., Prateeptongkum, S., Jackstell, R., Vogl, N., Weckbecker, C. & Beller, M. α-Functionalization of non-activated aliphatic amines: ruthenium-catalyzed alkynylations and alkylations. *Chem. Commun.* **46**, 1956–1958 (2010).

S13. Chatterjee, P., Wang, H., Manzano, J. S., Kanbur, U., Sadow, A. D. & Slowing I. I. Surface ligands enhance the catalytic activity of supported Au nanoparticles for the aerobic α-oxidation of amines to amides. *Catal. Sci. Technol.* **12**, 1922–1933 (2022).

S14. Ma, L., Shi, X., Li, X. & Shi, D. Iron-catalyzed alkynylation of tertiary aliphatic amines with 1-iodoalkynes to synthesize propargylamines. *Org. Chem. Front.* **5**, 3515–3519 (2018).

S15. Mcnally, A., Prier, C. K. & MacMillan, D. W. C. Discovery of an α-amino C–H arylation reaction using the strategy of accelerated serendipity. *Science* **334**, 1114–1117 (2011).

S16. Chen, C.-K., Hortmann, A. G. & Marzabadi, M. R. ClO_2 oxidation of amines: synthetic utility and a biomimetic synthesis of elaeocarpidine. *J. Am. Chem. Soc.* **110**, 4829–4831 (1988).

S17. Yoshimitsu, T., Matsuda, K., Nagaoka, H., Tsukamoto, K. and Tanaka, T. Radical fixation of functionalized carbon resources: α-sp^3C−H carbamoylation of tertiary amines with aryl isocyanates. *Org. Lett.* **24**, 5115–5118 (2007).

S18. Sud, A., Sureshkumar, D. & Klussmann, M. Oxidative coupling of amines and ketones by combined vanadium- and organocatalysis. *Chem. Commun.* 3169–3171 (2009).

S19. Deb, M. L., Dey, S. S., Bento, I., Barros, M. T. & Maycock, C. D. Copper-catalyzed regioselective intramolecular oxidative α-functionalization of tertiary amines: an efficient synthesis of dihydro-1,3-oxazines. *Angew. Chem. Int. Ed.* **52**, 9791–9795 (2013).

S20. Sølvhøj, A., Ahlburg, A. & Madsen, R. Dimethylzinc-initiated radical coupling of β-bromostyrenes with ethers and amines. *Chem. Eur. J.* **21**, 16272–16279 (2015).

S21. Ueno, R., Ikeda, Y. & Shirakawa, E. *tert*-Butoxy-radical-promoted α-arylation of alkylamines with aryl halides. *Eur. J. Org. Chem.* 4188–4193 (2017).

S22. Shen, Y., Funez-Ardoiz, I., Schoenebeck, F. & Rovis T. Site-selective α-C–H functionalization of trialkylamines via reversible hydrogen atom transfer catalysis. *J. Am. Chem. Soc.* **143**, 18952–18959 (2021).

S23. Chiba, T. & Takata, Y. Anodic cyanation of tertiary aliphatic and heterocyclic amines. *J. Org. Chem.* **42**, 2973–2977 (1977).

S24. Yilmaz, O., Oderinde, M. S. & Emmert, M. H. Photoredox-catalyzed C_α–H cyanation of unactivated secondary and tertiary aliphatic amines: late-stage functionalization and mechanistic studies. *J. Org. Chem.* **83**, 11089–11100 (2018).

S25. Moriarty, R. M., Vaid, R. K. & Duncan, M. P. Hypervalent iodine oxidation of amines using iodosobenzene: synthesis of nitriles, ketones and lactams. *Tetrahedron Lett.* **29**, 6913–6916 (1988).

S26. Griffiths, R. J., Burley, G. A. & Talbot, E. P. A. Transition-metal-free amine oxidation: a chemoselective strategy for the late-stage formation of lactams. *Org. Lett.* **19**, 870–873 (2017).

S27. Jin, X., Kataoka, K., Yatabe, T., Yamaguchi, K. & Mizuno, N. Supported gold nanoparticles for efficient α-oxygenation of secondary and tertiary amines into amides. *Angew. Chem. Int. Ed.* **55**, 7212–7217 (2016).

S28. Miyazaki, R., Jin, X., Yoshii, D., Yatabe, T., Yabe, T., Mizuno, N., Yamaguchi, K. & Hasegawa, J. Mechanistic study of C–H bond activation by O_2 on negatively charged Au clusters: α,β-dehydrogenation of 1-methyl-4-piperidone by supported Au catalysts. *Catal. Sci. Technol.* **11**, 3333−3346 (2021).
